# Reaction kinetics and interplay of two different surface states on hematite photoanodes for water oxidation

**DOI:** 10.1038/s41467-020-20510-8

**Published:** 2021-01-11

**Authors:** Jingguo Li, Wenchao Wan, Carlos A. Triana, Hang Chen, Yonggui Zhao, Christos K. Mavrokefalos, Greta R. Patzke

**Affiliations:** Department of Chemistry, University of Zurich, Winterthurerstrasse 190, CH-8057 Zurich, Switzerland

**Keywords:** Chemistry, Catalysis, Electrocatalysis, Photocatalysis

## Abstract

Understanding the function of surface states on photoanodes is crucial for unraveling the underlying reaction mechanisms of water oxidation. For hematite photoanodes, only one type of surface states with higher oxidative energy (S1) has been proposed and verified as reaction intermediate, while the other surface state located at lower potentials (S2) was assigned to inactive or recombination sites. Through employing rate law analyses and systematical (photo)electrochemical characterizations, here we show that S2 is an active reaction intermediate for water oxidation as well. Furthermore, we demonstrate that the reaction kinetics and dynamic interactions of both S1 and S2 depend significantly on operational parameters, such as illumination intensity, nature of the electrolyte, and applied potential. These insights into the individual reaction kinetics and the interplay of both surface states are decisive for designing efficient photoanodes.

## Abstract (web summary)

Detailed understanding of the reaction kinetics of the surface states of hematite photoanodes is crucial to improving their water oxidation performance. Here the authors reveal two dynamically interacting states which play a key part in the structure properties of these anodes.

## Introduction

Direct solar energy to chemical fuels conversion through photoelectrochemical (PEC) approaches has attracted great research interest over the past decades^1–4^. It is generally accepted that the surface states on photoelectrodes are key determinants of the observed device performance^5,6^. Taking hematite photoanode as an example, two different kinds of surface states were widely probed primarily through electrochemical methods, photo-electrochemical impedance spectroscopy, and fast cathodic cyclic voltammetry (CV), in particular^7–10^. We notice that only the surface state with higher oxidative energy (S1) has been interpreted as the reaction intermediate, which was then determined as iron-oxo species with operando spectroscopic characterizations^11,12^. The other surface state with lower oxidative energy (S2), however, was assigned either to recombination centers or catalytically inactive sites^8,9,13^. Correspondingly, intensive research interests are directed on strategies to eliminate these detrimental surface states, including the addition of passivation layers^14,15^, secondary annealing^16^, and modification with cocatalysts^17–19^, etc. Despite the fact that such post-treatments can raise the PEC performance, many different mechanisms are proposed beyond a simple “passivation” hypothesis^20,21^. This is due to alterations of the original surface energetics which influence kinetic processes of the charge carriers^8,22^.

**Fig. 1:**
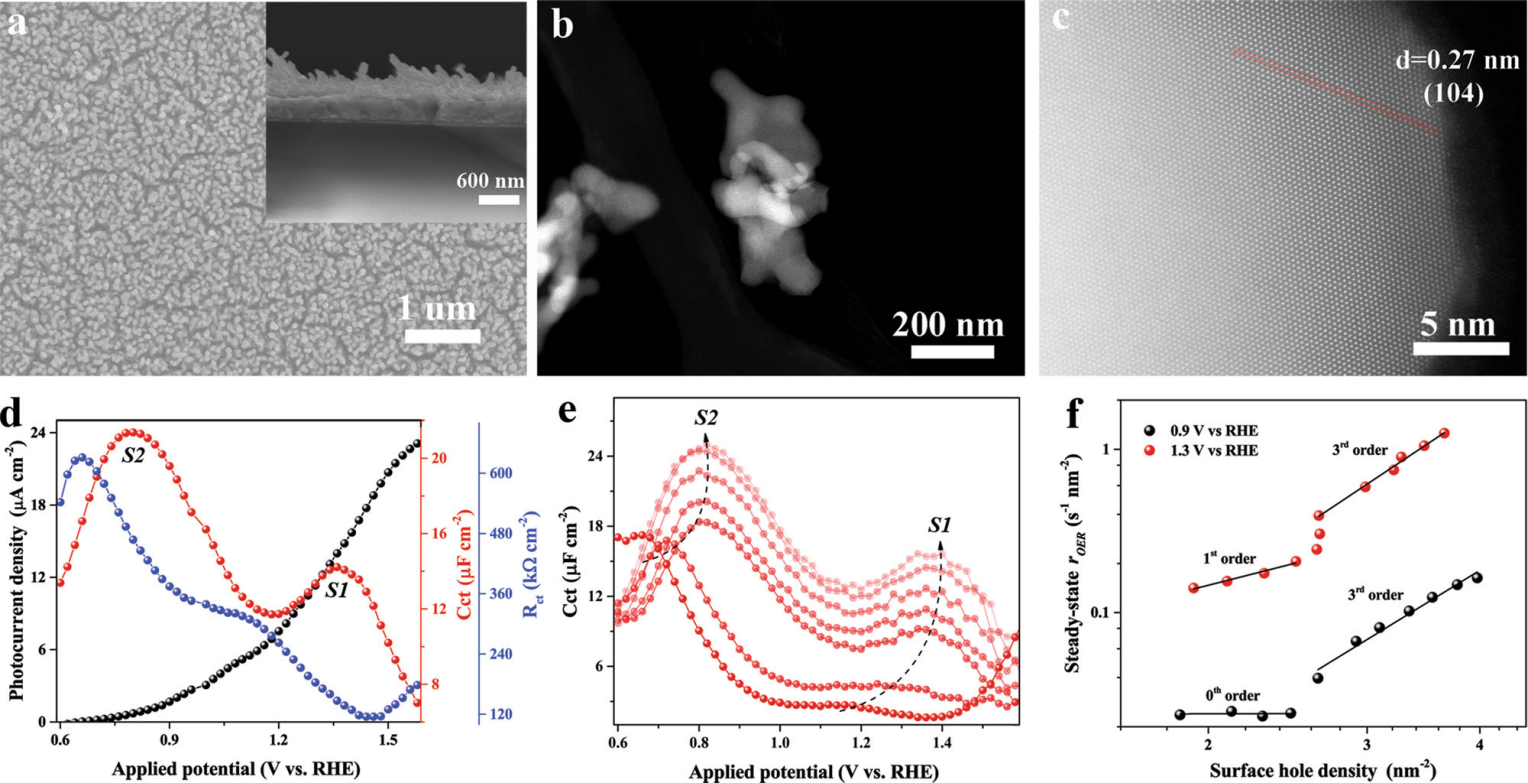
Electron microscopic analyses of hematite photoanode and reaction kinetics of S1 and S2 at pH 8.0. **a** Representative top-view SEM image and cross-section (inset) of as-prepared hematite thin film, **b** HAADF-STEM image of hematite nanorods, **c** magnified image with atomic resolution, **d** *J–V* (black) and surface state capacitance (*C*_ct_, red) and charge transfer resistance (*R*_ct_, blue) evolution curves as a function of the applied potential (illumination intensity: 100 mW cm^−2^, pH 8.0), **e** evolution of surface state capacitance at stepwise-elevated illumination density (5, 8, 10, 20, 40, 60, and 80 mW cm^−2^, pH 8.0), the growth of S1 and S2 is indicated by arrows, **f** rate law analysis at 1.3 and 0.9 V vs. RHE (pH 8.0) where S1 and S2 reach their maximum values. Charge transfer capacitance and corresponding resistance *R*_ct_ values were obtained by fitting the measured data (Supplementary Fig. S5) with an equivalent circuit shown in Supplementary Fig. S6. Steady-state OER rate (*r*_OER_) and surface hole densities were probed by intensity-modulated PEIS.

In this work, we first confirmed both types of surface states on model hematite surfaces. For brevity, we here refer to the first type of surface state appearing at high potential as S1 and to the second one at lower potential as S2. We notice that a consistent assignment of iron-oxo species could be achieved for S1, while the possibility that S2 is a reaction intermediate cannot be absolutely excluded for two reasons: (i) we do observe a low but definitely positive steady-state photocurrent when S2 reaches its maximum density (Fig. 1d) and (ii) although S2 is lower in energy, reaction kinetics need to be taken into consideration especially at higher external applied potentials. Coincidentally, a low activation energy reaction pathway (third-order reaction of surface holes) was reported on hematite surfaces where an iron peroxo intermediate was proposed with the support of density functional theory (DFT) calculations^23,24^. The energy barrier of this 3^rd^ order reaction mechanism is a few hundred meV lower than another first-order reaction mechanism. Unfortunately, the link between this peroxo species (probed by rate law analysis and DFT) and the widely reported surface states (S2 in the present work, probed by electrochemical methods) is still missing. We here newly link S2 to the peroxo species through a combination of electrochemical measurements and rate law analysis.

In addition, the fundamental questions “What is the correlation of both surface states?”, and “How do they behave under different experimental conditions?” still remain unresolved to a large extent. This issue significantly constrains the mechanistic understanding of PEC cells. We here propose a dynamic interaction of both surface states, where their density and distribution depend on a wide range of experimental conditions (applied potential, illumination intensity, electrolyte pH, etc.) In the following, we present detailed investigations of both surface states over a wide parameter range, using two complementary techniques. We derive their dynamic behavior from kinetic analyses and discuss it as a significant step towards the coherent understanding of PEC devices.

## Results

### Photoanode fabrication and characterization

Hematite photoanodes were prepared in a standardized manner according to previous protocols^25,26^. The morphology of hematite was first investigated using scanning electron microscopy, and uniform nanorod arrays were identified on the fluorine-doped tin oxide glass surface (Fig. 1a). The thickness is around 450 nm according to the cross-section image in Fig. 1a (inset). The hematite nanorods were further studied by scanning transmission electron microscopy (STEM) using a high-angle annular dark-field (HAADF) detector (Fig. 1b). The HAADF-STEM image in Fig. 1c shows high crystallinity, which was further confirmed with well-indexed thin-film X-ray diffraction patterns (Supplementary Fig. S1) and Raman peaks (Supplementary Fig. S2)^27^. As shown in Supplementary Fig. S3, an indirect band gap energy of 2.08 eV was determined from UV–vis absorbance data, which agrees with previously reported values^28,29^.

### Two types of surface states

First, the PEC performance of the fabricated photoanodes was evaluated under simulated solar irradiation. The near-steady-state photocurrent density (*J*, one minute preconditioned before data acquisition) vs. applied bias (*V*) curve under illumination in pH 8.0 electrolyte is shown in Fig. 1d (black spheres). As a reference, performance in the dark was recorded (Supplementary Fig. S4, black spheres). The *J–V* characteristics are in line with previous explorations with the onset potential positioned at around 0.8 V vs. RHE^10^. In order to uncover the underlying relationship between the observed *J–V* features and the charging of the surface states, PEIS measurements were carried out under the same conditions. Two semicircles are constantly present in the Nyquist plots at a wide range of applied biases (Supplementary Fig. S5), and the second semicircle (at low frequency) is generally becoming smaller at higher bias, suggesting a lower charge transfer resistance which is correlated well with the increasing current density (Fig. 1d). To quantify the associated two capacitive elements, a simplified physical model consisting of the space-charge capacitance and charge transfer capacitance (also referred to as chemical capacitance due to the charging of surface states) is considered (Supplementary Fig. S6), which has been well established for interpreting the hematite–electrolyte interface^30,31^. The surface state capacitance is caused by the accumulation of photo-generated minority carriers, i.e., holes in this case^5,9^. Two well-separated peaks with characteristic energetics are present in the tested bias range (Fig. 1d, red spheres), one around the photocurrent onset (S2), and another one at a higher potential of a few hundreds of millivolts (S1), which is in line with previous investigations on hematite surfaces, except for their absolute values^8,9,13,32,33^. In addition, a strong correlation between the accumulation of surface holes and the changes in charge transfer resistance could be identified (Fig. 1d, blue spheres)^10^.

In order to monitor how S1 and S2 evolve from dark (Supplementary Fig. S4) to one sun irradiation conditions (Fig. 1d), related PEIS measurements were conducted at stepwise-increased illumination intensities (Mott–Schottky analysis in Supplementary Fig. S7, donor densities, and flat band potentials are listed in Supplementary Table 1). As shown in Fig. 1e, the energetics and populations of both S1 and S2 show strong illumination intensity dependence, undergoing a rapid shift at around 8–10 mW cm^−2^. A strong correlation could be observed for peaks of S1 and S2 (Fig. 1e) and valleys in the charge transfer resistance plots occur for all different illumination intensities (Supplementary Fig. S8). Most likely, the sharp transition is a strong indicator for the change of catalytic characteristics of both S1 and S2. Therefore, understanding the dynamic nature of both types of surface states is crucial to decode the underlying reaction processes.

### Reaction kinetics of S1 and S2

To acquire information on the reaction kinetics of both surface states, we performed illumination intensity-modulated PEIS analyses (cf. Supplementary Discussion for details), where a series of different surface hole densities (derived using Supplementary Eq. 3, more details in the Supplementary Discussion I) and corresponding photocurrent densities could be obtained^34^. In this way, the reaction order (*n*) of surface holes and the related apparent reaction rate constant (*k*_app_) can be determined from rate law analyses, which provide key information for the reaction kinetics of both surface states.

At 1.3 V vs. RHE where S1 reaches peak values, as shown in Fig. 1f, characteristic first-order kinetics (*n* = 1.21, *k*_app_ = 0.08 s^−1^, *r*^2^ = 0.97) with respect to surface holes is present at weak illumination intensities. This first-order behavior at low hole densities is proposed to arise from water oxidation with one-hole oxidation processes as the rate determining step (RDS)^35,36^. We also note that previous PEIS analyses of hematite photoanodes have identified S1 at similar applied potential (~1.3 V vs. RHE)^9,10^. Spectro-electrochemical studies later confirmed high-valent iron-oxo intermediates (Fe^IV^ = O) to constitute S1, representing the one-hole transfer products^11,12^. Under higher illumination intensities, the reaction kinetics undergo a transformation to third-order (*n* = 3.36, *k*_app_ = 0.03 hole^−2^ nm^4^ s^−1^, *r*^2^ = 0.99, Fig. 1f) when the hole density increases to a certain threshold value (~2.6 holes per nm^−2^). Clearly, the OER rate is significantly accelerated due to the reaction order transition. The turnover frequencies (TOFs) of surface holes increase from 0.16 s^−1^ at ~2.0 holes per nm^−2^ (first-order kinetics) to 0.61 s^−1^ at ~3.0 holes per nm^−2^ (third-order kinetics), which is similar to previous studies^24,37^. Such phenomena have been explored on multiple n-type oxide semiconductor surfaces with the help of optical (photoinduced absorption spectroscopy, PIA) coupled electrochemical techniques^38,39^.

To determine the reaction kinetics of S2, a similar rate law analysis approach was adopted. At 0.9 V vs. RHE, an unexpected zero-order kinetics (*n* = 0.01, *k*_app_ = 0.03 hole nm^−2^ s^−1^, *r*^2^ = 0.95) with respect to surface holes was derived for the weak illumination range (Fig. 1f), which means that the initially accumulated S2 is hardly participating in the OER. The TOF for surface holes in the OER is merely 0.02 s^−1^ even at a density of ~2.0 holes per nm^−2^. Such a slow rate of hole transfer implies that these separated surface holes are undergoing significant recombination with conduction band electrons at the hematite–electrolyte interface, which is in line with observations in a previous study of Le Formal et al.^40^ (the competition of interfacial hole transfer with recombination is discussed further in the Supplementary Discussion II). However, once the surface hole density surpassed a certain value (as a result of increased illumination intensity), a distinct third order reaction (*n* = 3.09, *k*_app_ = 0.01 hole^−2^ nm^4^ s^−1^, *r*^2^ = 0.97) was present, indicating that S2 indeed became active in the OER. Unfortunately, the OER rate is still very limited despite the fast third-order kinetics. The TOF for surface holes increased to 0.08 s^−1^ at ~3.0 holes per nm^−2^, which is far below the value determined at 1.3 V vs. RHE, implying that recombination is still the prevailing option for surface holes. The concurrent observation of a third-order reaction of surface holes at both 0.9 and 1.3 V vs. RHE under intense illumination suggests that shared reaction intermediates, most likely iron-peroxo species, are probably involved in both cases. This hypothesis is based on the facts that (i) the accumulation of S1 (iron-oxo species, first-order reaction pathway), could initiate a third-order reaction pathway by lowering the reaction activation energy;^22^ and (ii) S2 exhibits lower oxidative energetics compared to S1, around 400 mV difference on the potential scale (Fig. 1d, e), which is essentially close to the activation energy difference for the first- and third-order pathways^24^. Therefore, we tentatively assign S2 to iron-peroxo species generated after O–O bond formation (operando spectroscopic analysis is ongoing, details are discussed in the Supplementary Discussion III), and further analyses are provided below. Apparently, the presence and interplay of S1 and S2 follow specific dynamics, and understanding their relationship is the key to further interpret the reaction kinetics at the hematite surface.

### Interplay and lifetimes of S1 and S2

**Fig. 2:**
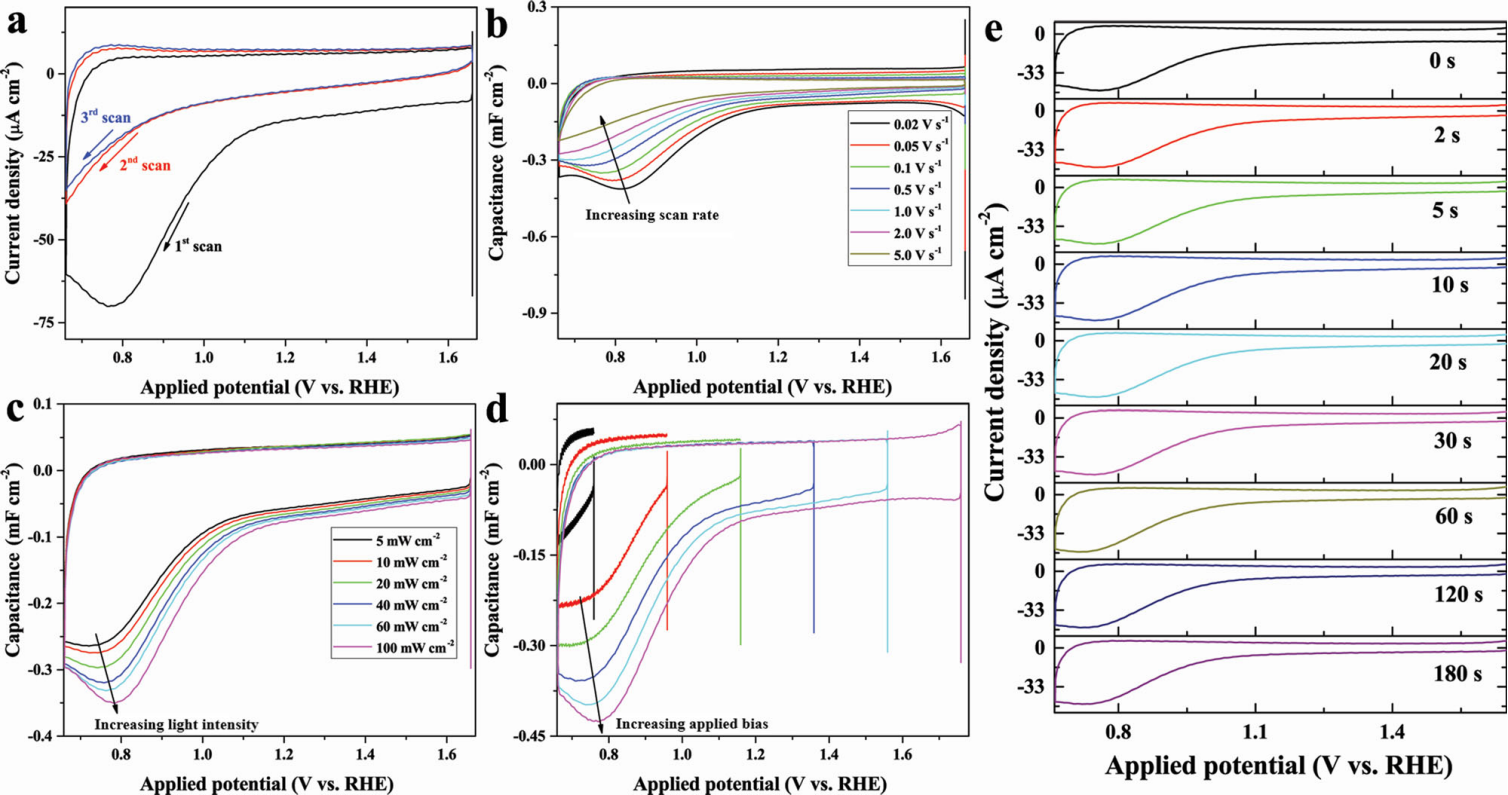
Evolution and lifetimes of S1 and S2. Fast cathodic CV scans (200 mV s^−1^, otherwise separately labeled) of a representative photoanode at pH 8.0 after preconditioning at steady-state photoelectrochemical OER (100 mW cm^−2^, otherwise separately labeled) at the high potential for 1 min: comparison of different scan cycles (**a**), the capacitive plot of the first CV scans measured at different scan rates (**b**), comparison of different illumination intensities (**c**), different potentials (**d**), and different waiting times under dark conditions (**e**).

Both S1 and S2 should in principle display defined reduction bands during the cathodic sweep in the CV if they have sufficient lifetime and populations on the surface. Therefore, the photoanode was first kept at OER potential under illumination (one minute) to reach a steady-state, and then a fast cathodic CV scan in the dark was conducted (details in the Supplementary Discussion). As shown in Fig. 2a, only one reduction band corresponding to the reduction of S2 was present during the first scan. This means that S2 has a sufficient lifetime for CV analysis while S1 does not. During the following scans in dark conditions, S2 also disappeared and this is clearly pointing to the fact that it is a reaction intermediate and only involved in the PEC process. To track the evolution of S2, the above CV measurements were furthermore performed at different scan rates. In the capacitance plots as shown in Fig. 2b, the amount of S2 decreases as the scan rate increases, indicating that its population grows over time. This is a rather unexpected phenomenon because it is normally anticipated that surface charged states would be reduced or recombined with increasing time in the dark.

The growth of S2 over time (Fig. 2b) suggests that most probably a certain amount of S1 has transformed into S2. This could be assisted by understanding the physiochemical processes associated with the fast cathodic CV scan experiment. Initially, both S1 and S2 have reached steady-state density as the photo-excitation continues for 1 min. Once the light is off, the incident beam goes to zero intensity on the ms timescale (determined by the response time of the beam shutter), and the supply of S1 is stopped. The resting S1 which is located at higher oxidative potential can now follow different pathways, such as recombination with conduction band electrons, direct initiation of OER, or transformation into low energy S2. This partial conversion process is also supported by the fact that the population of S2 grows when either the starting illumination intensity (Fig. 2c) or the potential (Fig. 2d) is increased during the initial preconditioning stage of the fast cathodic CV measurements. In addition, it is clear that there is already a certain level of hole accumulation at the S2 sites even at low illumination intensities or low applied potentials (Fig. 2c, d). Therefore, we can exclude the possibility that the charge filling of S2 takes place after the charge saturation of S1. Instead, the charging of S1 and S2 is in equilibrium through electronic interactions.

In order to estimate the lifetime of S2, we designed the following experiments: after the photoanode was kept at OER potential for one minute (steady-state), the light was turned off and different waiting time intervals (while the applied potential was kept constant, Supplementary Fig. S9) were applied prior to the fast cathodic CV scan in the dark. The lifetime of S2 is surprisingly long as its reduction profiles overlapped even after a resting time of 3 min (Fig. 2e). Such a long-lived intermediate has been assigned to surface peroxo species^41–43^, which is also in line with our analysis above. Unfortunately, it is not possible to confirm the precise lifetime of S2 as the potential is constantly changing during the fast CV scan (cf. Supplementary Discussion for details).

### Effect of surface protonation state on the dynamic interplay of S1 and S2

**Fig. 3:**
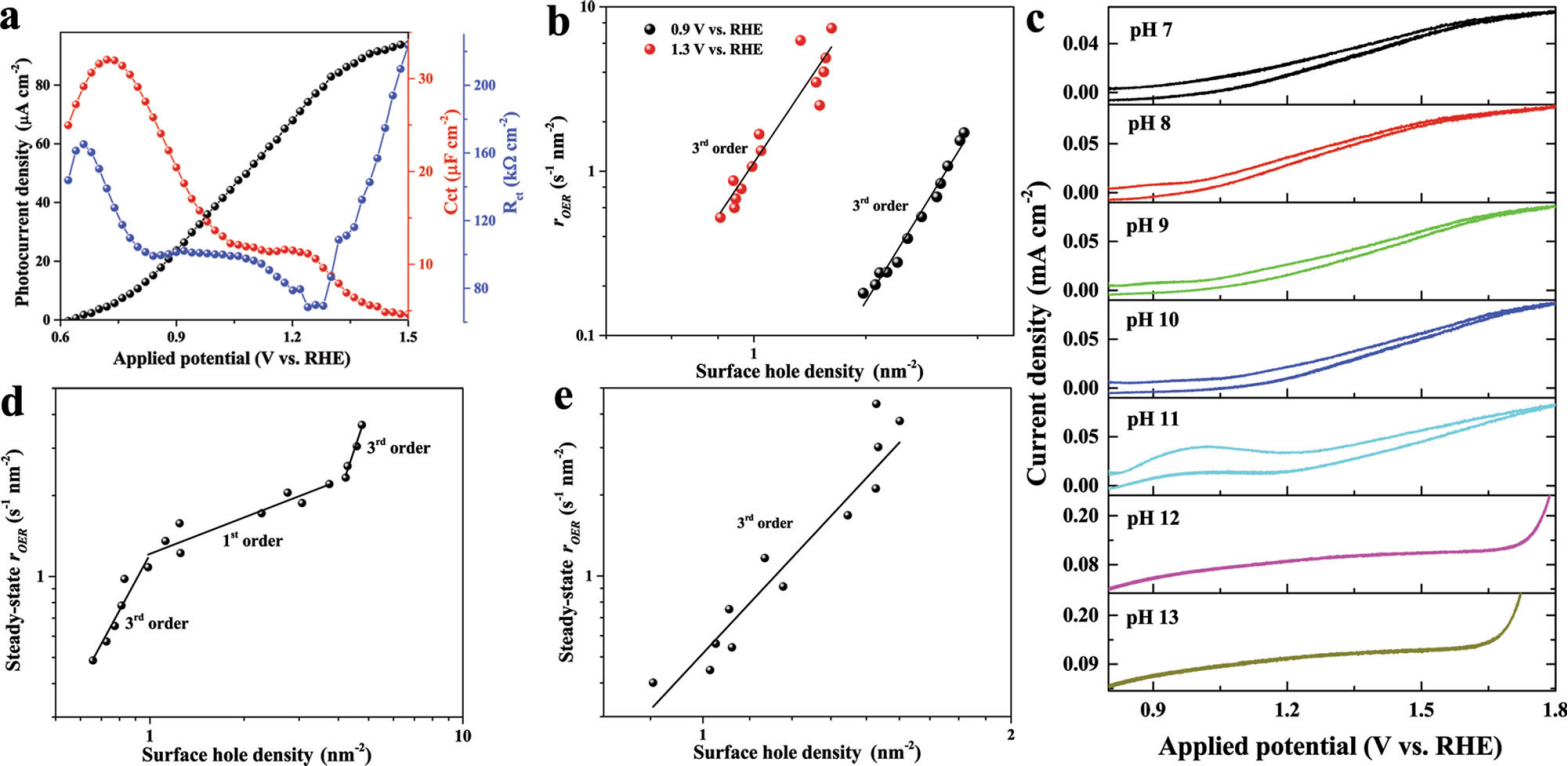
Effect of electrolyte pH on the dynamic interplay of S1 and S2. **a** Evolution of *J–V* (black), surface state capacitance (*C*_ct_, red) and charge transfer resistance (*R*_ct_, blue) as a function of the applied potential (illumination intensity: 100 mW cm^−2^, pH 13.0). **b** Rate law analysis at 1.3 and 0.9 V vs. RHE (pH 13.0) where S1 and S2 gain maximum values. **c** CV profiles (illumination intensity: 100 mW cm^−2^, scan rate: 5 mV s^−1^) of a hematite photoanode measured in different electrolyte pH (unbuffered). Rate law analysis at 1.3 V vs. RHE in pH 11.0 where S1 reaches maximum values without (**d**) and with buffer ions (**e**). Steady-state OER rate (*r*_OER_) and surface hole densities were probed by intensity-modulated PEIS.

To further understand the interplay between S1 and S2, similar PEIS measurements were performed at different pH values (Mott–Schottky analysis in Supplementary Fig. S10, donor densities and flat band potentials are listed in Supplementary Table 2). As shown in Fig. 3a, the ratio of S1 to S2 is suppressed at pH 13 and a much higher OER rate is achieved. Data obtained in 1 M NaOH (pH 13.6, Supplementary Fig. S11) and at other pH values (Supplementary Figs. S12–15) are also in line with this observation. More importantly, comparable energetic potentials of both S1 and S2 were determined regardless of the electrolyte pH (Supplementary Fig. S16), suggesting that the same reaction intermediates are present. In addition, the evolution of S2 at pH 13 was also evaluated by the fast cathodic CV scan experiments described above, and similar features were observed as a function of (i) scan cycles and scan rates (Supplementary Figs. S17 and S18), (ii) starting potentials (Supplementary Fig. S19), (iii) illumination intensities (Supplementary Fig. S20), and resting time in the dark (Supplementary Figs. S21 and S22). The decreased ratio of S1 to S2 at high pH means that the formation of S2 is now facilitated. This is substantiated by the uniform third-order reaction for the surface holes at both 0.9 (*n* = 3.37, *k*_app_ = 0.03 hole^−2^ nm^4^ s^−1^, *r*^2^ = 0.98) and 1.3 (*n* = 3.07, *k*_app_ = 1.32 hole^−2^ nm^4^ s^−1^, *r*^2^ = 0.92) V vs. RHE (Fig. 3b), which differs starkly from the kinetics observed at pH 8 (Fig. 1f). Specifically, the TOF of surface holes already reaches 0.70 s^−1^ at ~3.0 holes per nm^−2^ and exceeds 5.11 s^−1^ at only ~1.5 holes per nm^−2^ for 0.9 and 1.3 V vs. RHE, respectively. The absence of zero-order and first-order kinetics at both low and higher potentials under weak illumination could be attributed to the fast migration kinetics of S1.

Since the point of zero net charges (PZC) of hematite lies at around pH 11 for our device (Fig. 3c, and additional discussions in the Supplementary Discussion), the hematite surface would be mostly deprotonated at pH 13. Thus, there will be a significant amount of channels for S1 to hop and accumulate in the form of S2^44,45^, and the overall OER could proceed directly with higher reaction order of surface holes without much accumulation taking place. However, we still cannot completely exclude the possibility that the minimum surface hole density collected here is higher than the threshold points where the reaction order transition takes place (as the minimum illumination intensity is 5 mW cm^−2^ for our setup)^23^.

To further confirm the effect of surface protonation/deprotonation on the distribution of surface states, a similar rate law analysis was conducted near the pH of PZC. Surprisingly, at 1.3 V vs. RHE, the reaction order with respect to surface holes exhibits a unique three-stage transition, as shown in Fig. 3d, starting from a high reaction order (third, *n* = 2.73, *k*_app_ = 1.21 hole^−2^ nm^4^ s^−1^, *r*^2^ = 0.92), then proceeding to a lower reaction order (first, *n* = 0.78, *k*_app_ = 0.19 s^−1^, *r*^2^ = 0.90) and returning to higher reaction order (third, *n* = 3.28, *k*_app_ = 0.04 hole^−2^ nm^4^ s^−1^, *r*^2^ = 0.96) again. This three-stage reaction order switch was further verified at different potentials and electrolyte concentrations (Supplementary Figs. S31 and S32). We anticipate that the reaction proceeds with mostly deprotonated states first because the diffusion of hydroxyl ions to the photoanode surface is sufficient to sustain a small steady-state OER rate when the illumination is weak. In this case, S1 has sufficient mobility for the fast formation of S2 on the deprotonated surface, and the overall reaction starts with third-order regarding surface holes. As the steady-state OER rate grows with elevated illumination intensity, the supply of hydroxyl ions is most likely not able to sustain the deprotonated surface and the migration of S1 is limited so that first-order kinetics with respect to holes would be expected as discussed above. Once the density of S1 surpasses a certain threshold, the third-order reaction would be resumed as the formation of S2 is facilitated again. This interesting three-stage transition of reaction orders at pH 11.0 combines the observations in the strong alkaline electrolyte (pH 13.0, Fig. 3b) and in near-neutral conditions (pH 8.0, Fig. 1f). According to our analysis, the first reaction order transition (third to first) should be mainly attributed to the change of surface protonation state, while the second reaction order transition (first to third) is dominated by the effect of S1 accumulation. However, we have to make it clear that both factors, namely changes in surface protonation state and S1 accumulation, are coexisting at the semiconductor-electrolyte interface at pH 11.0. This implies that we are unable to completely exclude the contribution of S1 accumulation to the first reaction order transition (third to first). This also applies to the effect of changes in the protonation state on the second reaction order transition (first to third). Furthermore, this may explain the fact that the threshold values for the surface hole density of the reaction order transitions vary with the pH (Fig. 3b, d, Fig. 1f).

The importance of the surface protonation state is also validated by analyzing the transient photocurrent profiles in terms of (i) anodic decay half time (Supplementary Tables 3 and 4), (ii) steady-state photocurrent (Supplementary Figs. S24 and S27), and (iii) hole transfer efficiency (Supplementary Figs. S29 and S30) as a function of electrolyte pH and concentration, illumination density and applied potential (Supplementary Figs. S23, S25, S26, and S28). At 0.9 V vs. RHE, only third-order kinetics were observed near the pH of PZC, which means that the photoanode surface remains deprotonated at the applied illumination range. This is also supported by the fact that a very low steady-state OER rate is recorded (Supplementary Fig. S33).

In order to further highlight the importance of surface protonation/deprotonation for the distribution of S1 and S2, the above experiments were repeated in the buffered electrolyte. The ratio of S1 to S2 is already significantly and early lowered at pH 9, and completely disappears at higher pH values (Supplementary Figs. S34–40). This indicates that the drastic protonation/deprotonation change at the photoanode surface does not take place anymore (Supplementary Figs. S41–47 and Supplementary Tables 5 and 6). Therefore, the three-stage reaction order transition near the PZC vanished (Fig. 3e), indicating the productive role of buffer species for the migration and accumulation of S1.

### Reproduction of the above trends by transient photocurrent spectroscopy (TPS) and implications

In addition to PEIS, TPS is another classic (photo)electrochemical method for probing the density of surface states^46^. The evolution profile of surface states (Supplementary Fig. S48) largely mimics the features of our PEIS analysis (Fig. 1c). Similarly, the surface hole density can be calculated from TPS (details are included in the Supplementary Discussion), so that, upon illumination intensity modulation, we again used the rate law analysis approach to derive the kinetic parameters of the reaction.

Importantly, similar transition phenomena of the reaction order could be reproduced in the unbuffered electrolyte as obtained from the PEIS approach (Supplementary Figs. S49 and 50), especially the three-stage reaction order transition at PZC conditions (Supplementary Figs. S51 and S53). We notice that the observed reaction order transition here is generally in line with the results previously probed by coupled PIA and electrochemical (TPS) techniques^23,24,47^. This means that the kinetic behavior of surface states can be analyzed in a rather equivalent manner by different techniques^37,40^. However, it is important to know that these different probing techniques are based on very different working principles. For example, surface hole density values derived from the PEIS approach are based on the separation of electronic processes occurring at different frequency domains^48–50^. Since OER through surface states is very sluggish compared to charge separation and diffusion processes, it is possible to apply a simplified physical model to derive the capacitance that is responsible for the slow interface process^33,51^. Obviously, PEIS is counting the total number of surface holes resting in the form of both S1 and S2 at steady-state (cf. Supplementary Discussion for details) when assuming that there is no charge transfer through valence band holes. In contrast, the hole density derived from TPS is mostly based on short-lived S1 without S2 because it can rest for more than 3 min (Fig. 2e). The positive correlation between the measured surface hole density in TPS (Supplementary Fig. S48) and the resolved surface state capacitance in PEIS (Fig. 1c) is essentially the reason why we derived similar reaction orders from the rate law analyses. This actually points to the fact that the interconversion of surface holes (with different energies) follows some defined rules (cf. Supplementary Discussion for details). In the future, techniques that can first differentiate S1 from S2, and then individually investigate their evolution kinetics may provide more precise insight into their mutual influence and eventually shine a light on the still widely unknown interfacial reaction mechanisms^41,52^.

## Discussion

The behavior of surface states that are located at the solid–liquid interface is closely related to the operational conditions, such as applied potential, illumination intensity, and electrolyte pH. Obviously, all these experimental parameters will significantly influence not only the accumulation of photogenerated holes at the interface but also their distribution in the forms of S1 and S2. In order to investigate the intrinsic surface properties, we here employed native hematite as a model system (see details in the Supplementary Discussion IV). As shown in Supplementary Figs. S55–S60, the Faradaic efficiency of oxygen evolution on our hematite samples was almost unity at different pH values (8–13) and applied potentials of 1.3–1.6 V vs. RHE (see details in the Supplementary Discussion V).

**Fig. 4:**
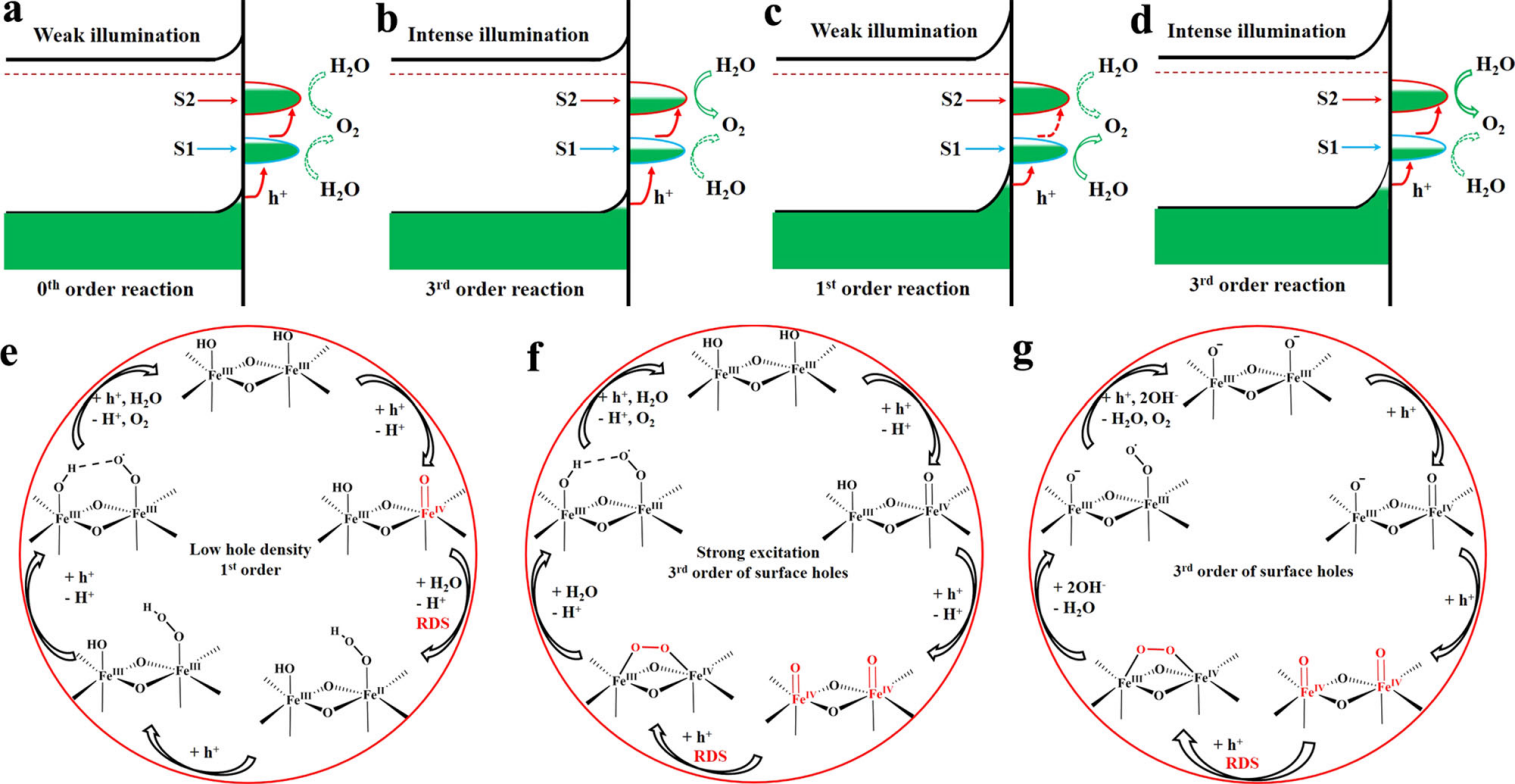
Schematic illustration of the steady-state interplay of S1 and S2 and corresponding catalytic cycles. Top: the steady-state interplay of S1 and S2 at different applied potentials and illumination intensities in pH 8.0 electrolyte. **a** At 0.9 V vs. RHE and weak illumination (<10 mW cm^−2^), a small fraction of photogenerated S1 is stored in the form of S2 in order to prevent recombination, while lesser amounts of accumulated S2 are unable to initiate OER effectively. This results in zero-order kinetics with respect to surface holes. **b** At 0.9 V vs. RHE and intense illumination (≥10 mW cm^−2^), S2 has achieved sufficient surface occupancy, so that the subsequent hole injection will give rise to OER. Third-order reaction kinetics of surface holes is observed. **c** At 1.3 V vs. RHE and weak illumination (<10 mW cm^−2^), photogenerated S1 is able to directly initiate OER as the recombination process is significantly retarded. This gives rise to first-order reaction kinetics with respect to surface holes. **d** At 1.3 V vs. RHE and intense illumination (≥10 mW cm^−2^), the accumulation of S1 promotes the fast formation of S2 at lower energy, so that OER is now proceeding via a low energy barrier third-order pathway (dashed arrows, very slow processes; solid arrows, fast processes). Bottom: Proposed catalytic cycles at pH 8 and 1.3 V vs. RHE **e** under weak illumination (<10 mW cm^−2^, low surface hole density), where the nucleophilic addition of water to the Fe^IV^ = O site represents the RDS, **f** under intense illumination (>10 mW cm^−2^, high surface hole density), where the third hole oxidation of the edge site Fe^IV^(=O)–O–Fe^IV^(=O) becomes the RDS. **g** However, at pH 13, the RDS is still associated with the third hole oxidation of the edge site Fe^IV^(=O)–O–Fe^IV^(=O) across the applied illumination intensity range (5–100 mW cm^−2^).

To probe the effect of a single experimental parameter, we made sure that all other parameters were constant. First, at 0.9 V vs. RHE and electrolyte pH 8, the OER rate does not respond to an increase of the illumination intensity (zero-order as indicated in Fig. 1f). This means that the detected S2 exhibits very sluggish OER behavior under these conditions. However, the density of S2 is increasing fast with the illumination intensity (Fig. 1e), suggesting that it starts to act as a kind of reservoir for photogenerated holes. This reservoir function is further evidenced by the fast cathodic CV scan experiments (Fig. 2b). When the reservoir level is low, the chance for further hole injection to initiate OER is negligible (Fig. 4a), giving rise to the zero-order for surface holes in the overall OER reaction (Fig. 1f). Once the reservoir level reaches a certain threshold under stronger illumination, the subsequent injection of photogenerated holes becomes effective (Fig. 4b), and the overall reaction order for surface holes changes to third-order (Fig. 1f). In other words, at low applied potential and weak illumination intensity, photogenerated holes (S1) are mostly isolated and easily deactivated through recombination with conduction band electrons^40,53,54^. Only those who succeed to form triply oxidized edge site clusters can persist through transformation into less energetic and stable S2. When more S1 species are produced at higher illumination intensity, the possibility of their successful association is higher and a larger number of less energetic S2 states are formed.

At 1.3 V vs. RHE and electrolyte pH 8, however, S1 has a significantly longer lifetime as the conduction band electrons are mostly retarded due to strong band bending^55,56^. In this case, S1 is able to initiate OER even at lower densities (i.e., when the illumination is weak, Fig. 4c), and the first-order reaction of surface holes is expected and indeed observed (Fig. 1f). At elevated illumination intensity, the accumulation of S1 enables the establishment of its dynamic equilibrium with triply oxidized edge site centers, followed by the sustained formation of S2 (Fig. 4d). Therefore, an overall third order reaction was observed (Fig. 1f). We proposed according OER mechanisms^24,41,47,57^ (Fig. 4e, f) and included relevant discussions in the Supplementary Information.

When an electrolyte with pH 13 is applied, the direct observation of third-order reactions at both 0.9 and 1.3 V vs. RHE (Fig. 3b) suggests a significantly enhanced S1 mobility, which is in stark contrast to the observations in pH 8 (Fig. 1f), highlighting the importance of protonation state on the dynamic interplay of S1 and S2. Further, the unique three-stage rate order transformations near PZC conditions (Fig. 3d), as well as their disappearance in buffered systems (Fig. 3e), support a different catalytic mechanism on the deprotonated surface (Fig. 4g). Since a negligible kinetic isotope effect was observed over a wide range of electrolyte pH (Supplementary Fig. S54), we assume that the RDS of the third-order kinetics in Fig. 4f, g is O–O bond formation^23,24^. All the above discussions point to the fact that in order to understand the origin of different catalytic performances, an in-depth investigation of the dynamic interplay of S1 and S2 is essential.

In this study, two different surface states of hematite, S1, and S2, are resolved at distinct oxidative potentials for constant illumination intensity and defined electrolyte pH. In addition to the well-resolved chemical nature of S1, we further assign S2 to another OER intermediate (iron-peroxo species) and identify the dynamic interplay of both surface states based on the following results obtained from a wide range of experimental conditions: (1) rate law analysis at higher potential (1.3 V vs. RHE) reveals a distinct first-order reaction kinetics for surface holes at low densities, while third-order kinetics are observed when they accumulate toward higher density. In contrast, a transition from zero to third-order reaction kinetics was observed at lower potential (0.9 V vs. RHE). (2) S2 was demonstrated to exhibit a surprisingly long lifetime (>3 min) and it originates from the accumulation of short-lived S1. (3) Surface deprotonation in a strongly alkaline electrolyte facilitated the mobility of S1 and induced third-order reaction kinetics involving S2. More importantly, a unique three-stage reaction mechanism transformation was first discovered due to changes in the surface protonation state near the PZC. (4) Reproducible rate law trends were obtained using TPS as an alternative technique at various pH values.

We newly connect and unify the conclusions of previous works based on a variety of different analytical techniques. We are, however, aware that the dynamic features of both S1 and S2 under different experimental conditions still render the unambiguous interpretation of their surface phenomena rather difficult. The present work also clearly highlights that different analytical techniques consequently probe different aspects of the interfacial catalytic reactions. This illustrates the increasing need for further comprehensive studies to tackle these current controversial issues of PEC research.

The kinetics and interplay of both surface states on hematite correspond well with many other oxide-based water oxidation catalysts monitored under operando conditions^52,58–60^. This points to high-energetic oxo species (after one-hole injection) as a common feature to initiate OER directly, with no need for extensive accumulation once they reach a sufficient lifetime. Such scenarios would then give rise to first-order reaction kinetics. Alternatively, the oxo species can quickly migrate and react with nearby oxo species to form less-energetic and long-lived peroxo species assisted by either stronger illumination or surface deprotonation, giving rise to higher reaction orders. This model is in line with the widely proposed edge motifs for Co-based water oxidation catalysts^41,44,61–63^. Based on the understanding of the dynamics and interplay of both surface states, strategies that facilitate oxo species migration on the surface would notably enhance the OER performance of hematite photoanodes. All in all, we introduce a clear strategy and convenient analytical techniques for understanding and customizing the surface properties of high-performance water oxidation catalysts through understanding their surface states^64,65^.

## Supplementary information

Supplementary Information

## Data Availability

The data that support the findings of this study are available from the corresponding author upon request.
