# Supplementary material for: Reaction kinetics and interplay of two different surface states on hematite photoanodes for water oxidation
Source: Nat Commun. 2021 Jan 11;12:255. doi: 10.1038/s41467-020-20510-8 (PMC7801602; doi:10.1038/s41467-020-20510-8)
Supplement: Supplementary file 1 — Supplementary Information [file 41467_2020_20510_MOESM1_ESM.pdf]

Supporting Information:

Reaction Kinetics and Interplay of Two Different Surface States on Hematite Photoanodes for Water Oxidation

Jingguo Li, Wenchao Wan, Carlos A. Triana, Hang Chen, Yonggui Zhao,

Christos K. Mavrokefalos, Greta R. Patzke*

Department of Chemistry, University of Zurich, Winterthurerstrasse 190, CH-8057 Zurich, Switzerland.

Correspondence and requests for materials should be addressed to G.R.P. (email: greta.patzke@chem.uzh.ch)

Table of Contents

1. Supplementary Methods 3

1.1 Materials and characterizations 3

1.2 Photoanode preparation 3

1.4 Band gap energy determination 5

2. Two types of surface states 6

2.1 Basic settings and physical model selection for photo-electrochemical impedance spectroscopy (PEIS) analysis 6

2.2 Illumination intensity modulated PEIS and rate law analysis 8

2.3 Tracing surface states with fast cathodic CV scans 10

2.5 Remarks on the lifetime of *S1* and *S2* 12

3. Distribution of both surface states in different electrolyte pH 13

3.1 PEIS analysis 13

3.2 Capturing surface states using fast cathodic CV scans at pH 13 17

3.3 Near steady-state CV analysis at different pH values 21

3.4 Transient photocurrent spectra as a function of illumination intensity 22

3.5 Transient photocurrent spectra as a function of applied potential 25

3.6 Hole transfer efficiency 27

3.7 Rate law analysis near the PZC 29

4. Distribution of both surface states in buffered electrolyte 30

4.1 PEIS analysis in buffered electrolyte 30

4.2 Near steady-state CV analysis in buffered electrolyte 35

4.3 Transient photocurrent spectra in buffered electrolyte as a function of illumination intensity 36

4.4 Transient photocurrent spectra in buffered electrolyte as a function of applied potential 39

4.5 Hole transfer efficiency in buffered electrolyte 41

4.6 Remarks on the TPS study in both buffered and unbuffered electrolyte at different pH values 42

5. Reproduction of results using transient photocurrent techniques 43

5.1 Profile of surface states probed by transient photocurrent techniques 43

5.3 Pathways for photogenerated holes 47

6. Proposed OER mechanism 47

7. Supplementary Discussion 48

7.1 Supplementary Discussion I: Density of surface holes for rate law analysis 48

7.2 Supplementary Discussion II: Competition of interfacial hole transfer with back-electron recombination (BER) 50

7.3 Supplementary Discussion III: Resolving the chemical nature of *S2* with operando spectroscopy 51

7.4 Supplementary Discussion IV: Reasons for the use of native hematite as a model system 52

7.5 Supplementary Discussion V: Faradaic efficiency of OER on hematite electrodes 53

8. References 59

1. Supplementary Methods

1.1 Materials and characterizations

Unless otherwise stated, all chemicals were of reagent grade and purchased from Sigma–Aldrich, and were used without further purification. The fluorine doped tin oxide (FTO) plates were sonicated for cleaning in: pure water, ethanol, acetone, pure water (15 min each). pH determination: Mettler Toledo FE 20 pH meter. UV-Vis spectroscopy: Perkin–Elmer Lambda 650 S spectrophotometer in the range 300-800 nm using a Quartz SUPRASIL precision cell (10 mm). Heating experiments: Nabertherm P330 (30-3000 °C). X-ray diffraction: Rigaku SmartLab X-Ray diffractometer equipped with a PhotonMax high-flux 9 kW rotating anode and X-ray source with Cu-K_α radiation at (λ = 1.54056 Å); HyPix-3000 detector; measured at 19 °C. Scanning electron microscopy (SEM): Zeiss Supra 50 VP. All photoelectrochemical measurements were carried out on a Zahner Zennium electrochemical workstation and data was analysed using the Thales software provided by Zahner. An AM 1.5 solar simulator (LOT QuantumDesign AG) was used for all photoelectrochemical tests; the illumination light intensity on the photoanode surface was controlled by the distance between light source and photoanode surface, and the illumination intensity was quantified using solar detector of LOT QuantumDesign AG. Front illumination geometry (from the sample side) was applied for all measurements. A mechanical beam shutter coupled with a K-cube Solenoid controller (Thorlabs GmbH) was used for modulating the solar irradiation, and it takes 10 ms for the shutter to open from 20% to 80%. A three-electrode cell was used with Ag/AgCl (in saturated KCl solution), Pt wire as reference and counter electrode respectively, 0.05 M $NaClO_4$ was employed as electrolyte and the pH value was tuned by adding different amounts of NaOH. Deuterated electrolytes were prepared at the same concentrations with D_2O (99.9 % purity). Kinetic isotope effects (KIE) were evaluated from the ratio of the steady-state photocurrents measured in H_2O and D_2O, respectively. 0.05 M borate buffer with different pH values was used for the control experiments. All voltages are normalized to the reversible hydrogen electrode (RHE) using: V (vs. RHE) = V (vs. Ag/AgCl) + 0.197 + 0.059 · pH. For PEIS tests, a frequency range from 0.1 to 10,000 Hz was selected with an AC voltage amplitude of 5 mV.

1.2 Photoanode preparation

The hematite photoanodes used in this study were fabricated according to previous work.[1,2]

1.3 Structural analysis

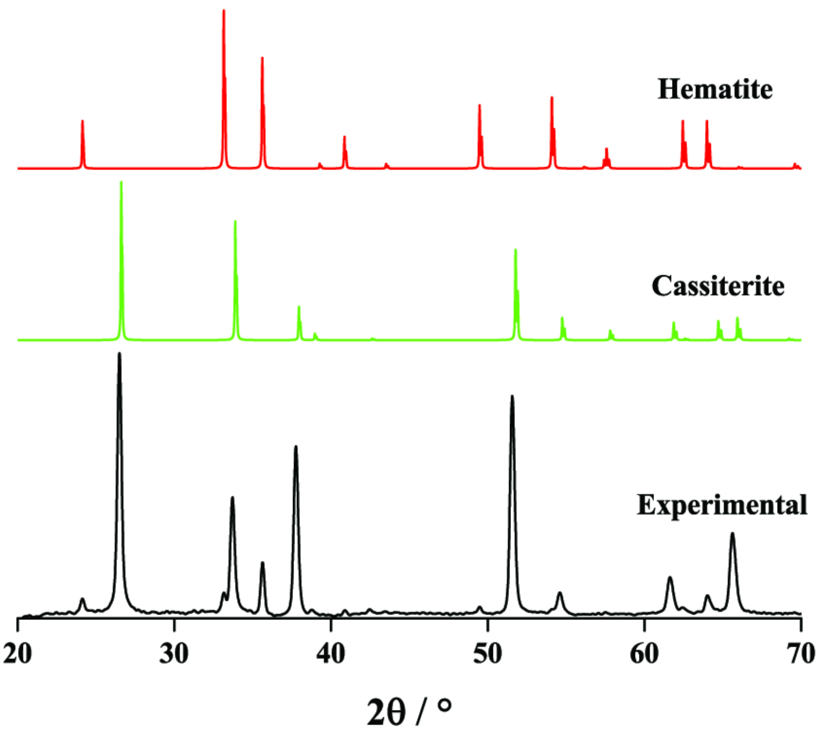

Supplementary Figure 1. Experimental thin-film XRD pattern of hematite photoanode (bottom), standard hematite (top, ICSD: 00-033-0664), and standard cassiterite (middle, ICSD: 00-041-1445).

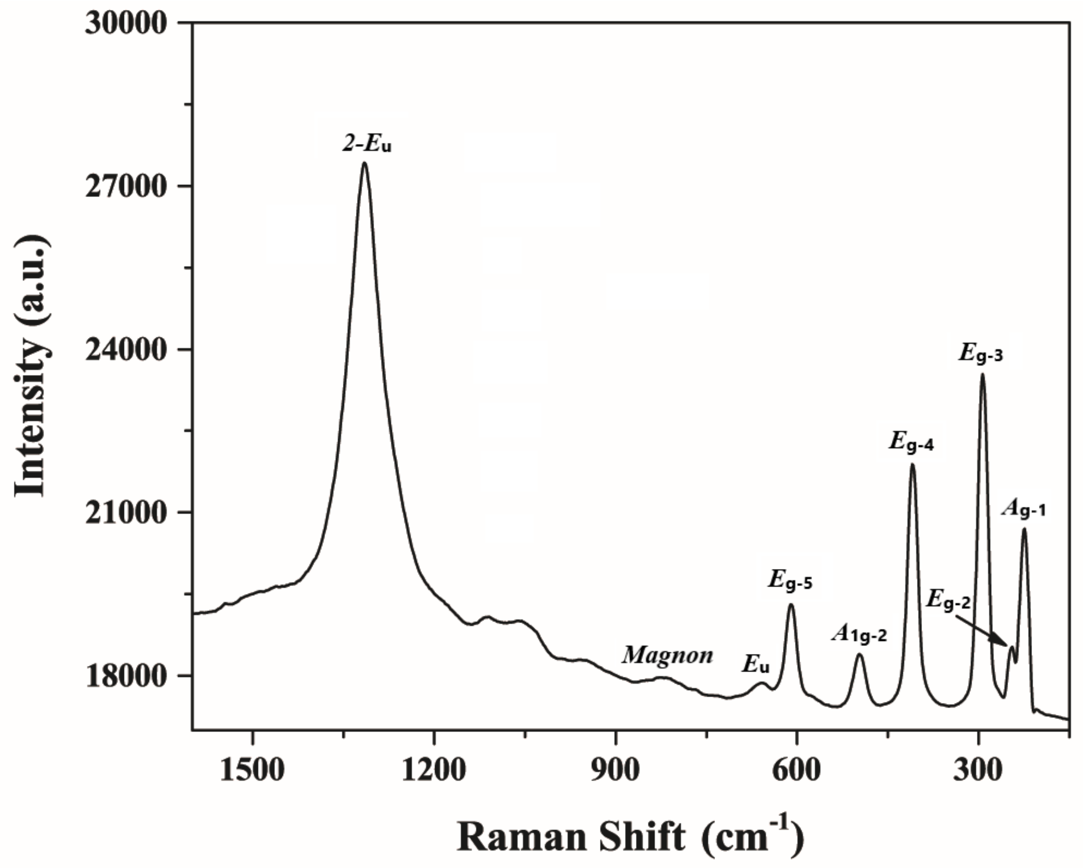

Supplementary Figure 2. Raman spectra of a representative as prepared hematite photoanode.

1.4 Band gap energy determination

The band gap energy of as-fabricated hematite was determined from the UV-vis absorbance data recorded in the range of 350-800 nm using the Tauc equation:

$$\alpha hv = A_0\left(hv - E_g\right)^n \qquad \textbf{Supplementary Equation 1}$$

where A_0 is a constant, hv is the photon energy (eV), and E_g is the band energy (eV). The value of n is related to the nature of the optical transition: n equals 0.5 or 1.5 for direct allowed/forbidden transitions, and n equals 2 or 3 for indirect allowed/forbidden transitions, respectively. α is the optical absorption coefficient, which can be derived from absorbance (A) and hematite film thickness (t) using the expression:

$$\alpha = 2.303A/t \qquad \textbf{Supplementary Equation 2}$$

The band energy of as-prepared hematite photoanodes can be estimated from the linear fit of $(\alpha hv)^{-n}$ vs. incident photon energy (hv) near the absorption edge. The determined indirect band energies were in line with the reported values.[3]

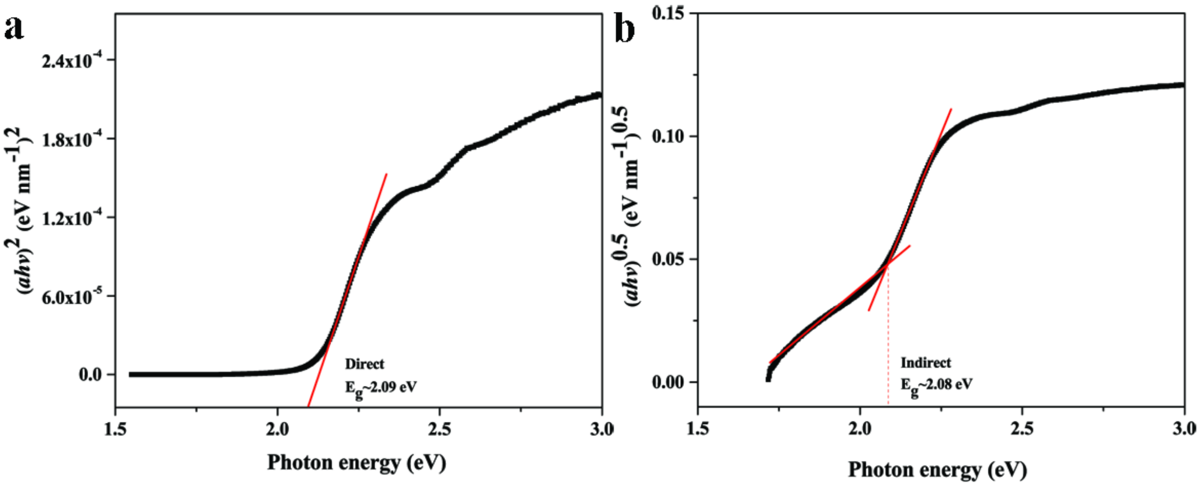

Supplementary Figure 3. Derived direct (a) and indirect (b) allowed transition band gap energies of an as-prepared hematite photoanode.

2. Two types of surface states

2.1 Basic settings and physical model selection for photo-electrochemical impedance spectroscopy (PEIS) analysis

Prior to each PEIS data acquisition, a time period of 1 min was given to establish steady-state PEC water oxidation conditions. Constant 5 mV AC perturbation with frequencies ranging from 0.1 to 10 kHz was employed. For quantitative analysis, a simplified physical model consisting of charge separation capacitance and charge transfer capacitance elements was applied. The data fitting was done with the built-in function of the Thales software.

Only two semicircles are observed in the experimental Nyquist plot (Supplementary Figure 5): the first semicircle appearing in the high frequency domain is assigned to the capacitance of the space charge layer, and the second semicircle in the low frequency domain is attributed to the capacitance of both surface states for water oxidation.[4,5] Therefore, an equivalent circuit with charge transfer capacitance and space charge layer capacitance is proposed to quantify their respective magnitude. In this case, the derived charge transfer capacitance is the overall value contributed by both surface states, and it is not possible to distinguish the individual charge transfer processes via ***S1*** and ***S2***.

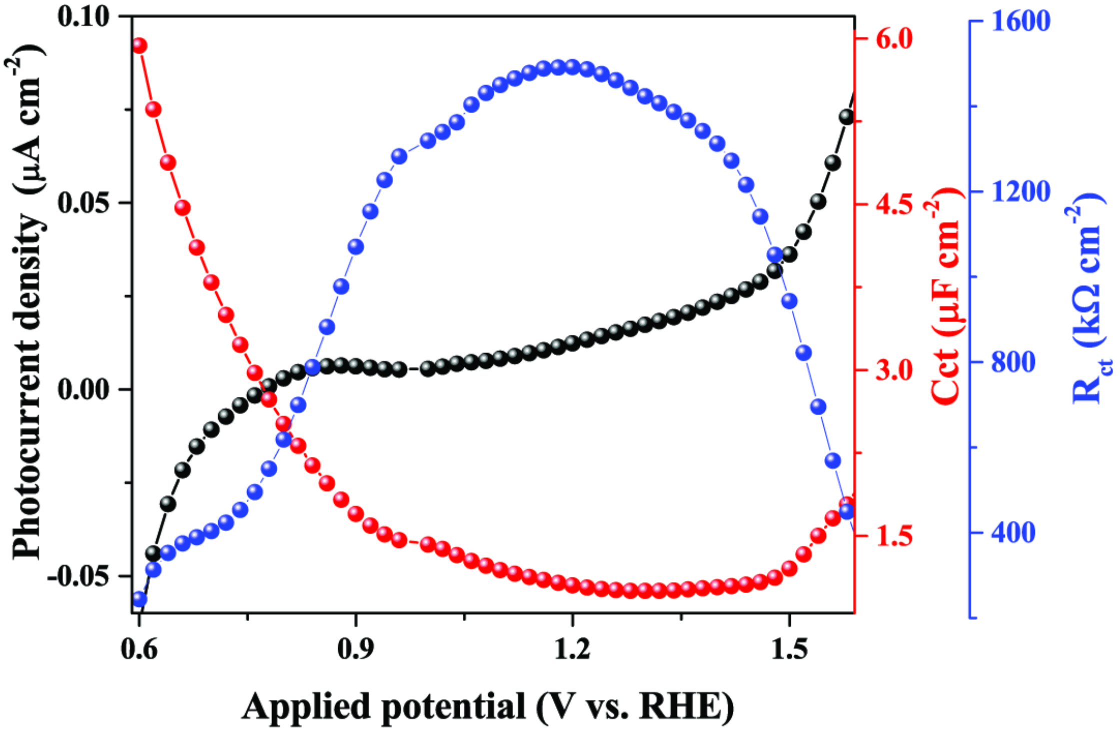

Supplementary Figure 4. Evolution of *J-V* (black spheres), surface state capacitance (red spheres) and charge transfer resistance (blue spheres) as a function of the applied potential (dark, pH 8.0).

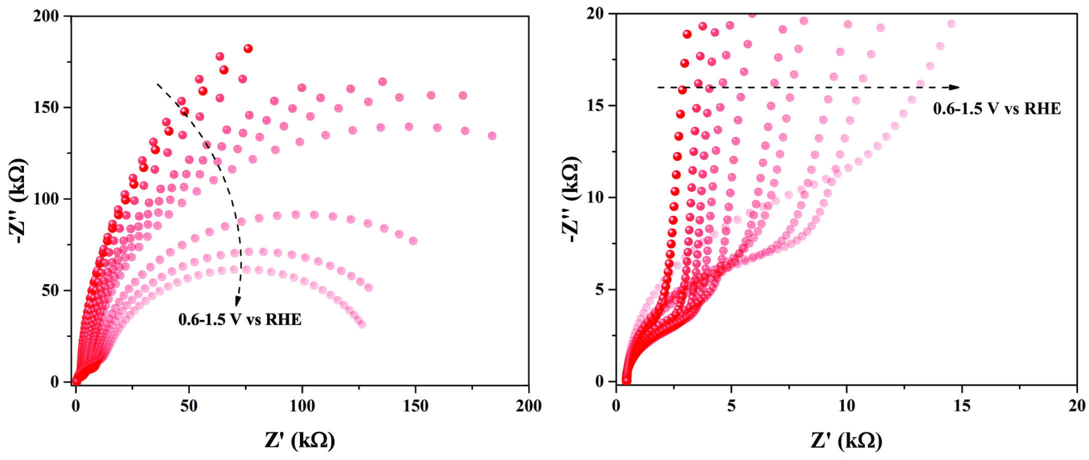

Supplementary Figure 5. Nyquist plots (left) and enlarged high frequency section (right) for hematite photoanodes measured at different applied potentials (0.6-1.5 V vs RHE) under 100 mW cm^{-2} illumination (pH 8.0).

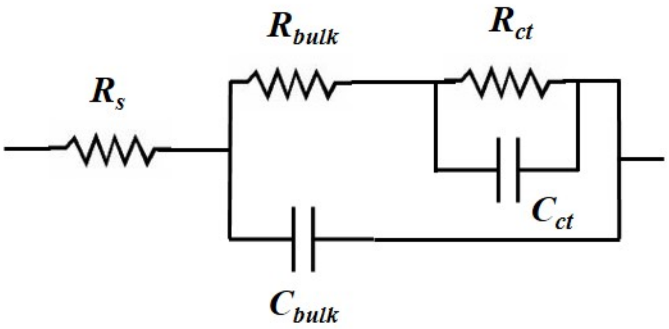

Supplementary Figure 6. Equivalent circuit used to simulate measured EIS data, C_{bulk} and C_{ct} are capacitances for bulk and surface states, respectively; R_{ct}, R_{bulk} and R_s are resistances related to surface charge transfer, bulk hematite and electrolyte, respectively.

2.2 Illumination intensity modulated PEIS and rate law analysis

To understand the effect of surface hole density on the reaction pathway, rate law analysis was done by modulating the illumination intensity (5-100 mW cm^{-2}) during PEIS measurements, as the water oxidation rate can be estimated from the steady-state photocurrent density (J_{ss}), and the surface hole density can be estimated from the following Equation:[6]

$$[h^+] = \left[C_{ct} * V_{applied} * \left(\frac{R_{ct}}{R_{ct}+R_{bulk}+R_s}\right)\right] \Big/ S \qquad \textbf{Supplementary Equation 3}$$

where $[h^+]$ is the surface hole density, $V_{applied}$ is the applied potential of the potentiostat during PEIS measurements (the potential applied between working electrode and reference electrode), S is the effective surface area.

Correspondingly, the reaction order of surface holes can be derived from Equation 4:[7]

$$J_{ss} = k_{app} * [h^+]^n \qquad \textbf{Supplementary Equation 4}$$

k_{app} is the apparent reaction rate constant associated with the water oxidation reaction, and n is the reaction order with respect to surface holes. The rate law analysis is valid based on following facts: (i) the hematite photoanode is stable under experimental conditions here, meaning that there is no material degradation induced by self-oxidation;[8] (ii) the Faradaic efficiency for water oxidation is unity, suggesting that there are no competitive reactions;[9] (iii) water oxidation is proceeding exclusively through surface states.[10]

The donor density (N_d) and flat band potential (E_{fb}) of photoanodes was determined using the Mott-Schottky equation:[11]

$$\left(\frac{A}{C_{bulk}}\right)^2 = \frac{2}{q\kappa\varepsilon_0 N_D}\left(V - E_{fb} - \frac{k_B T}{q}\right) \quad \textbf{Supplementary Equation 5}$$

where A is the surface area of the tested photoanode, C_{bulk} is the space charge capacitance, q is the elementary charge, κ is the dielectric constant of hematite (32 was used here), ε_0 is the vacuum permittivity, V is the applied DC potential, k_B is Boltzmann constant, and T is the absolute temperature.

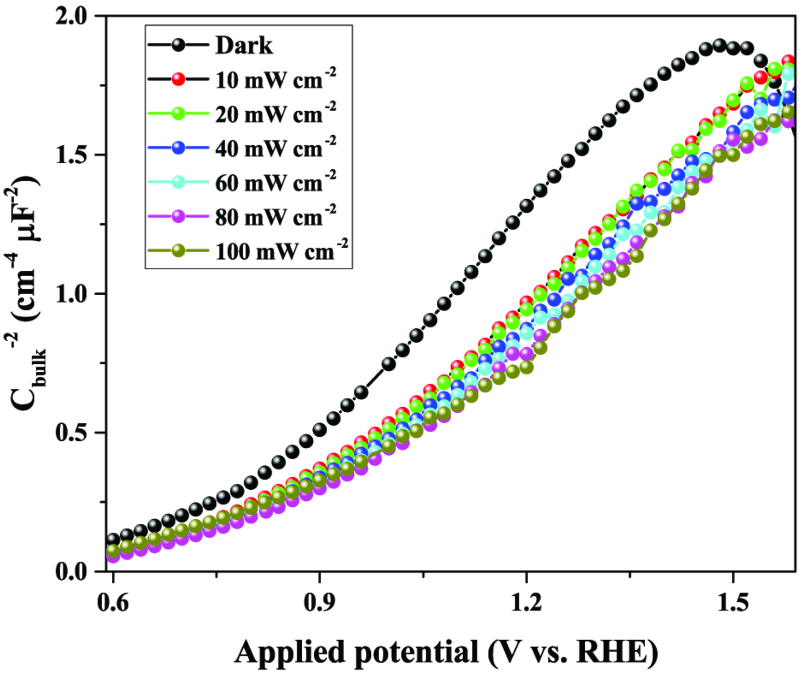

Supplementary Figure 7. Mott-Schottky plots for hematite photoanodes measured under different illumination intensities (0-100 mW cm^{-2}).

Supplementary Table 1. Flat band potential (E_{fb}) and donor density (N_d) of hematite photoanodes at different illumination intensities (0-100 mW cm^{-2})

Illumination intensity (mW cm^{-2})	E_{fb} (V vs. RHE)	N_d (cm^{-3})
0	0.72	1.65 x 10^{18}
10	0.78	1.86 x 10^{18}
20	0.80	1.85 x 10^{18}
40	0.80	1.94 x 10^{18}
60	0.81	1.98 x 10^{18}
80	0.82	2.00 x 10^{18}
100	0.82	2.01 x 10^{18}

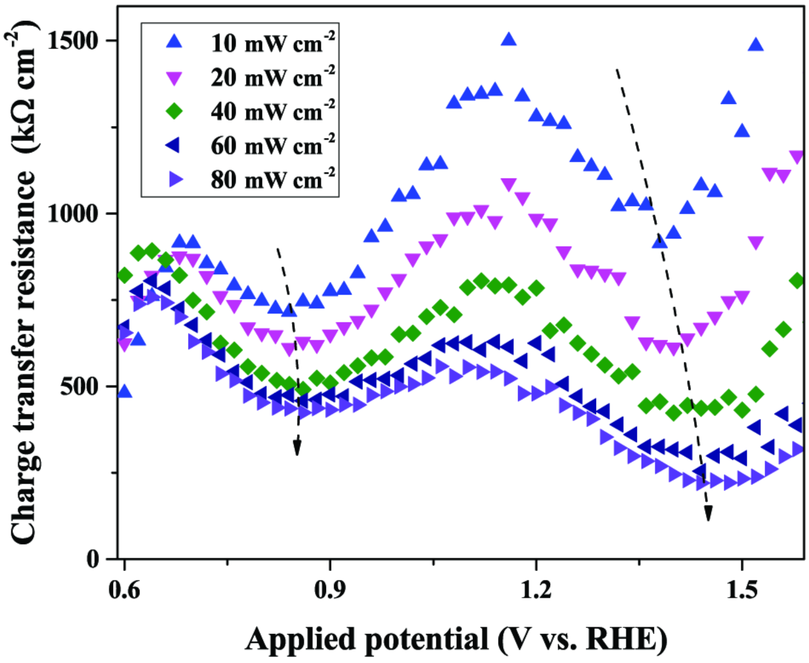

Supplementary Figure 8. Charge transfer resistance for hematite photoanodes measured under different illumination intensities.

2.3 Tracing surface states with fast cathodic CV scans

The presence of both surface states (referred to as ***S1*** and ***S2*** at high and low potentials, respectively) during PEIS measurements is attributed to the oxidation of different chemical species on the hematite surface.[12] In principle, both charged chemical species should again have well-defined reduction bands if the cathodic CV scan is capable of capturing them. Therefore, we first aged the photoanode at high potential

and illumination conditions for 1 min to warrant steady-state conditions for both surface states. Then the illumination was stopped and a fast cathodic CV scan (3 cycles at a speed of 200 mV s^{-1}) was conducted. Immediately after the illumination was switched off, a strong cathodic spike appeared which was attributed to the discharge (or recombination) of highly energetic surface species (possibly ***S1*** and valence band holes). This assignment is supported by the absence of a reduction band at around 1.3 V vs RHE for ***S1***. In contrast, a prominent reduction band set in around 1.1 V vs RHE with a maximum around 0.8 V vs RHE in the first CV cycle, which correlates well with the energetics of ***S2***. In the following CV cycles, the reduction of ***S2*** disappeared, indicating that ***S2*** is a reaction intermediate participating in PEC water oxidation. In order to understand the evolution dynamics of ***S2***, the above-mentioned fast cathodic CV measurements were conducted over a scan rate range from 20-5000 mV s^{-1}. Surprisingly, the capacitance of ***S2*** increases over time, which means that additional ***S2*** is formed after the illumination was stopped. We assigned this unexpected observation to the partial conversion of ***S1*** to ***S2***. Lastly, similar experiments were performed while either the initial illumination intensity or the starting potential was modulated. The ***S2*** population was found to be proportional to both illumination intensity and starting potential. This could be explained with two facts, namely: (i) the strong dependence of primary-generated surface holes (***S1***) on illumination intensity and potential; (ii) the transformation of the primary surface holes to the secondary surface states (***S2***) upon accumulation.[13]

2.4 Estimation of the lifetime of surface states

We designed the following experiment to estimate the lifetime of ***S2***: initially the photoanode was preconditioned (60 s) at high potential and illumination for steady-state conditions of both surface states; once the illumination is off, fast recombination of highly energetic ***S1*** and valence band holes give rise to a cathodic spike while the less energetic ***S2*** can last longer. After different time intervals in the dark, a fast cathodic CV scan followed in order to quantify the amount of resting ***S2***. No obvious change of the CVs could be identified, suggesting that ***S2*** did not undergo recombination and has a lifetime longer than 3 min.[14]

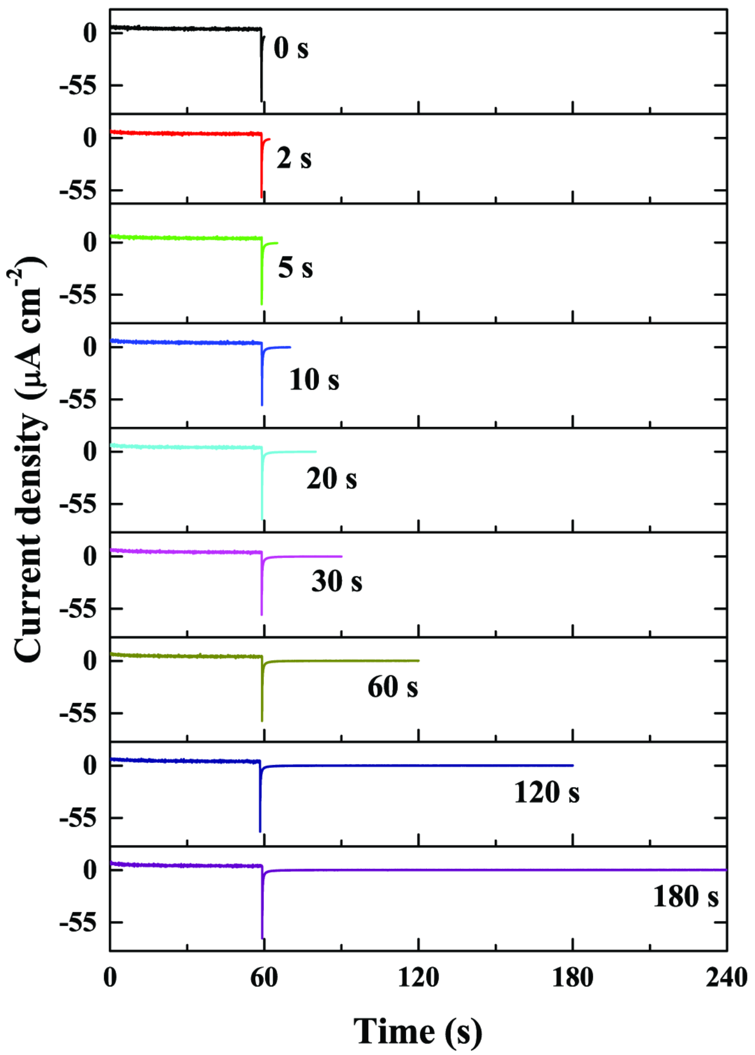

Supplementary Figure 9. Transient photocurrent operations of hematite photoanode: after achieving steady-state under illumination (100 mW cm^{-2}, 1 min), the light was switched off and different waiting times were observed before the initiation of a fast cathodic CV scan (Figure 2e).

2.5 Remarks on the lifetime of *S1* and *S2*

While the lifetime of ***S2*** was determined to be longer than 180 s according to the experiment in section **3.4**, the lifetime of ***S1*** is too short to probe it using the same approach. Therefore, we describe ***S2*** and ***S1*** to be long-lived and short-lived, respectively, in the present study. This assignment matches very well with their chemical identity (iron-oxo and iron-peroxo species, respectively) derived from rate law analysis. However, we notice that the terminology of describing the lifetime of intermediate species in previous publications is different and may sometimes be difficult for readers to follow. Therefore, we herein want to clarify this

issue. First, we found out that the long-lived photogenerated holes (~ 3 s) monitored by photoinduced absorption spectroscopy (~ 600 nm) were assigned to high-valent iron species (Fe^{IV}=O),[15–20] which is ***S1*** in our case. The lifetime of these photogenerated holes depends significantly on the applied potential, and it is actually very short when compared to ***S2*** in this study. Second, a long-lived peroxo intermediate (decay half time up to 90 s) was isolated before in a homogeneous iron-based catalyst.[21] In addition, a peroxo intermediate with lifetime exceeding 100 s was observed in some other heterogeneous systems.[22] In both cases, the lifetime of peroxo intermediates is in line with the one of ***S2*** that we probed here. Third, the Co^{IV}=O (lifetime in ms range) and superoxide (lifetime longer than 6 s) intermediate species determined by time-resolved Fourier-transform infrared spectroscopy on particular Co_3O_4 surfaces generally agree well with our assignments of ***S1*** and ***S2***.[14,23] In summary, unified trends could be derived from the above discussions with ***S2*** having a much longer lifetime than ***S1***. Therefore, we refer to them as long-lived and short-lived, respectively.

3. Distribution of both surface states in different electrolyte pH

3.1 PEIS analysis

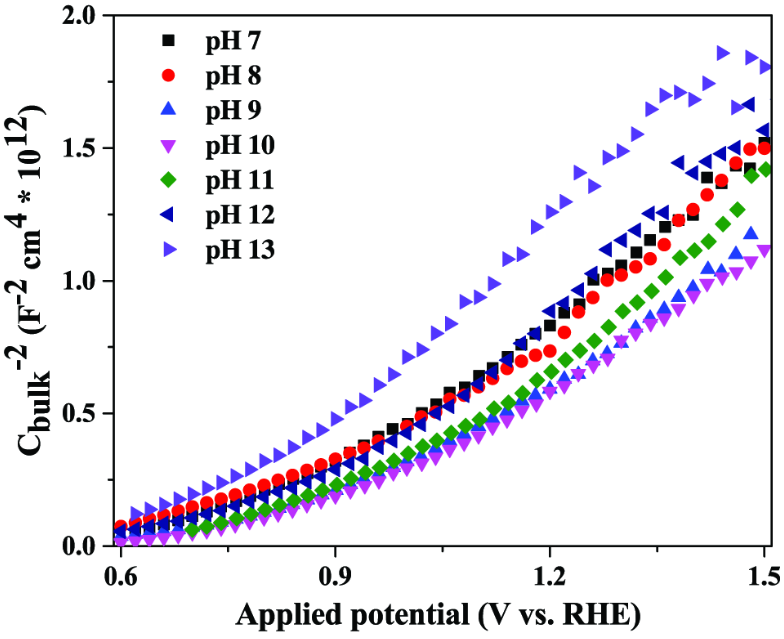

Supplementary Figure 10. Mott-Schottky plots for hematite photoanodes measured at different pH values (illumination intensity: 100 mW cm^{-2}).

Supplementary Table 2. Flat band potential (E_{fb}) and donor density (N_d) of a hematite photoanode at different electrolyte pH values (illumination intensity: 100 mW cm^{-2}).

Electrolyte pH	E_{fb} (V vs. RHE)	N_d (cm^{-3})
7	0.80	2.11 x 10^{18}
8	0.82	2.02 x 10^{18}
9	0.84	2.57 x 10^{18}
10	0.84	2.63 x 10^{18}
11	0.88	2.06 x 10^{18}
12	0.85	1.75 x 10^{18}
13	0.74	1.65 x 10^{18}

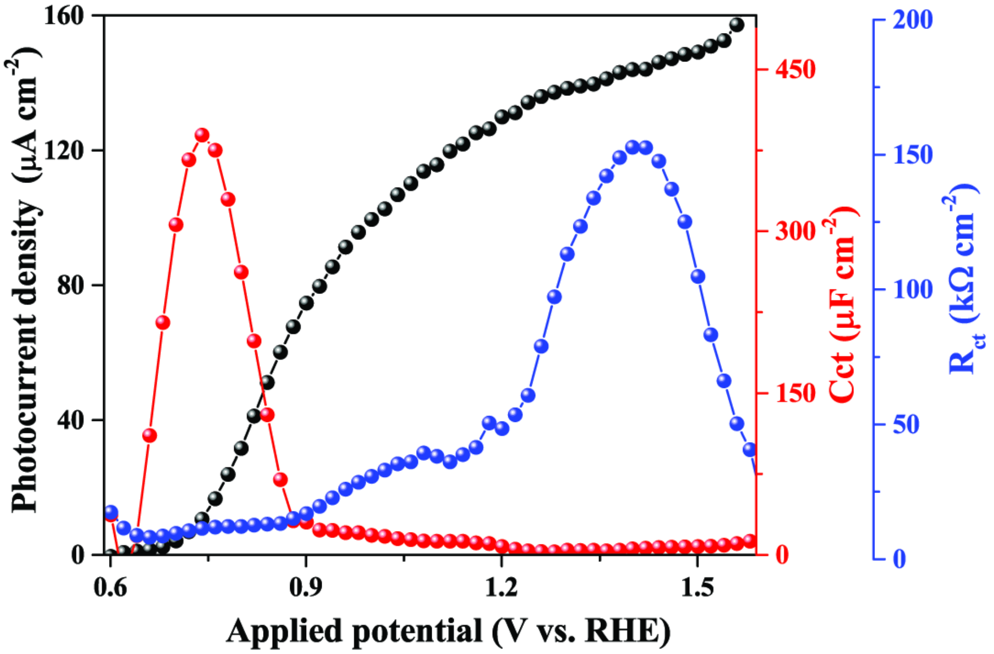

Supplementary Figure 11. Evolution of *J-V* (black spheres), surface state capacitance (red spheres) and charge transfer resistance (blue spheres) as a function of applied potential (illumination intensity: 100 mW cm^{-2}, 1 M NaOH).

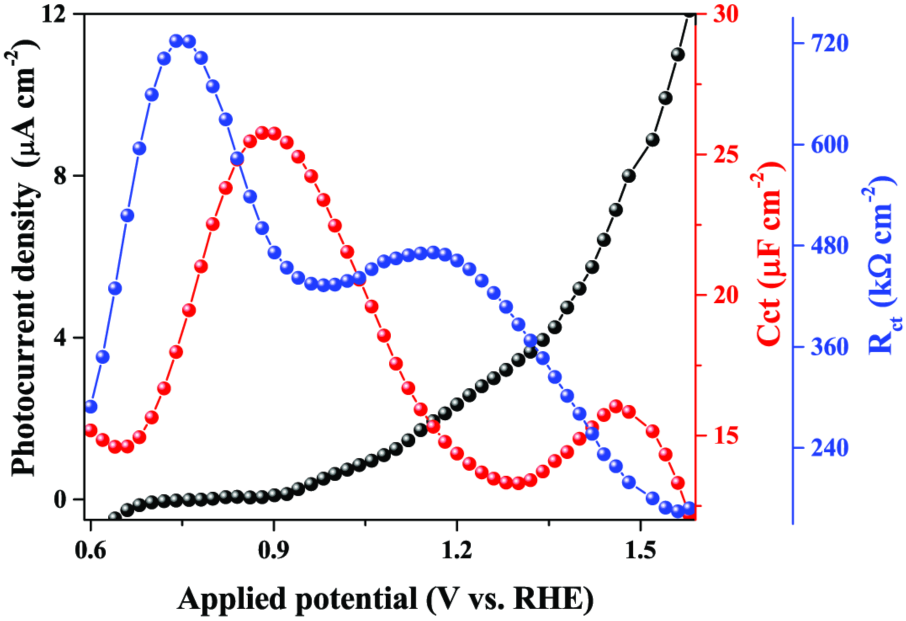

Supplementary Figure 12. Evolution of *J-V* (black spheres), surface state capacitance (red spheres) and charge transfer resistance (blue spheres) as a function of applied potential (illumination intensity: 100 mW cm^{-2}, pH 9.0).

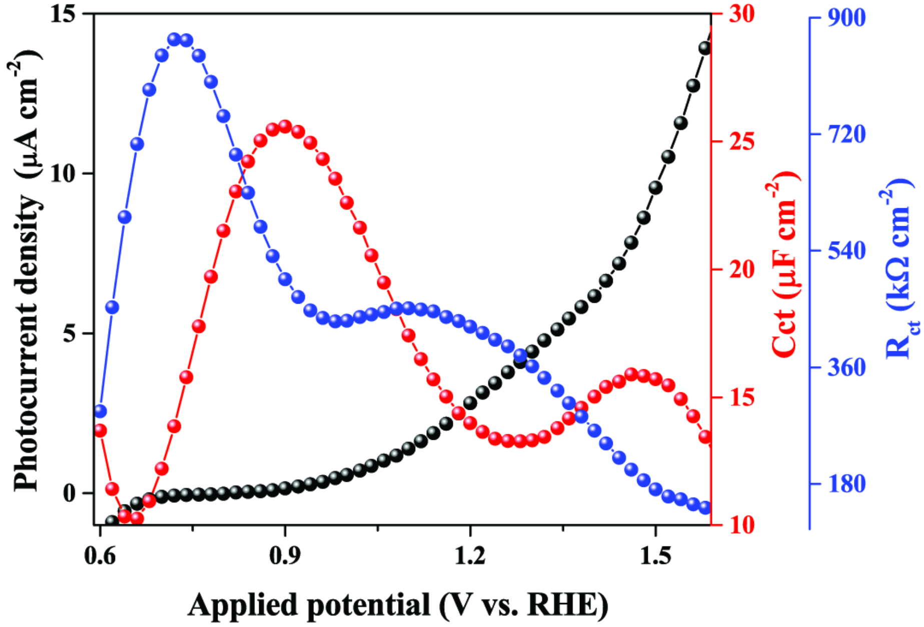

Supplementary Figure 13. Evolution of *J-V* (black spheres), surface state capacitance (red spheres) and charge transfer resistance (blue spheres) as a function of applied potential (illumination intensity: 100 mW cm^{-2}, pH 10.0).

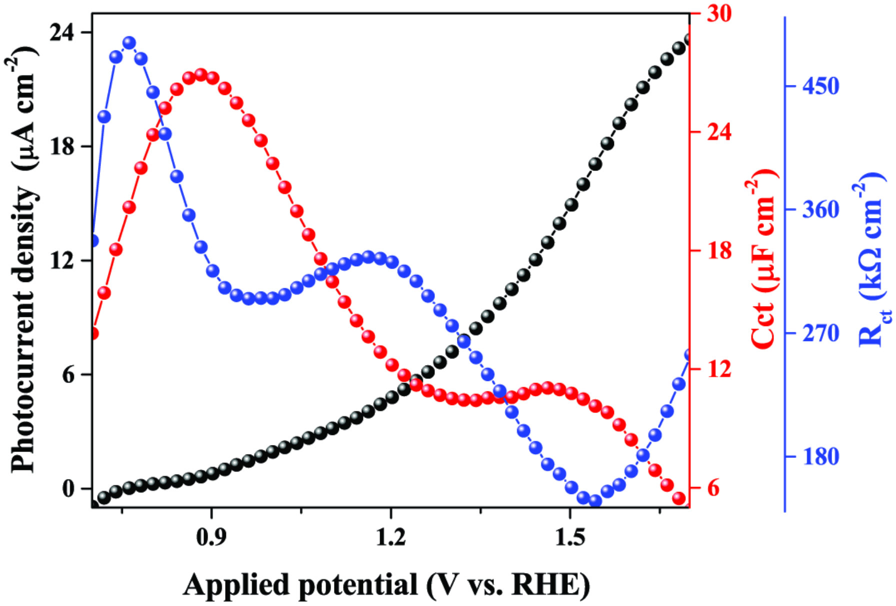

Supplementary Figure 14. Evolution of *J-V* (black spheres), surface state capacitance (red spheres) and charge transfer resistance (blue spheres) as a function of applied potential (illumination intensity: 100 mW cm^{-2}, pH 11.0).

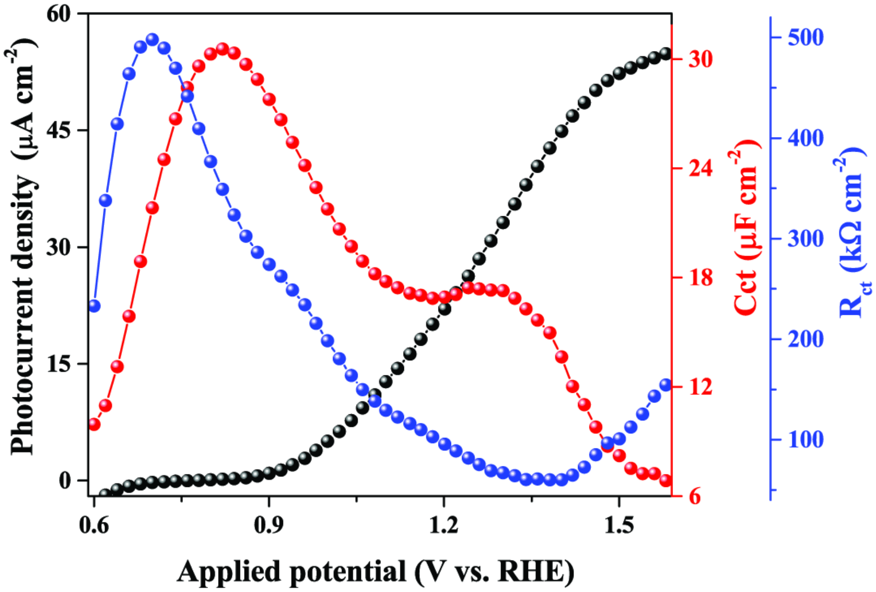

Supplementary Figure 15. Evolution of *J-V* (black spheres), surface state capacitance (red spheres) and charge transfer resistance (blue spheres) as a function of applied potential (illumination intensity: 100 mW cm^{-2}, pH 12.0).

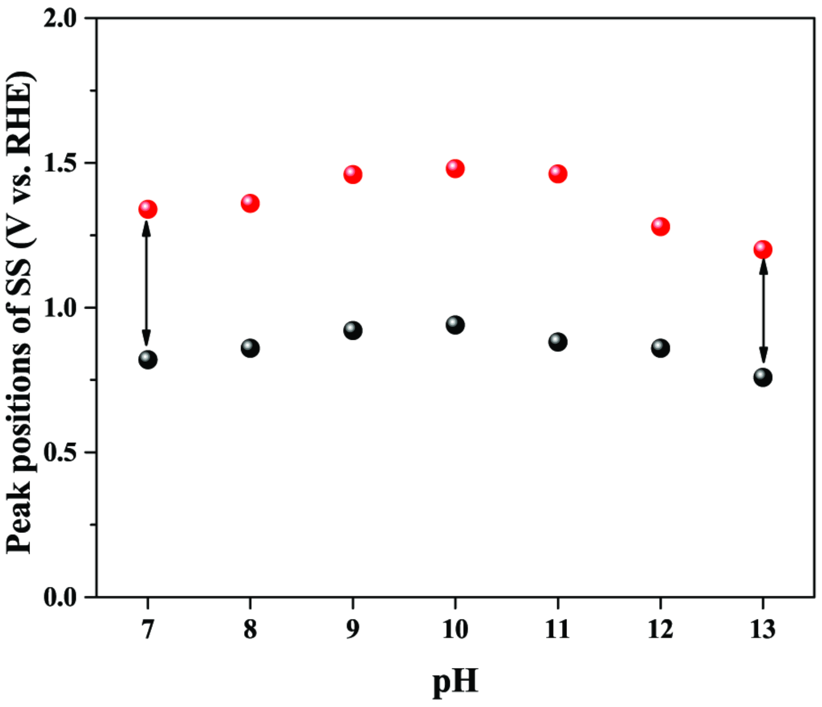

Supplementary Figure 16. Peak positions of both surface states at different electrolyte pH.

3.2 Capturing surface states using fast cathodic CV scans at pH 13

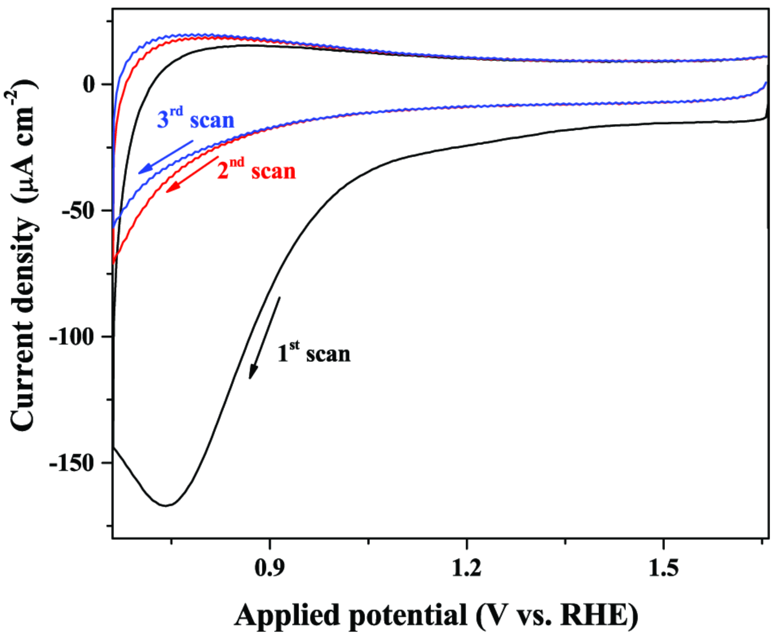

Supplementary Figure 17. Fast cathodic CV scans (200 mV s^{-1}) of a photoanode at pH 13 after preconditioning at steady-state photoelectrochemical OER (100 mW cm^{-2}) at high potential for 1 min, different scan cycles are labeled for comparison.

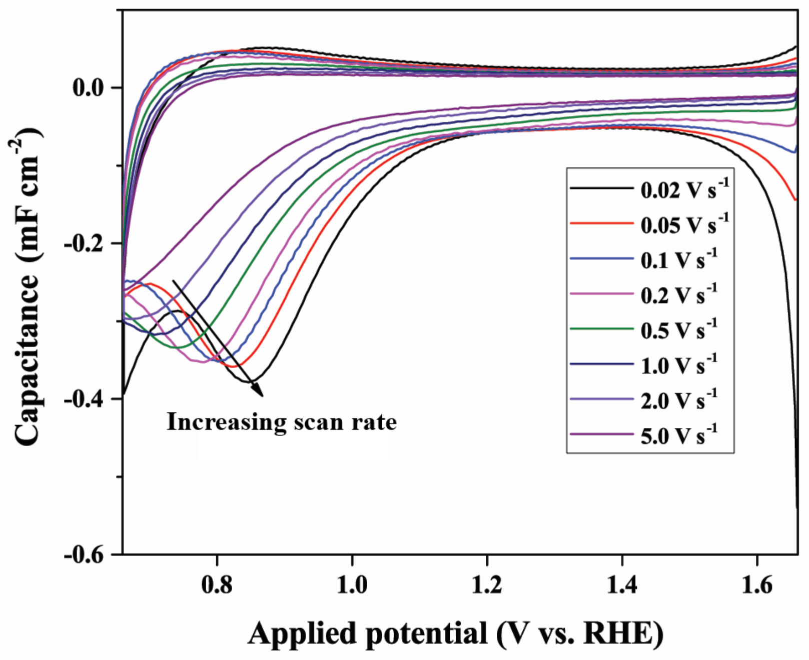

Supplementary Figure 18. Capacitive plot of the first CV scans measured at the same conditions as in Supplementary Figure 17 except for different scan rates; the capacitance decrease at increasing scan rate is labeled for clarity.

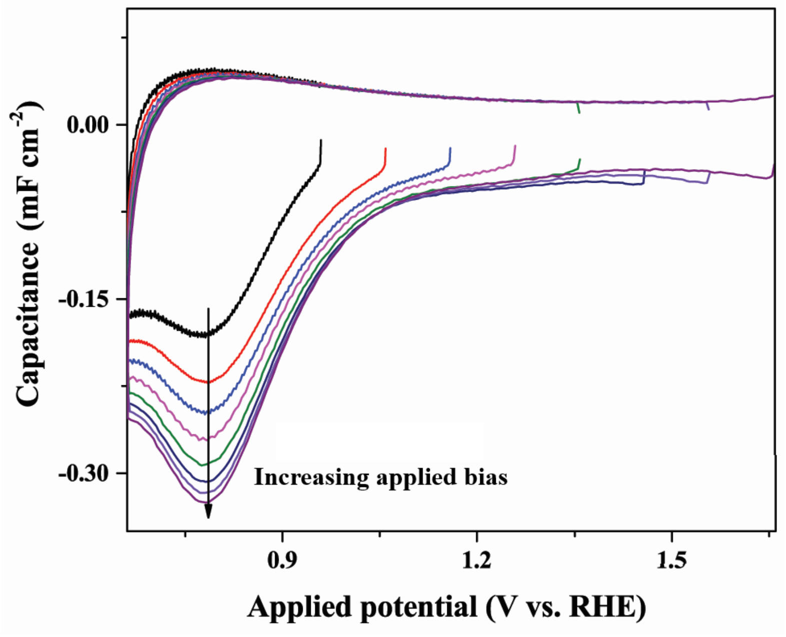

Supplementary Figure 19. Fast cathodic CV scans (200 mV s^{-1}) of a photoanode at pH 13 after preconditioning at different potentials for 1 min (illumination intensity: 100 mW cm^{-2}).

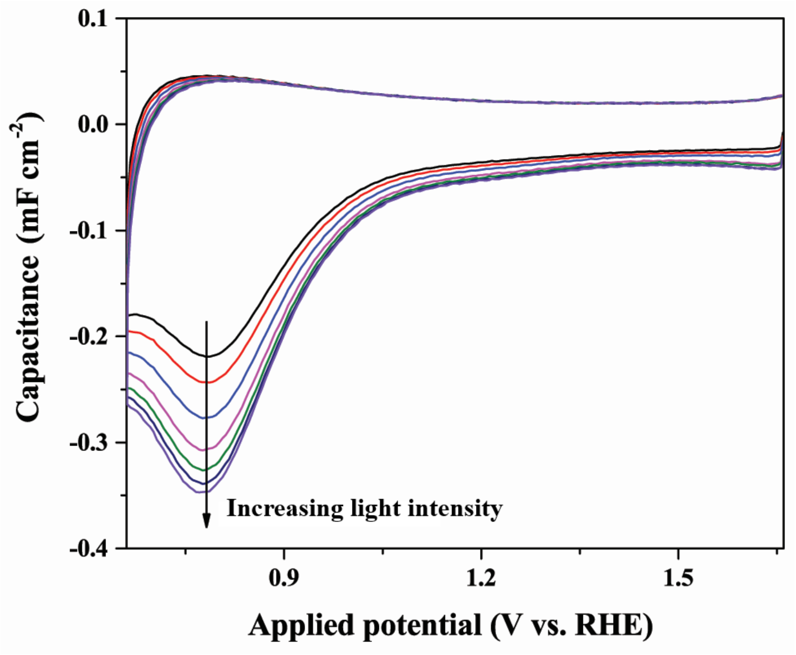

Supplementary Figure 20. Fast cathodic CV scans (200 mV s^{-1}) of a photoanode at pH 13 after preconditioning at different illumination intensities (5-100 mW cm^{-2}) at high potential for 1 min.

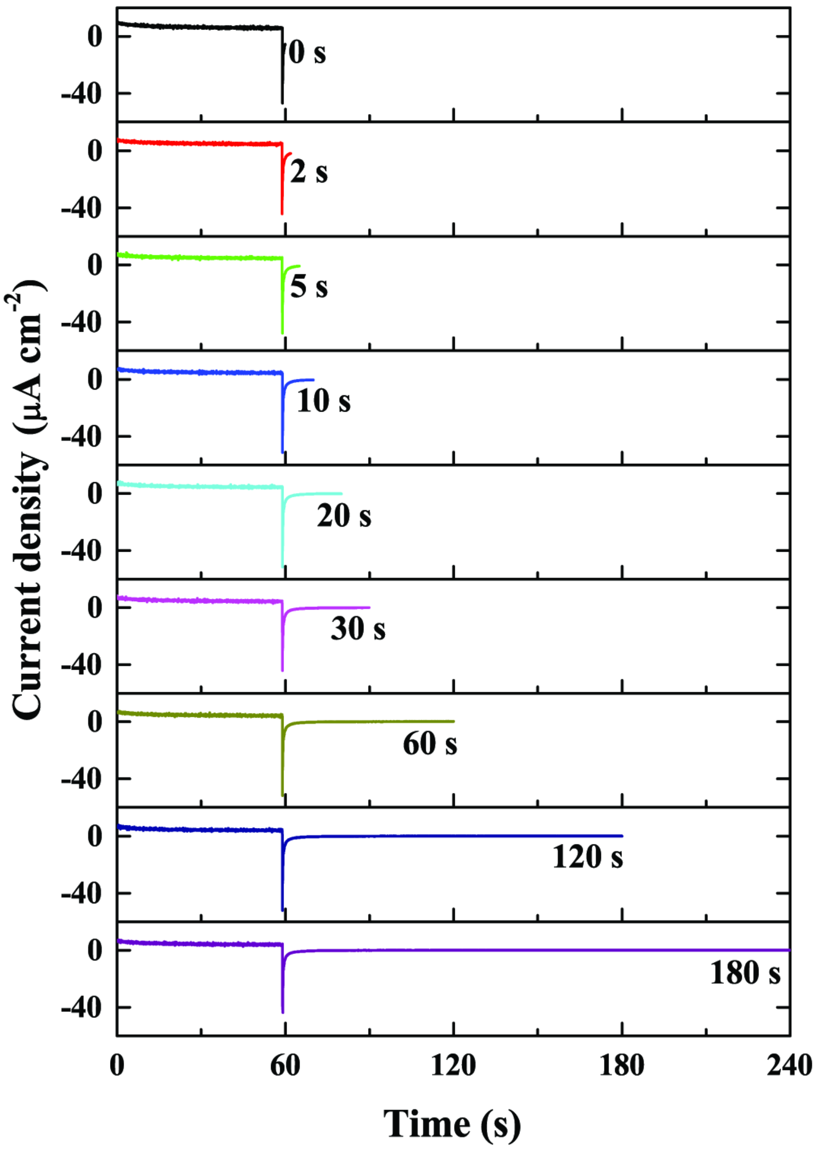

Supplementary Figure 21. Transient photocurrent operations of hematite photoanode: after achieving steady-state illumination conditions (100 mW cm^{-2}, 1 min), the light was switched off and different waiting intervals were observed before initiation of a fast cathodic CV scan.

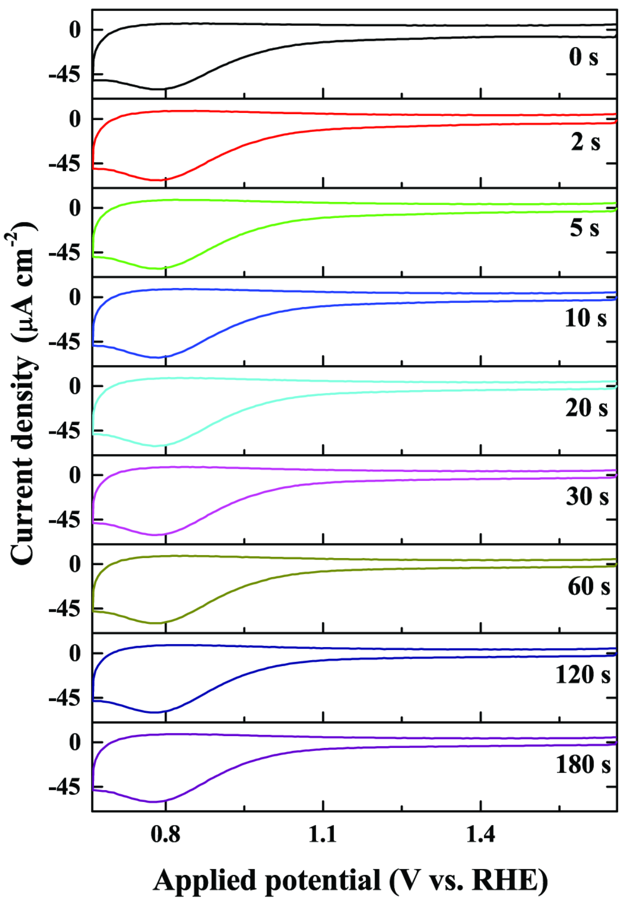

Supplementary Figure 22. Fast cathodic CV scans (200 mV s^{-1}) of a photoanode at pH 13 after the transient operation in Supplementary Figure 21 (different waiting intervals in the dark).

3.3 Near steady-state CV analysis at different pH values

In order to determine the point of zero charge (PZC) of the hematite surface, slow CV scans (5 mV s^{-1}) were performed at different electrolyte pH under 100 mW cm^{-2} illumination. As shown in Figure 3c, distinct CV features could be identified in different pH regions: (i) from pH 7 to 10, the photocurrent densities are very low at 1.23 V vs. RHE (between 0.025 and 0.035 mA cm^{-2}), and onset potentials are exceptionally stable in this region. After the onset potentials, the current densities gradually increase while remaining comparable at higher potentials; (i) above pH 11, current densities increase rapidly in the region of lower potentials, meanwhile, obvious photocurrent plateaus appeared after the initial growth; and interestingly (ii) around pH 11, a distinct current density pseudo-peak appears around 1.0-1.1 V vs. RHE. Clearly the interfacial protonation/deprotonation process plays a significant role for the observed phenomena.[24,25] The pseudo-peak observed at pH 11 could be attributed to the transition of the surface protonation state. When there are sufficient hydroxyl species available in the diffusion region, the surface of hematite is

deprotonated and hydroxyl species act as the dominant hole acceptor. Meanwhile, the surface will transform to the protonated state if the diffusion of hydroxyl species is limited. In this case, water molecules become the dominant hole acceptor. The distinct evolution behavior under both low and high pH conditions generally supports this hypothesis. In principle, the protonation state change at the photoanode surface significantly alters the behavior of both surface states. Since the primary photo-generated ***S1*** is located at higher oxidative potentials, at steady-state, it has three consumption pathways: direct transfer to surface hydroxyls or water molecules (depending on the surface protonation state), transformation to less energetic ***S2***, and recombination with conduction band electrons. Obviously, the electrolyte pH can alter this steady-state equilibrium by changing the individual kinetic processes.[26,27] For example, at pH 13 the recombination process is suppressed and charge transfer through formation of ***S2*** is facilitated (3rd order reaction).

3.4 Transient photocurrent spectra as a function of illumination intensity

To investigate the effect of illumination intensity on the surface protonation state change, transient photocurrent spectra were recorded from 5-100 mW cm^{-2} at constant applied potential (1.4 V vs. RHE). The transition of the surface protonation state starts between 30-40 mW cm^{-2}, as indicated by the transient photocurrent profile (Supplementary Figure 23, pH 11) and the decay half time (Supplementary Table 3). The analysis of the steady-state photocurrent density in Supplementary Figure 24 further corroborates this assignment. To exclude the electrolyte concentration effect, TPS measurements were also conducted in 0.5 M $NaClO_4$ (pH 11) under illumination intensity modulation conditions (Supplementary Figure 25).

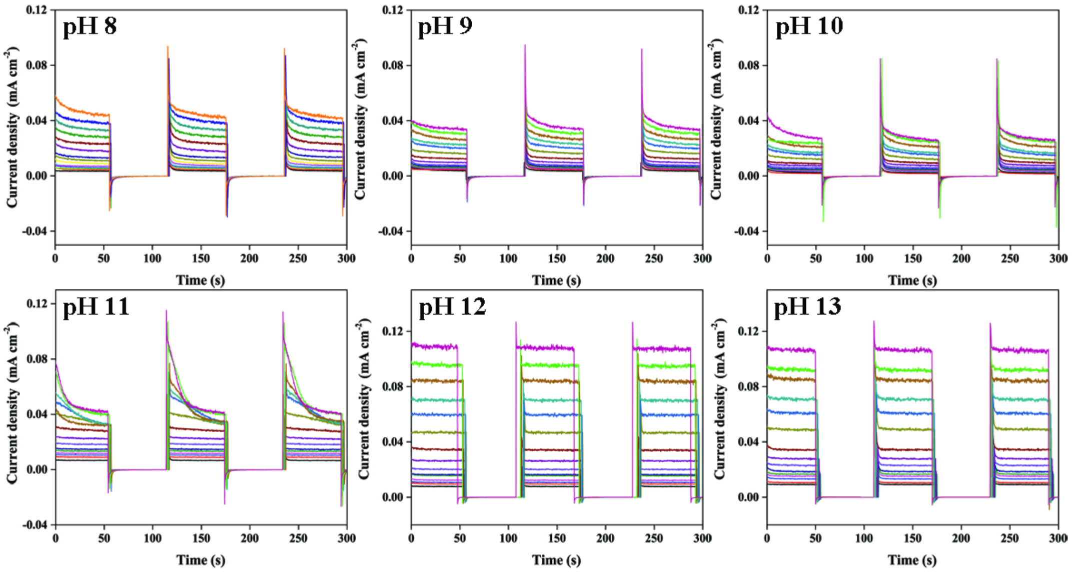

Supplementary Figure 23. Transient photocurrent profiles of hematite photoanode measured at different pH values (8-13) and different illumination intensities (5-100 mW cm^{-2}, see Supplementary Table 3).

Supplementary Table 3. Anodic decay half time of a hematite photoanode measured at different pH values (8-13) and different illumination intensities (5-100 mW cm^{-2}).

Light intensity (mW cm^{-2})	$t_{1/2}$ (s)					
	pH 8	pH 9	pH 10	pH 11	pH 12	pH 13
5	1.6	1.6	1.6	0.8	0.8	1.0
6	1.4	1.6	1.6	0.8	0.8	0.8
7	1.4	1.6	1.4	0.8	0.8	0.8
8	1.4	1.6	1.4	0.6	0.8	0.8
9	1.4	1.4	1.2	0.6	0.8	0.8
10	1.4	1.4	1.2	0.6	0.6	0.6
12	1.0	1.4	1.0	0.6	0.6	0.6
15	1.0	1.0	0.8	0.6	0.6	0.6
20	1.0	1.0	0.8	0.6	0.4	0.6
30	0.8	0.8	0.8	0.8	0.4	0.4
40	0.6	0.6	0.6	4.6	0.2	0.4
50	0.8	0.6	0.6	10.4	0.2	0.4
60	0.6	0.6	0.6	9.6	0.2	0.4
80	0.6	0.6	0.4	7.4	0.2	0.2
100	0.6	0.4	0.4	6.8	0.2	0.2

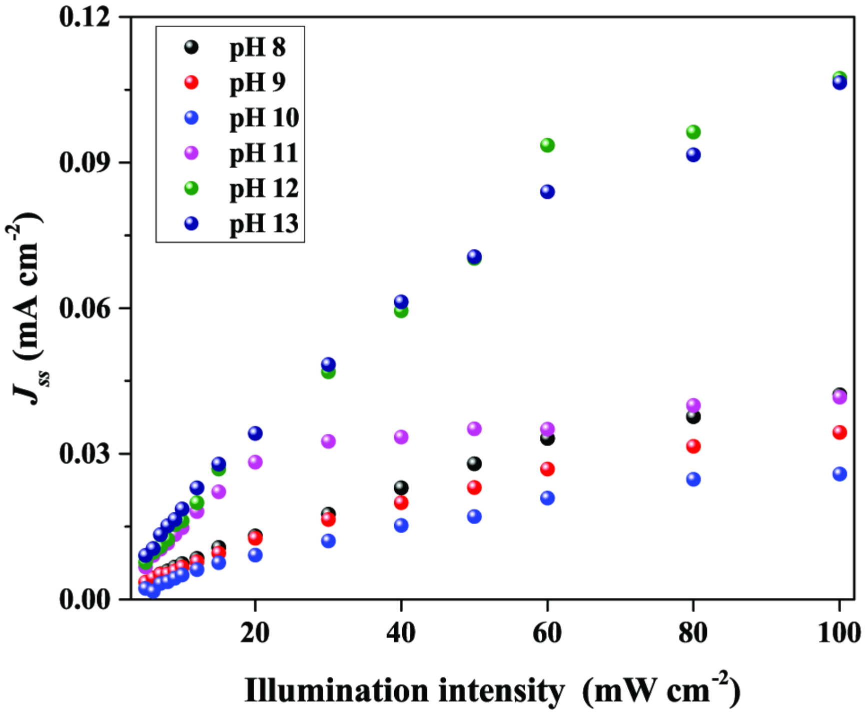

Supplementary Figure 24. Evolution of steady-state photocurrent (J_{ss}) of hematite photoanode measured at different pH (8-13) and different illumination intensities (5-100 mW cm^{-2}).

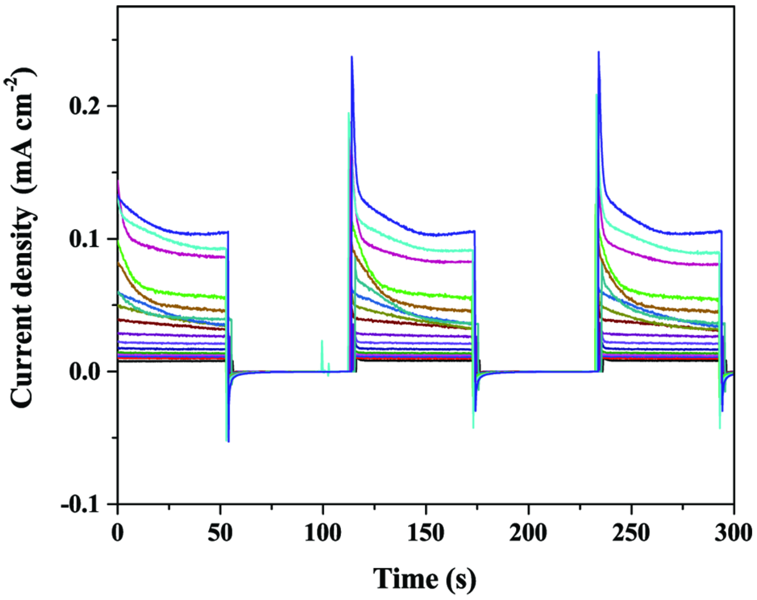

Supplementary Figure 25. Transient photocurrent profiles of a hematite photoanode measured at pH 8 (0.5 M $NaClO_4$) and different illumination intensities (5-100 mW cm^{-2}).

3.5 Transient photocurrent spectra as a function of applied potential

When the illumination intensity was kept constant (100 mW cm^{-2}) and the applied potential increased steadily from 0.9-1.5 V vs. RHE, the prominent slow decay feature of the anodic photocurrent density only occurs at around pH 11. The analysis of the anodic photocurrent decay half time ($t_{1/2}$) in Supplementary Table 4 indicates that the transition of surface protonation state starts between 1.0-1.1 V vs. RHE. Clearly, the photoanode surface is deprotonated when the applied potential is below this transition point, while above it, the surface becomes protonated. To visualize this protonation state change, the evolution of the steady-state photocurrent density is summarized in Supplementary Figure 27 as a function of applied potential and electrolyte pH, highlighting the importance of surface protonation state on the surface state property and thus on the catalytic activity difference.

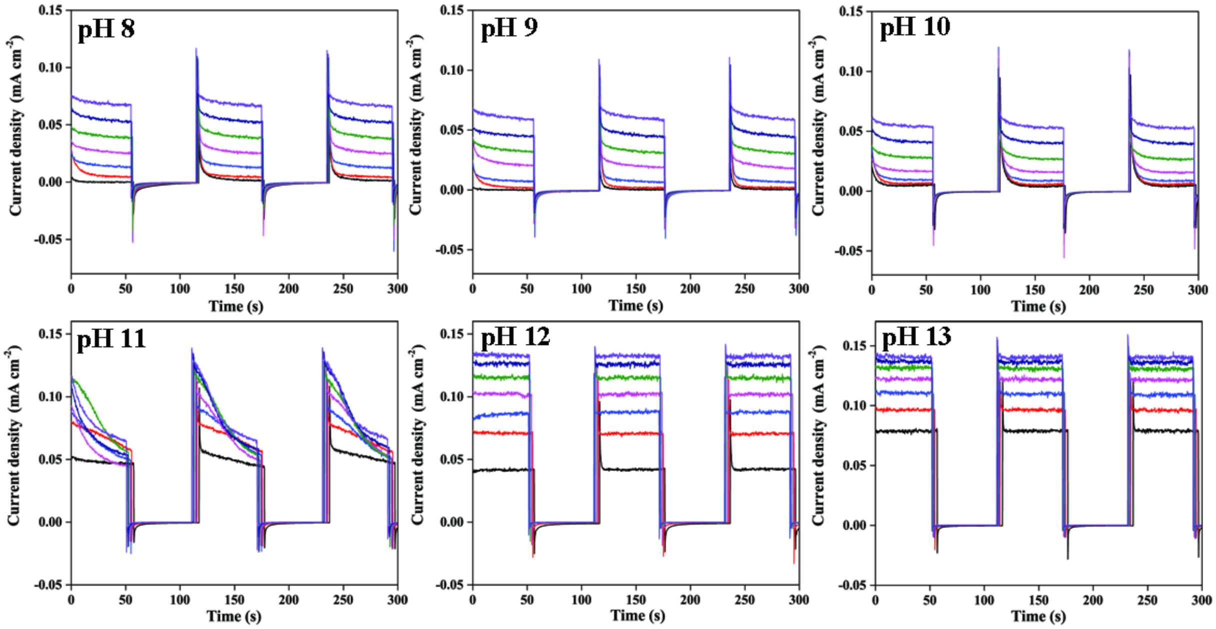

Supplementary Figure 26. Transient photocurrent profiles of hematite photoanode measured at different pH (8-13) and different applied potentials (0.9-1.5 V vs RHE, see Supplementary Table 4).

Supplementary Table 4. Anodic decay half time of a hematite photoanode measured at different pH (8-13) and different applied potentials (0.9-1.5 V vs RHE).

Potential (V vs. RHE)	$t_{1/2}$ (s)					
	pH 8	pH 9	pH 10	pH 11	pH 12	pH 13
0.9	0.4	0.6	0.8	0.2	0.2	0.2
1.0	0.6	0.6	0.8	0.6	0.2	0.4
1.1	0.8	0.6	1.0	7.6	0.2	0.4
1.2	0.8	0.8	0.8	15.2	0.2	0.2
1.3	0.6	0.6	0.8	20.6	0.4	0.4
1.4	0.6	0.4	0.6	16.8	0.4	0.4
1.5	0.4	0.8	0.6	16.4	0.4	0.4

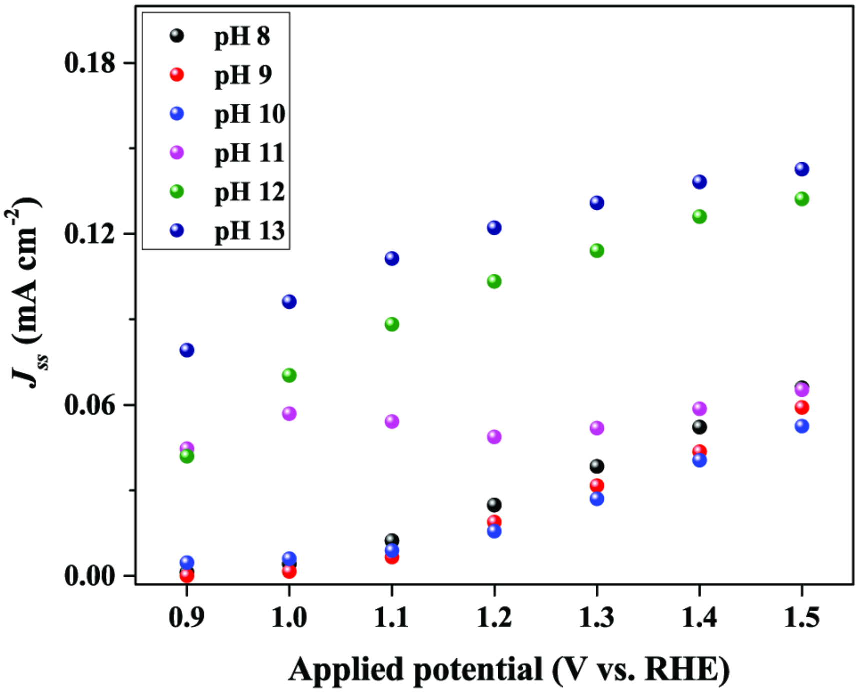

Supplementary Figure 27. Evolution of steady-state photocurrent (J_{ss}) of hematite photoanode measured at different pH (8-13) and different applied potentials (0.9-1.5 V vs RHE).

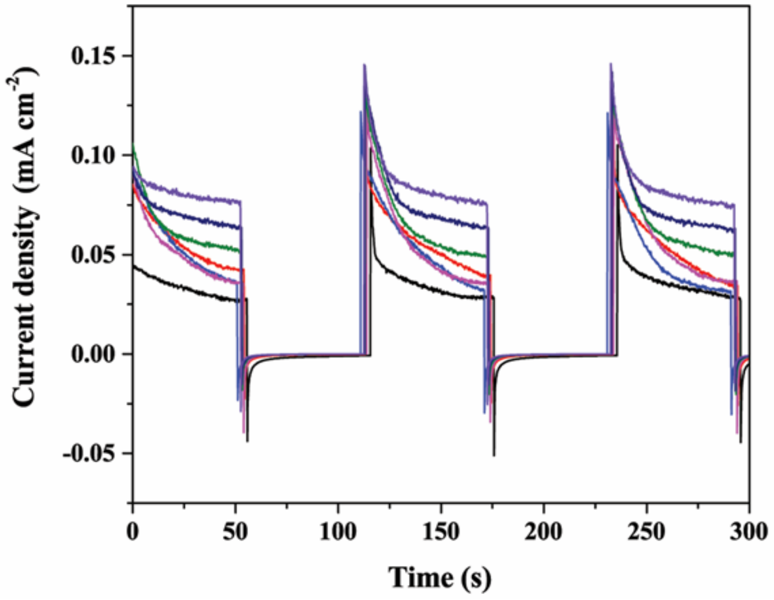

Supplementary Figure 28. Transient photocurrent profiles of a hematite photoanode measured at pH 8 (0.5 M $NaClO_4$) and different applied potentials (0.9-1.5 V vs RHE).

3.6 Hole transfer efficiency

Calculation of hole transfer efficiency from transient photocurrent spectroscopy measurements:[28]

$$\eta = \frac{J_\infty}{J_0} = \frac{k_{ct}}{k_{ct}+k_{rec}} \quad \textbf{Supplementary Equation 6}$$

where J_∞ is the steady-state photocurrent density, J_0 is the anodic spike, η is the hole transfer efficiency, k_{ct} and k_{rec} are the hole transfer rate constant and recombination rate constant, respectively.

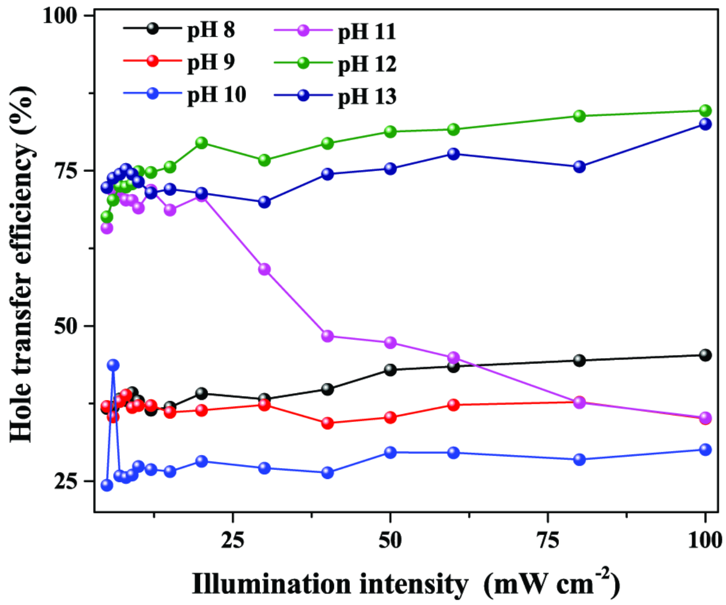

Supplementary Figure 29. Evolution of hole transfer efficiency of a hematite photoanode measured at different pH (8-13) and different illumination intensities (5-100 mW cm^{-2}).

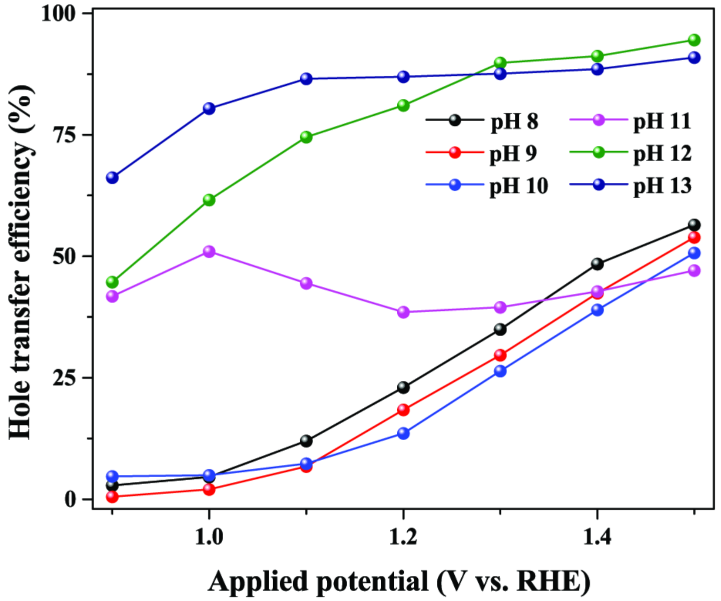

Supplementary Figure 30. Evolution of hole transfer efficiency of a hematite photoanode measured at different pH (8-13) and different applied potentials (0.9-1.5 V vs RHE).

3.7 Rate law analysis near the PZC

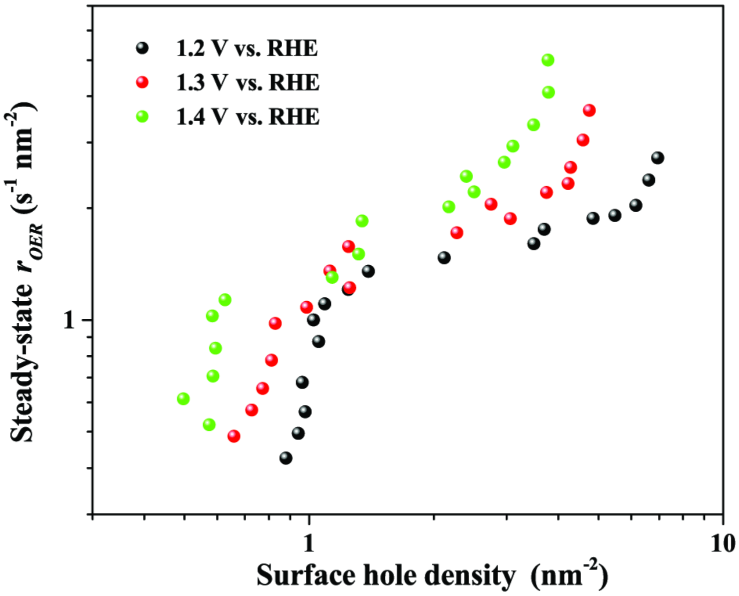

Supplementary Figure 31. Rate law analysis at different potentials (1.2-1.4 V vs. RHE, pH 11); steady-state OER rate (r_{OER}) and surface hole densities were probed by intensity modulated PEIS.

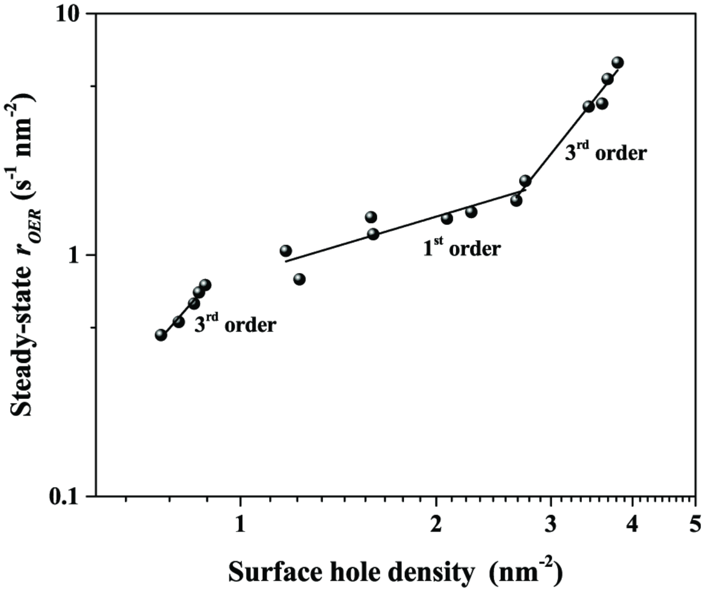

Supplementary Figure 32. Rate law analysis at 1.3 V vs. RHE (pH 11, 0.5 M $NaClO_4$) where ***S2*** reaches maximum values; steady-state OER rate (r_{OER}) and surface hole densities were probed by intensity modulated PEIS.

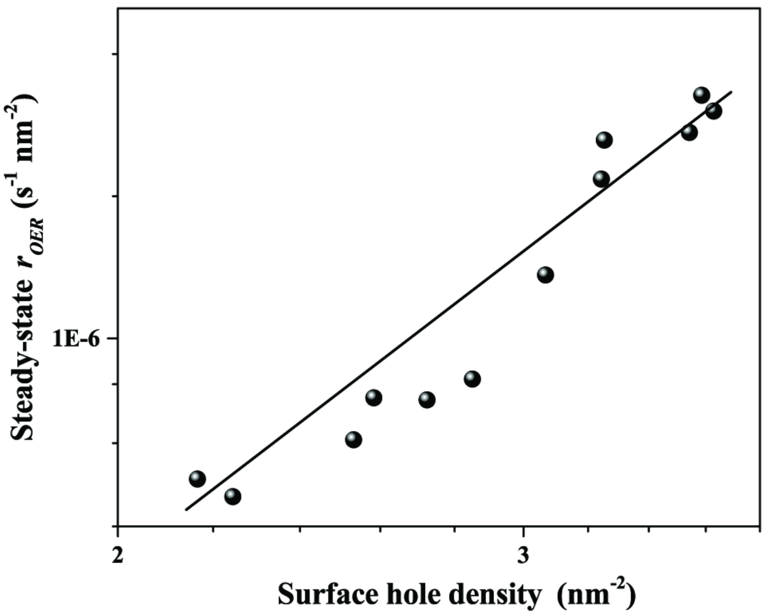

Supplementary Figure 33. Rate law analysis at 0.9 V vs RHE (pH 11) where ***S2*** reaches maximum values; steady-state OER rate (r_{OER}) and surface hole densities were probed by intensity modulated PEIS.

4. Distribution of both surface states in buffered electrolyte

4.1 PEIS analysis in buffered electrolyte

Buffered electrolyte (0.05 M borate buffer) was employed to verify the key influence of surface protonation states on the distribution and density of both surface states. The above-used PEIS measurements were repeated and simulated using a similar physical model. Clearly, the presence of ***S1*** was significantly retarded at pH 9 and almost invisible at pH 10.

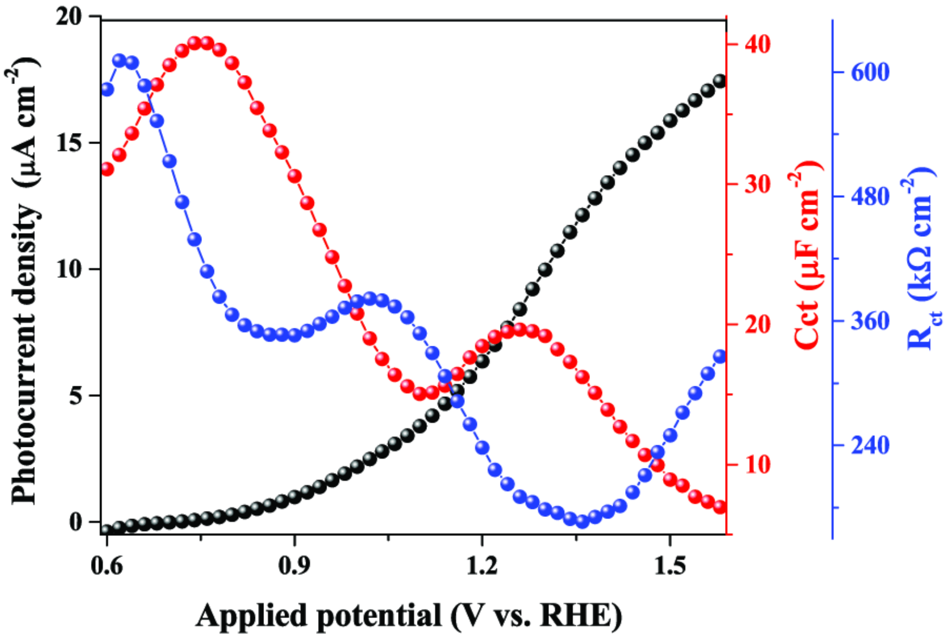

Supplementary Figure 34. Evolution of *J-V* (black spheres), surface state capacitance (red spheres) and charge transfer resistance (blue spheres) as a function of applied potential (illumination intensity: 100 mW cm^{-2}, pH 7.0).

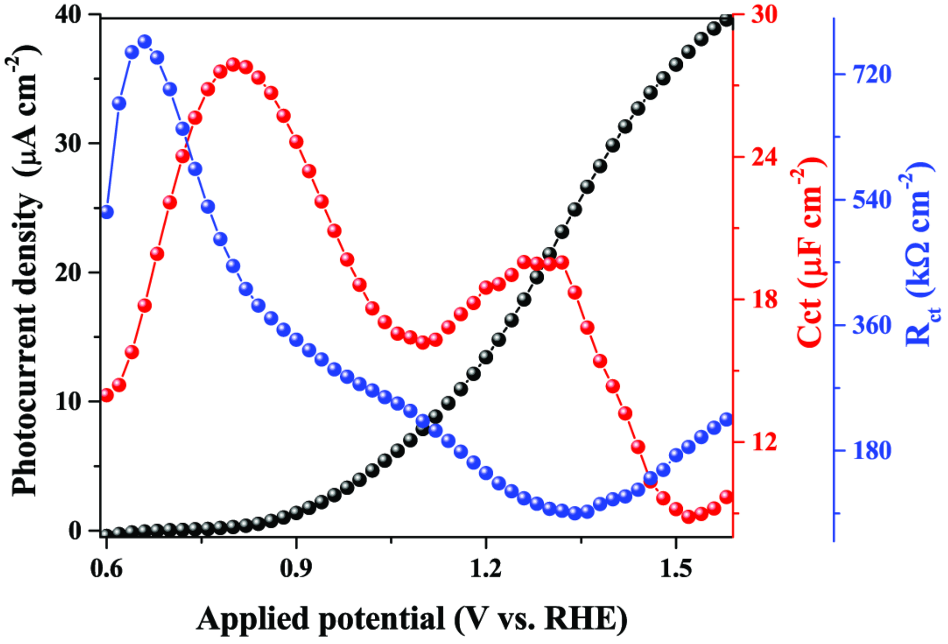

Supplementary Figure 35. Evolution of *J-V* (black spheres), surface state capacitance (red spheres) and charge transfer resistance (blue spheres) as a function of applied potential (illumination intensity: 100 mW cm^{-2}, pH 8.0).

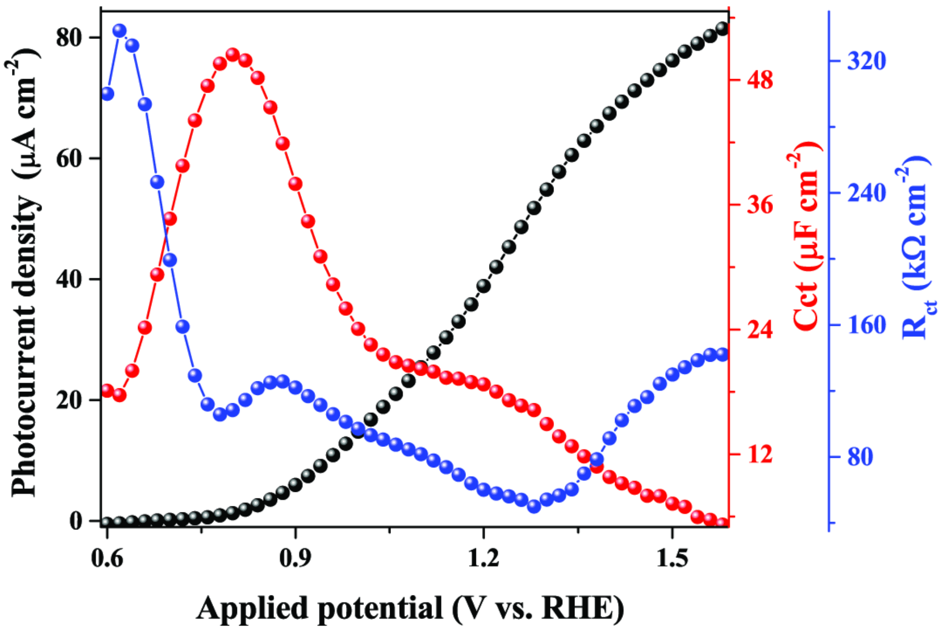

Supplementary Figure 36. Evolution of *J*-*V* (black spheres), surface state capacitance (red spheres) and charge transfer resistance (blue spheres) as a function of applied potential (illumination intensity: 100 mW cm^{-2}, pH 9.0).

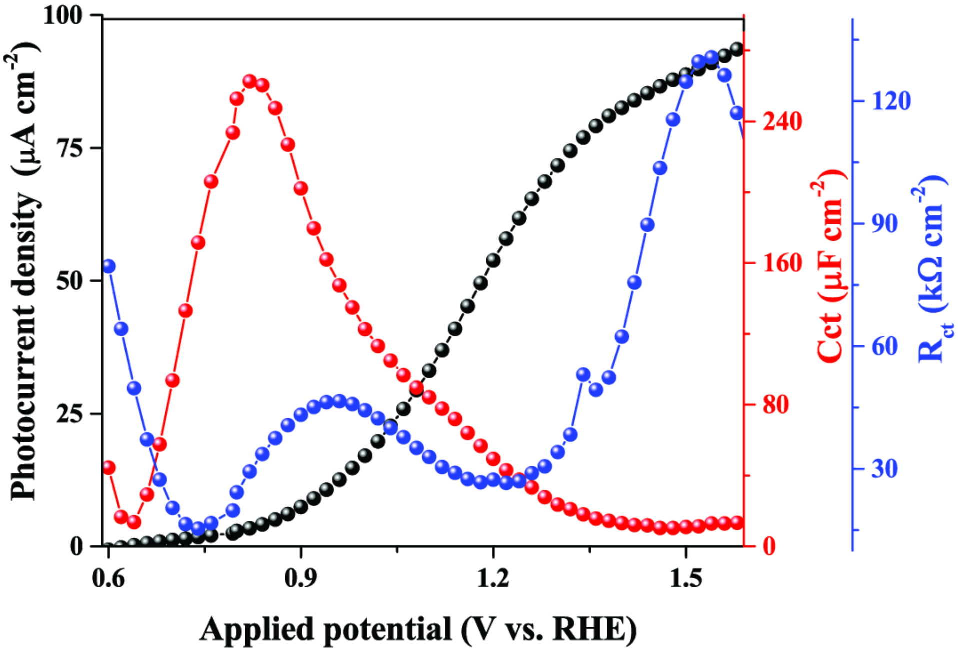

Supplementary Figure 37. Evolution of *J*-*V* (black spheres), surface state capacitance (red spheres) and charge transfer resistance (blue spheres) as a function of applied potential (illumination intensity: 100 mW cm^{-2}, pH 10.0).

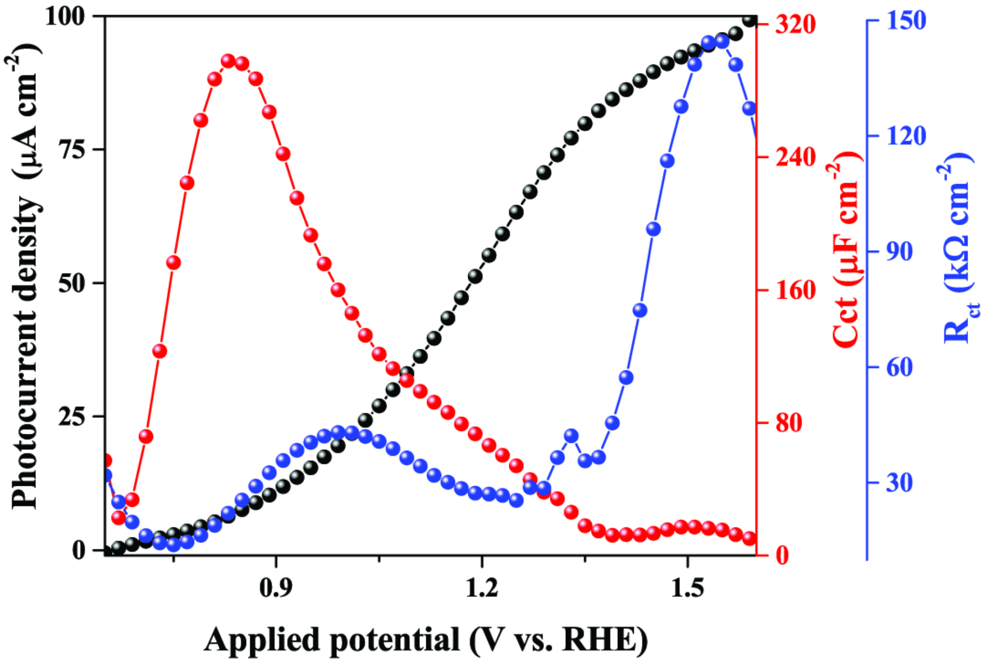

Supplementary Figure 38. Evolution of *J-V* (black spheres), surface state capacitance (red spheres) and charge transfer resistance (blue spheres) as a function of applied potential (illumination intensity: 100 mW cm^{-2}, pH 11.0).

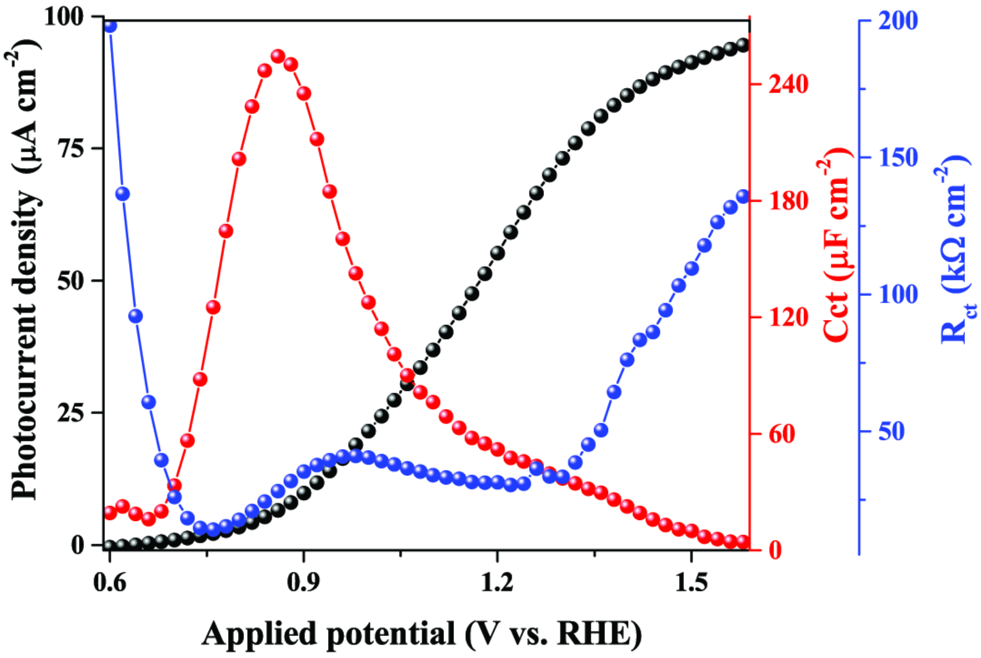

Supplementary Figure 39. Evolution of *J-V* (black spheres), surface state capacitance (red spheres) and charge transfer resistance (blue spheres) as a function of applied potential (illumination intensity: 100 mW cm^{-2}, pH 12.0).

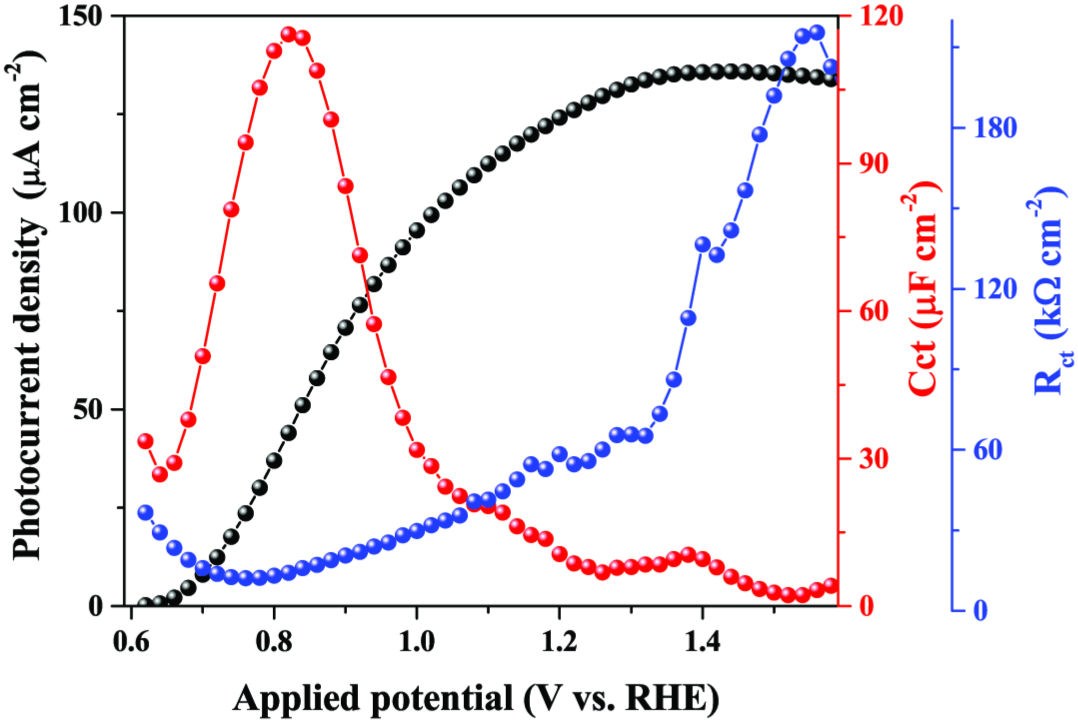

Supplementary Figure 40. Evolution of *J-V* (black spheres), surface state capacitance (red spheres) and charge transfer resistance (blue spheres) as a function of applied potential (illumination intensity: 100 mW cm^{-2}, pH 13.0).

4.2 Near steady-state CV analysis in buffered electrolyte

Similar CV measurements with a slow scan rate of 5 mV s^{-1} were conducted in the buffered electrolyte. The pseudo-peak at around 1.0-1.1 V vs RHE in unbuffered electrolyte at pH 11 completely disappeared under these conditions. In addition, photocurrent plateaus could even be observed at a low electrolyte pH of 8.

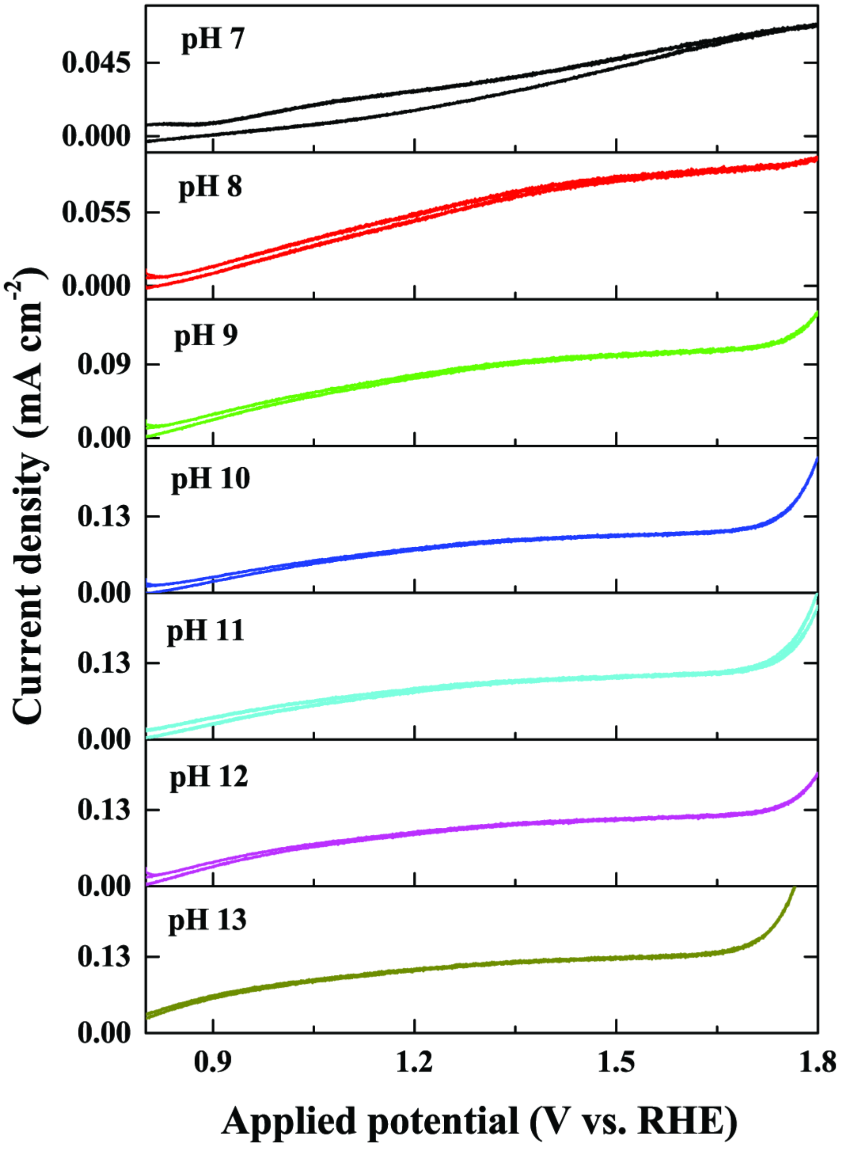

Supplementary Figure 41. CV profiles (illumination intensity: 100 mW cm^{-2}, scan rate: 5 mV s^{-1}) of a hematite photoanode measured in different electrolyte pH.

4.3 Transient photocurrent spectra in buffered electrolyte as a function of illumination intensity

The intensity dependent transition of surface protonation states completely disappeared in the buffered electrolyte, as indicated by transient photocurrent profiles in Supplementary Figure 42, the anodic decay half time in Supplementary Table 5, and the steady-state photocurrent change in Supplementary Figure 43.

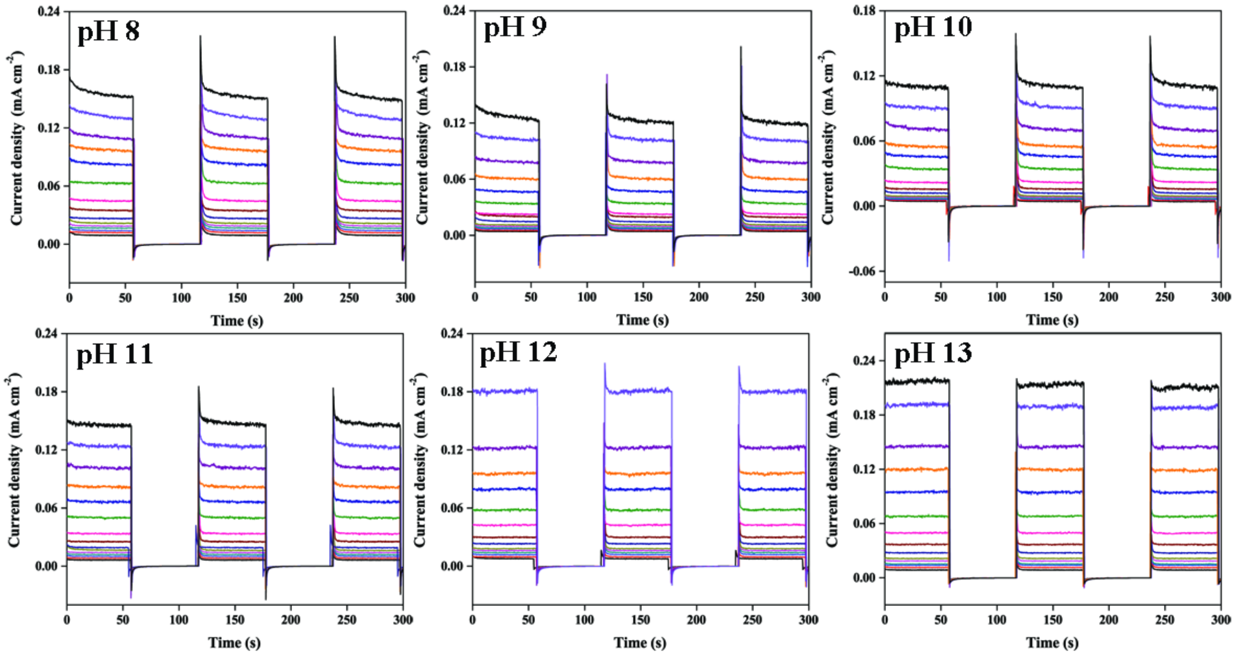

Supplementary Figure 42. Transient photocurrent profiles of hematite photoanode measured at different pH (8-13) and different illumination intensity (5-100 mW cm^{-2}, see Supplementary Table 5).

Supplementary Table 5. Anodic decay half time of hematite photoanode measured at different pH (8-13) and different illumination intensities (5-100 mW cm^{-2}).

Light intensity (mW cm^{-2})	$t_{1/2}$ (s)					
	pH 8	pH 9	pH 10	pH 11	pH 12	pH 13
5	3	1.0	1.0	1.0	0.8	1.0
6	1.6	0.8	1.0	1.0	0.8	0.8
7	1.6	0.8	1.0	0.8	0.6	0.6
8	1.2	0.6	0.8	0.6	0.6	0.4
9	1.2	0.4	0.8	0.6	0.6	0.4
10	1.0	0.4	0.6	0.6	0.4	0.4
12	1.0	0.4	0.4	0.6	0.4	0.4
15	0.8	0.4	0.2	0.4	0.4	0.4
20	0.8	0.4	0.2	0.4	0.4	0.4
30	0.8	0.2	0.2	0.4	0.2	0.2
40	0.8	0.2	0.2	0.4	0.2	0.2
50	0.8	0.4	0.2	0.2	0.4	0.6
60	0.8	0.2	0.4	0.6	0.4	0.6
80	0.8	0.4	0.6	0.6	0.6	0.6
100	0.8	0.6	0.6	0.8	0.6	0.4

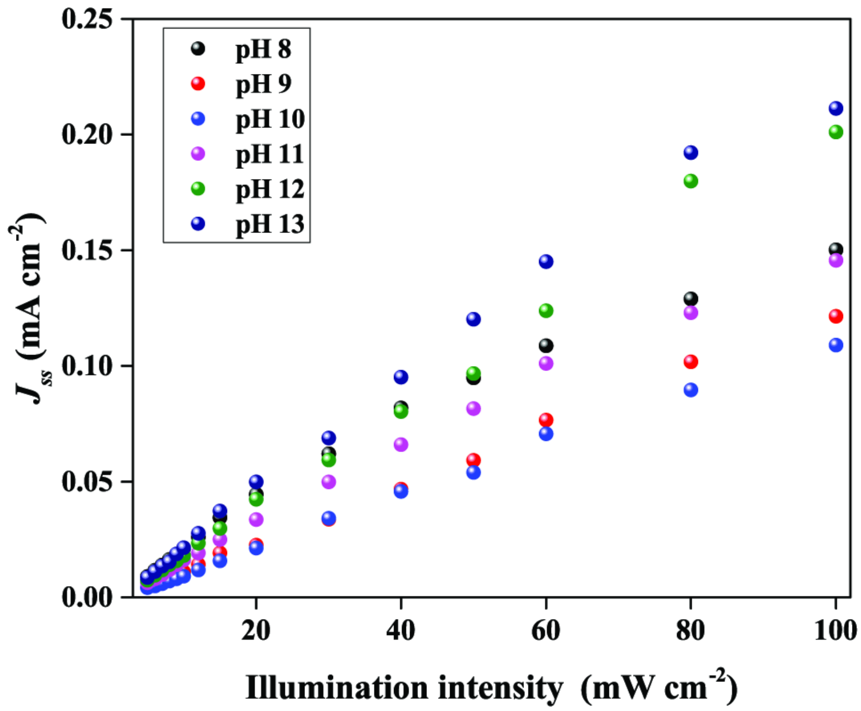

Supplementary Figure 43. Evolution of the steady-state photocurrent (J_{ss}) of a hematite photoanode measured at different pH (8-13) and different illumination intensities (5-100 mW cm^{-2}).

4.4 Transient photocurrent spectra in buffered electrolyte as a function of applied potential

The potential dependent transition of surface protonation states completely disappeared in the buffered electrolyte, as indicated by transient photocurrent profiles in Supplementary Figure 44, the anodic decay half time in Supplementary Table 6, and the steady-state photocurrent change in Supplementary Figure 45.

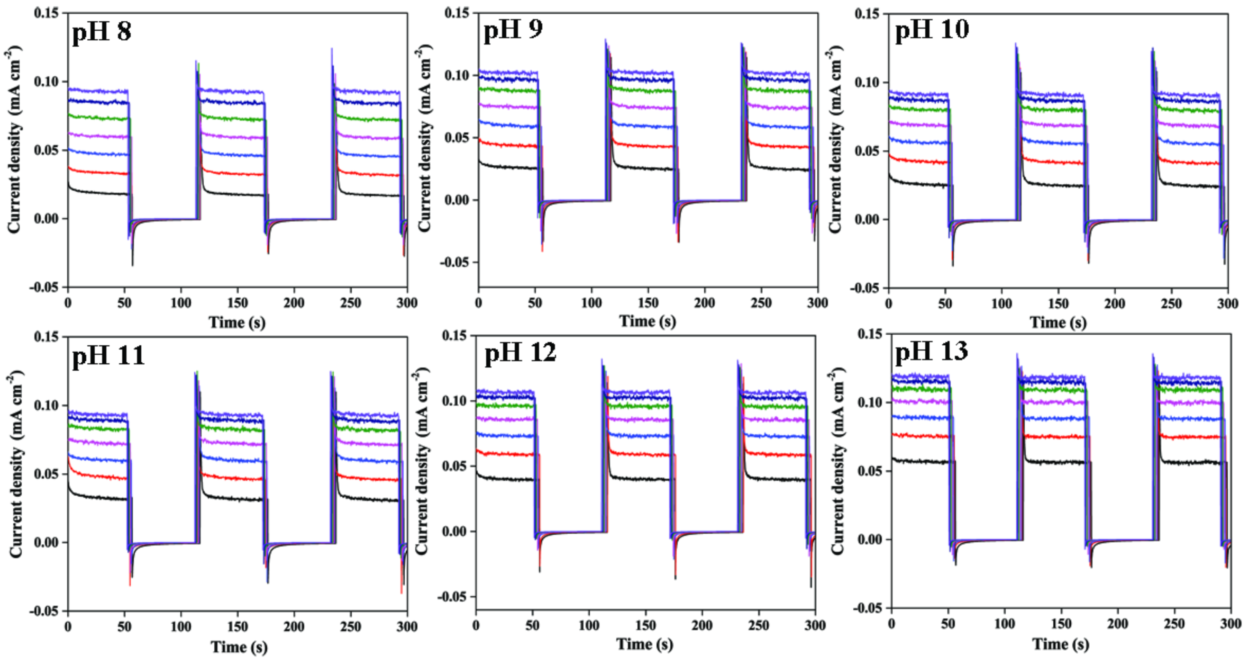

Supplementary Figure 44. Transient photocurrent profiles of hematite photoanode measured at different pH (8-13) and different applied potentials (0.9-1.5 V vs RHE, see Supplementary Table 6).

Supplementary Table 6. Anodic decay half time of hematite photoanode measured at different pH (8-13) and different applied potentials (0.9-1.5 V vs RHE).

Potential (V vs. RHE)	$t_{1/2}$ (s)					
	pH 8	pH 9	pH 10	pH 11	pH 12	pH 13
0.9	0.8	0.6	0.6	0.6	0.6	0.4
1.0	0.6	0.4	0.4	0.6	0.4	0.4
1.1	0.4	0.4	0.4	0.6	0.4	0.2
1.2	0.4	0.4	0.4	0.4	0.4	0.4
1.3	0.4	0.4	0.4	0.4	0.4	0.4
1.4	0.4	0.4	0.4	0.4	0.4	0.4
1.5	0.4	0.2	0.4	0.6	0.6	0.4

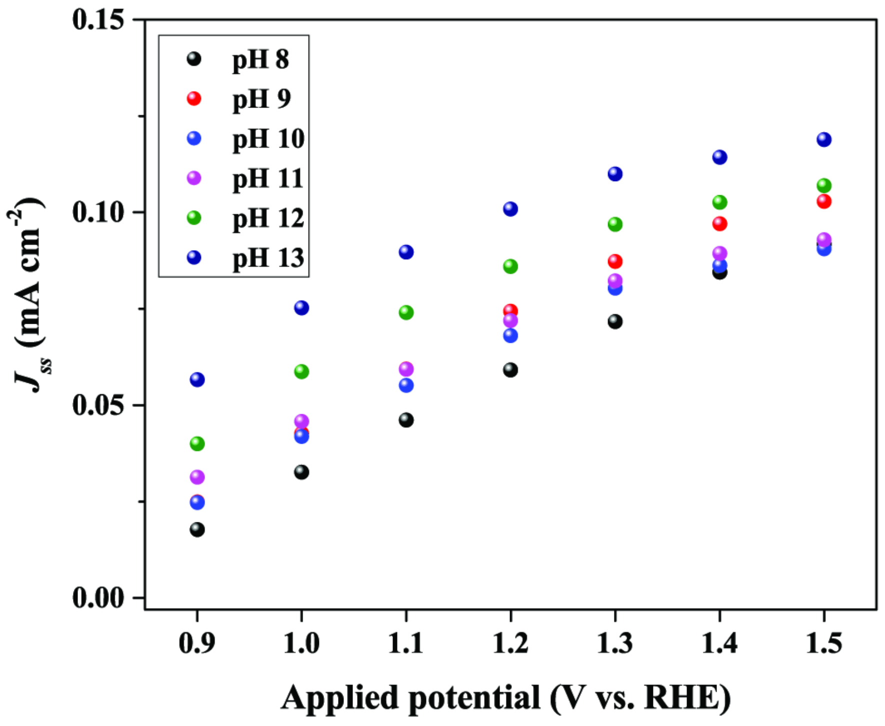

Supplementary Figure 45. Evolution of steady-state photocurrent (J_{ss}) of hematite photoanode measured at different pH (8-13) and different applied potentials (0.9-1.5 V vs RHE).

4.5 Hole transfer efficiency in buffered electrolyte

Similar to the calculations made for the unbuffered electrolyte, the hole transfer efficiency was also determined for the buffered electrolyte.

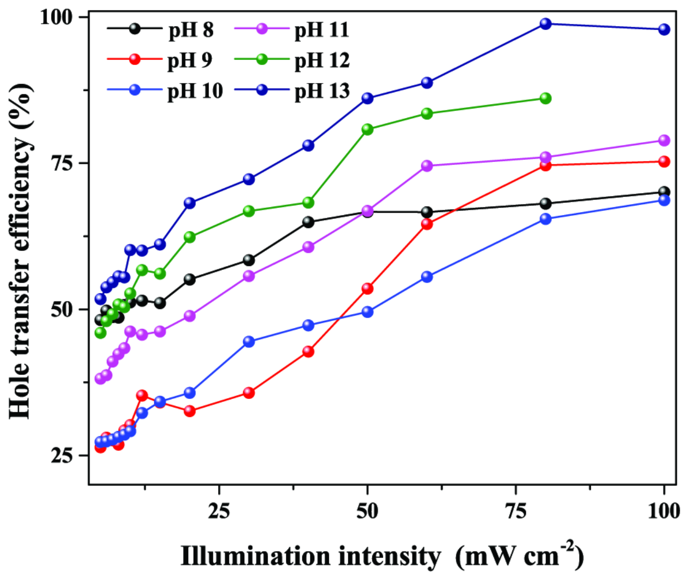

Supplementary Figure 46. Evolution of the hole transfer efficiency of hematite photoanode measured at different pH (8-13) and different illumination intensity (5-100 mW cm^{-2}).

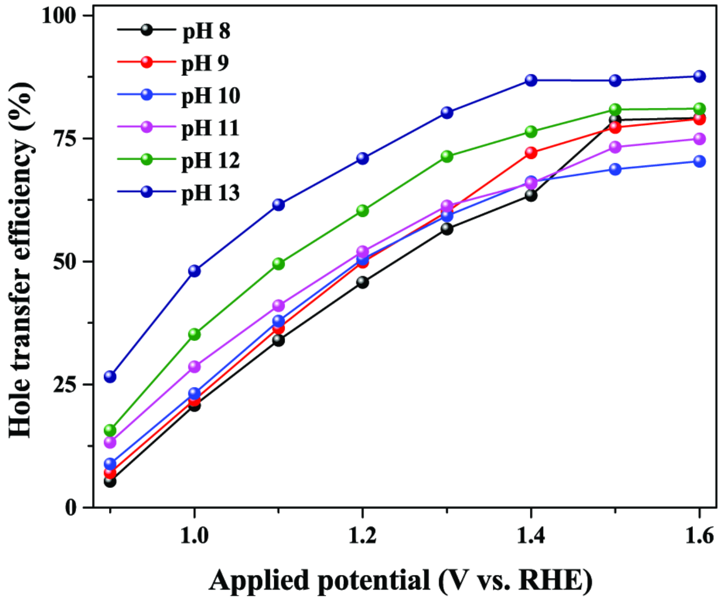

Supplementary Figure 47. Evolution of hole transfer efficiency of a hematite photoanode measured at different pH (8-13) and different applied potentials (0.9-1.5 V vs RHE).

4.6 Remarks on the TPS study in both buffered and unbuffered electrolyte at different pH values

During the transient photocurrent measurement, once the illumination is on, an anodic transient current is present and subsequently decays to the steady-state photocurrent. Similarly, a cathodic transient current will be present when the illumination is switched off and decays to the initial steady-state in the dark.[29] Multiple underlying dynamic processes such as charge accumulation in the hematite surface states, charge transfer to the electrolyte and electron hole recombination at the hematite-electrolyte interface, are responsible for the observed transient photocurrent evolution.[30] Generally, the anodic decay profile could be divided into two phases.[31,32] The fast decay phase is dominated by the charge accumulation and recombination processes and the slower decay phase represents the balance between charge transfer and recombination processes. In principle, both decay phases are controlled by the applied potential, illumination intensity and electrolyte characteristics, such as pH value. In the low pH region (pH 8-11), the buffered systems reach the steady-state photocurrent density more easily than the unbuffered ones, which highlights the capability of electrolyte buffers to keep the local environment constant (in terms of the protonation state on the hematite photoanode surface).[33] Therefore, both protonated and deprotonated micro surface regions contribute to the observed steady-state photocurrent density, where the surface distribution of hydroxyl ions determines their contribution. In the unbuffered electrolyte, however, the steady-state photocurrent density will not be established until the surface OH^- concentration is solely diffusion controlled. The slow decay phase is most pronounced near the PZC, which was determined as pH 11 in this study.

At high pH values (pH 12 and 13) for both buffered and unbuffered systems, the photoanode surface will be fully deprotonated and the overall rate is controlled by the catalytic reaction. Therefore, the steady-state photocurrent density is reached right after the fast decay phase. In addition, the evolution of the steady-state current densities in the unbuffered system is summarized in Supplementary Figure 24 (as a function of illumination intensity) and Supplementary Figure 27 (as a function of applied potential). Substantial transitions could be identified at pH 11 when the illumination intensity is around 30-40 mW cm^{-2} and the applied potential is around 1.1-1.2 V vs. RHE. Clearly, a switch from deprotonated to protonated surface state is responsible for this steady-state photocurrent transition. The significant steady-state photocurrent density gap between low pH values and high pH values for the unbuffered system further confirmed the fact that different protonation states are present. For the buffered system, there is a smoother transition for the steady-state photocurrent densities in the intermediate pH region, which indicates that the buffer pairs are capable of maintaining the local environment constant for the photoelectrochemical oxidation reaction.

5. Reproduction of results using transient photocurrent techniques

5.1 Profile of surface states probed by transient photocurrent techniques

Transient photocurrent spectroscopy is another powerful technique for investigating the charging and discharging processes of surface states.[34] Basically, the measurement starts from a steady-state in the dark, and once illumination is started, an anodic spike will be present and then decay to a new steady-state under illumination conditions. When the illumination is switched off, a similar cathodic spike will be present and the system will convert back to the initial steady-state in the dark. The amount of charges passed through the photoanode during the anodic transient process (Q_{anodic}) is the sum of surface stored (in surface states) and transferred charges (for water oxidation). Similarly $Q_{cathodic}$ can be integrated for the cathodic transient process, which is due to the discharge of some reactive and short-lived surface states (***S1*** and valence band holes in this case). In this way, the surface hole density can be estimated from $Q_{cathodic}$.

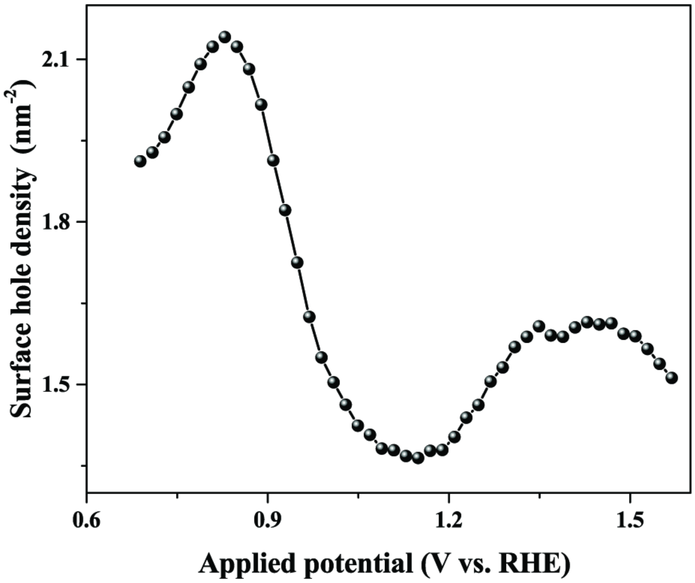

Supplementary Figure 48. Surface hole density evolution as a function of applied potential probed by transient photocurrent spectroscopy (illumination intensity: 100 mW cm^{-2}, pH 8.0).

5.2 Rate law analysis

Similar to illumination intensity modulated PEIS, upon changing the beam density, a series of surface hole density and corresponding reaction rates could be obtained. By substituting them into the rate equation, reaction orders of surface holes can be again calculated.

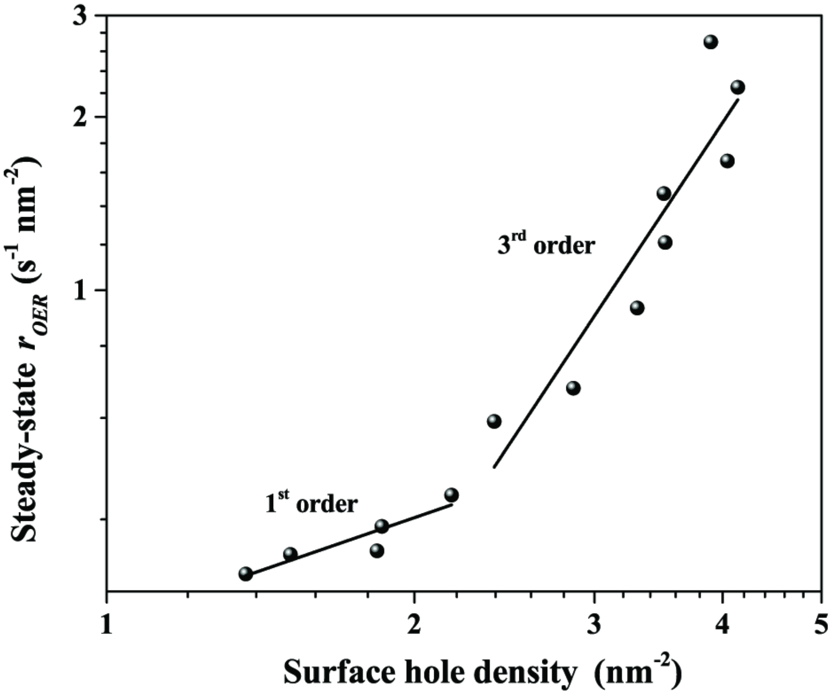

Supplementary Figure 49. Rate law analysis at 1.3 V vs RHE (pH 8) where ***S1*** reaches maximum values; steady-state OER rate (r_{OER}) and surface hole densities were probed by intensity modulated TPS.

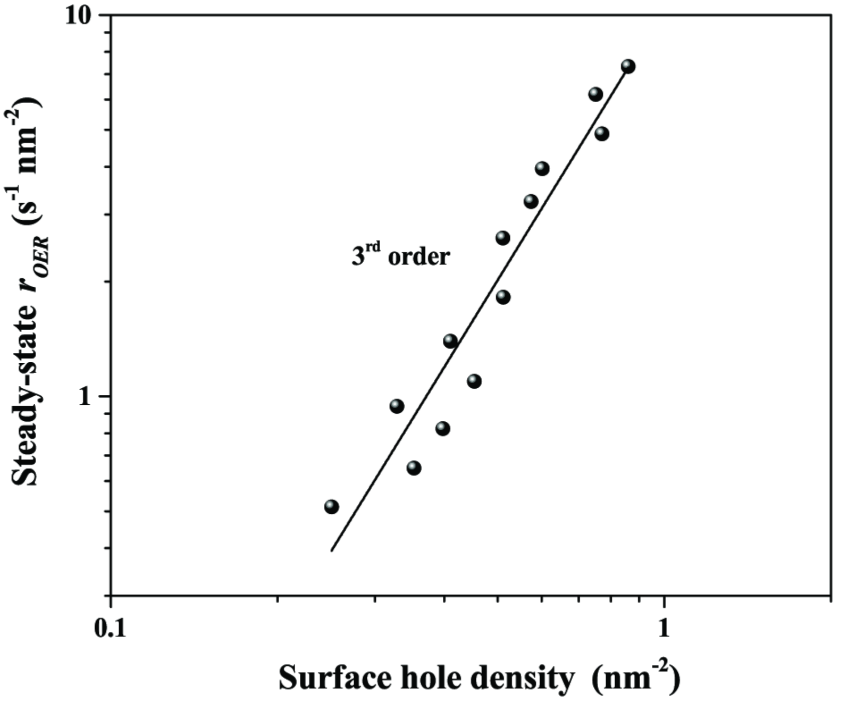

Supplementary Figure 50. Rate law analysis at 1.3 V vs RHE (pH 13) where ***S1*** reaches maximum values; steady-state OER rate (r_{OER}) and surface hole densities were probed by intensity modulated TPS.

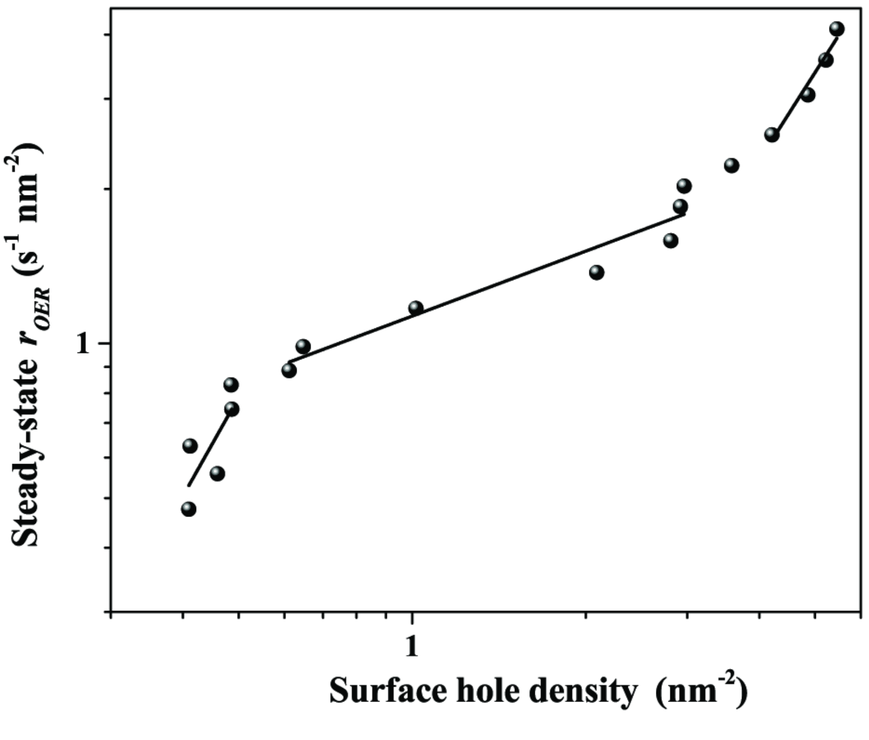

Supplementary Figure 51. Rate law analysis at 1.3 V vs RHE (pH 11) where ***S1*** reaches maximum values; steady-state OER rate (r_{OER}) and surface hole densities were probed by intensity modulated TPS.

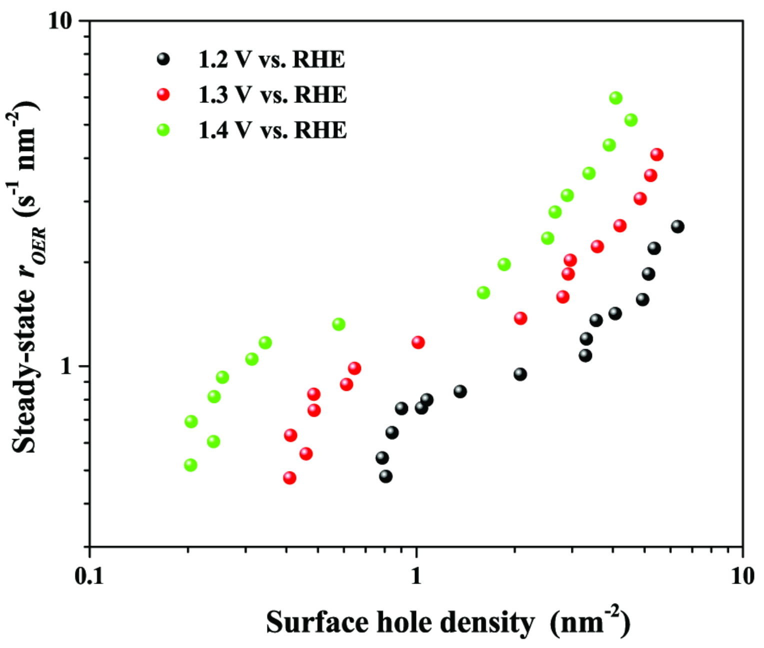

Supplementary Figure 52. Rate law analysis at different potentials (1.2-1.4 V vs. RHE, pH 11); steady-state OER rate (r_{OER}) and surface hole densities were probed by intensity modulated TPS.

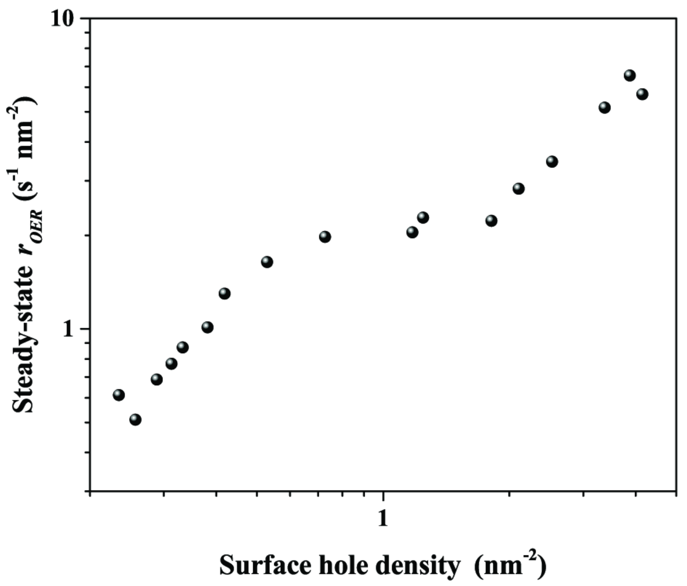

Supplementary Figure 53. Rate law analysis at 1.3 V vs RHE (pH 11, 0.5 M $NaClO_4$) where ***S1*** reaches maximum values; steady-state OER rate (r_{OER}) and surface hole densities were probed by intensity modulated TPS.

5.3 Pathways for photogenerated holes

Due to the limited hole diffusion length in hematite photoanodes (2-4 nm), photogenerated holes mainly recombine with conduction band electrons soon after their generation.[35,36] Only holes generated in the space charge region are able to diffuse to the valence band edge. These surviving valence band holes could either directly initiate water oxidation reaction through tunneling, or transfer to less energetic surface states (***S1*** in this case).[37] Since many studies have demonstrated the positive role of surface states during water oxidation, we here only consider the second pathway.[38,39] After surface holes arrive at ***S1***, they are now facing the same options as they do at the valence band edge, i.e. directly mediating water oxidation or a transfer to less energetic ***S2***. If they are following the first route, then a 1st order reaction would be expected. In contrast, 3rd order reaction kinetics would appear when the second approach is dominant. The transformation from ***S1*** to ***S2*** requires the accumulation of at least three equivalent holes. Therefore, the process is influenced by factors like surface hole density, hole diffusion barrier, and hole lifetime. Consequently, several experimental parameters can tune these three factors. Specifically, these are: (a) the illumination intensity is able to modulate the population of holes arriving the surface; (b) the electrolyte pH is capable of regulating the surface protonation state, thereby influencing the hopping speed of surface holes; (c) the applied potential could prolong the lifetime of surface holes by controlling the conduction band electron density. Apparently, the distribution of surface states and their transformation is controlled by the interplay of the above-mentioned experimental conditions. Therefore, it is easy to understand why different investigations sometimes give rise to controversial conclusions. Clearly, resolving the complex overall picture of this dynamic process is extremely difficult, but indispensable for understanding the working mechanism of a PEC cell, and of relevant photoelectrode processes. We hope that our study will inspire more investigations into the dynamic realm of PEC devices.

6. Proposed OER mechanism

At pH 8 (protonated surface) and low illumination intensity, the observed 1st order reaction at 1.3 V vs. RHE could be explained by the proposed reaction pathway shown in Figure 4e. When the surface hole density is low, the nucleophilic addition of water molecule to the isolated ***S1*** site (Fe^{IV}=O) is the rate determining step (RDS), meanwhile, a proton is released. When the illumination intensity surpasses a certain value (8-10 mW cm^{-2}), the increased surface hole density gives rise to a 3rd order reaction instead. In this case, the RDS takes place after three sequential hole accumulation steps at the neighboring Fe^{III}-OH sites (Figure 4 f). At pH 13, however, the deprotonated surface facilitates the fast migration of surface holes, and the RDS always takes place after accumulation of three surface holes (Figure 4 g), so that a 3rd order reaction kinetics is determined from rate law analysis. The RDS assignment for the 3rd order reaction

kinetics was further verified with kinetic isotope effect (KIE) determinations. Since only a negligible KIE was observed over a wide range of electrolyte pH (Supplementary Figure 54), the cleavage of the O-H bond could be excluded and consequently the transformation of the $Fe^{IV}(=O)-O-Fe^{IV}(=O)$ to a bridged peroxo species is proposed as RDS.

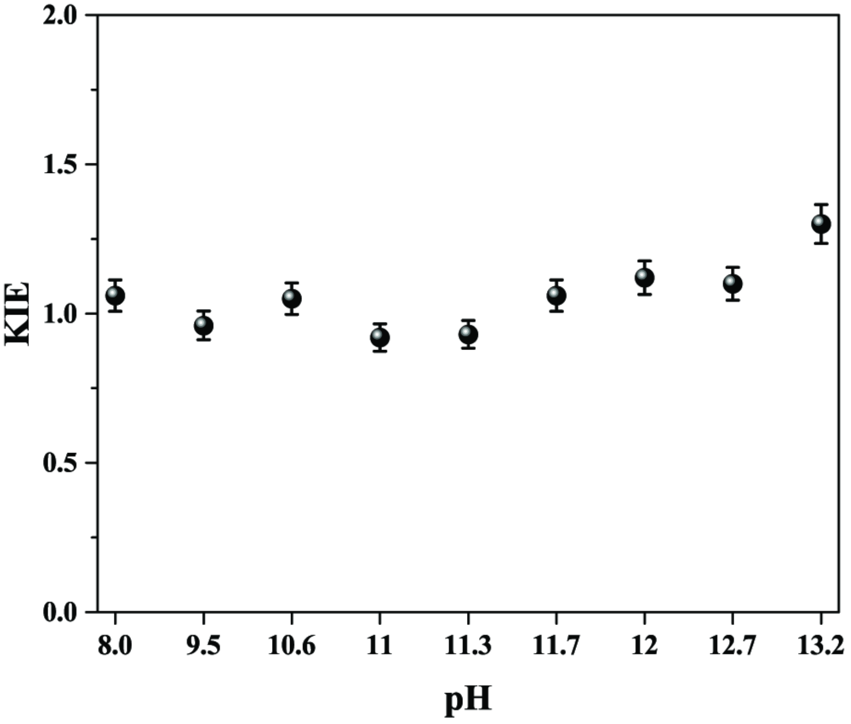

Supplementary Figure 54. Comparison of KIE when using D_2O instead of H_2O as the electrolyte solvent at different pH values. The negligible KIE indicates that no cleavage of O-H bonds is involved in the RDS. All data are presented as mean values ± standard deviation.

7. Supplementary Discussion

7.1 Supplementary Discussion I: Density of surface holes for rate law analysis

When performing the rate law analysis, we employed an alternative electrochemical way for estimating the density of surface holes. This approach assumed an ideal capacitor at the semiconductor-electrolyte interface and the corresponding charge transfer capacitance was resolved employing a simplified equivalent circuit (Supplementary Figure 6). In this model, the surface potential is proportional to the resistance fraction of charge transfer components. The 'applied potential' is the overall potential difference between working electrode and reference electrode which is controlled by the potentiostat. Therefore, the 'applied

potential' is an absolute value, and the applied equation can estimate the corresponding surface hole density in this case.[6] However, we have to point out that the derived density of surface holes does not represent their 'real value', because i) the equivalent circuit failed to separate charges that are resting in the surface and sub-surface (mostly referring to the band-bending region depending on the applied potential), and ii) the 'surface potential' estimation from Supplementary Equation 3 is based on the equivalent circuit which is a simplified physical model. In this case, a pre-factor should be placed in front of the interrogated value, $[h^+]_{surface} = a[h^+]_{interro}$. This pre-factor '*a*' could adjust the measured values of surface state capacitance and surface potential. We assume that the pre-factor *'a'* is a constant and is independent of the excitation density. Therefore, the derived reaction orders will not be influenced by the pre-factor *'a'* during the logarithmic operation, and they are rather determined by the intrinsic change rate of surface holes upon different excitations. Therefore, we see no problems to apply these derived reaction orders to re-construct the catalytic cycle and to predict the related intermediate species.

Indeed, deriving the surface concentration of holes is experimentally challenging. The Durrant group, for example, employed transient absorption spectroscopy to probe the density of surface holes, and then used the results for the interpolation of reaction kinetics. This approach, however, is based on the assumption that the absorption is primarily from the surface, not from the band-bending region or bulk. Nevertheless, the derived reaction kinetics could coherently explain their observed catalytic phenomena. Regardless of the absolute values of surface holes, the reaction orders as we derived here with two different electrochemical methods are in line with transient absorption techniques.[6,7,13,40,41] This coherent observation fully supports our conclusions. However, pursuing the 'real' density of surface holes is urgent for future in-depth understanding.

In order to obtain the 'real value' of the surface hole density, the first step is to probe the 'surface potential'. Techniques like dual working electrode (DWE) and Kelvin probe force microscopy are employed to estimate the 'surface potential'. However, they are actually not probing the 'surface potential' only. For example, DWE probes the potential difference between semiconductor top layer (the one facing the electrolyte) and bottom layer (the one facing the Au substrate layer). Therefore, the probed value is the combination of 'surface potential' and 'bulk potential' of the semiconductor. Until now, there are no experimental techniques reported which specifically probe the 'surface potential', to the best of our knowledge.

7.2 Supplementary Discussion II: Competition of interfacial hole transfer with back-electron recombination (BER)

In the present study, it became clear that both forward transfer and BER are coexisting at the potentials we applied (as evidenced by the negative transient peaks in Supplementary Figures 25, 26 and 28 etc.). This is exactly the reason why we observed very low photocurrents (Figure 1d and Figure 3a). In this case, the photo-generated holes are predominantly recombined with conduction band electrons at the semiconductor-electrolyte interface. Upon external potential elevation, application of alkaline electrolytes or under intense illumination, the BER process could be slowed down (Supplementary Figures 29-30). We notice that Le Formal *et al.* adopted a sufficiently high potential in order to completely retard the competitive interfacial BER.[7] By doing so, forward transfer is the only way for surface holes, which significantly facilitates the interpretation of photoinduced absorption (PIA) decay over time (after the light is switched off). Therefore, the evolution of reaction orders with respect to surface holes could be derived during the decay of the PIA signal. The transition of reaction orders during this dark decay agrees with the results obtained by Le Formal *et al.*, which is based on PIA signal and hole transfer rate at steady-state illumination (PIA vs. current).[7] This agreement adds further evidence that these surface accumulated holes are indeed not undergoing interfacial BER. When a lower external potential is applied, surface holes are not exempted from interfacial recombination any more. In this case, the rate law analysis approach (PIA vs. current) is still valid for interpreting the reaction orders as it is based on steady-state conditions (in other words, the density of surface holes does not change over time). The derivation of reaction orders during the PIA signal decay, however, may become problematic as the change of surface hole density over time is dependent on not only the forward transfer rate, but also the competitive BER rate.

We adopt PEIS techniques to determine the surface hole density and the corresponding transfer rate at pseudo-steady-state conditions (the magnitude of the small AC voltage perturbation is negligible compared to the DC applied voltage). This means that both surface hole density and transfer rate are not changing over time. By applying different excitation intensities, a wide range of steady-state surface hole densities and corresponding transfers rate values could be obtained for rate law analysis. This may be the reason why we observed similar reactions orders and their transition upon surface hole accumulation as has been reported in the study of Le Formal *et al.*[7]

In addition, previous work has demonstrated clearly that pronounced correlations were observed when measuring surface charges with different techniques.[20] This implies that coherent reaction orders and their transitions should be observed regardless of probing techniques. This coherence is indeed evidenced by rate law analyses in this work (employing PEIS and transient photocurrent spectra) and in the study of Le Formal

et al. (employing PIA).[7,13] Further, PEIS is also capable of quantifying the relative contributions of surface hole transfer and recombination in term of rate constants.[28,37] Since we are primarily interested in studying reaction kinetics and their interplay with surface states on hematite, we place less emphasis on the discussion of the recombination kinetics here.

We do observe a positive steady-state photocurrent (although very low) when the capacitance of *S2* reaches its maximum. Additionally, although *S2* is lower in energy, reaction kinetics need to be taken into consideration, especially at higher external applied potentials. Coincidentally, a previous study proposed a low activation energy reaction pathway in addition to the Fe^{IV}=O mediated energy demanding process.[13] Therefore, we linked our observations to these results and proposed that the chemical signature of *S2* is a peroxo species.

7.3 Supplementary Discussion III: Resolving the chemical nature of *S2* with operando spectroscopy

We propose that the chemical identity of *S2* is the surface peroxo intermediate which is generated through a three hole oxidation process. In addition, we have made attempts to resolve the chemical nature of *S2* directly with operando ATR-FTIR techniques but we could not achieve the vibrational signature for the following reasons: (i) we could only apply low illuminations during the spectroscopic measurements, as the spectroscopic cell has to be isolated somehow in order to prevent the isotope exchange of the electrolyte with the environment. Therefore, the fast third order reaction kinetics could not be initiated and the concentration of *S2* was limited. (ii) The electrolyte layer between the ZnSe crystal top surface and the hematite working electrode was too thick for the emerging waves to penetrate, so that the surface species were out of reach. (iii) The nanostructured hematite photoanode is not suitable for surface intermediate species analysis, since it provides limited accessible area for the emerging waves. (iv) Either the molecular concentration of *S2* is very low or the molar attenuation coefficient of *S2* is very small (depending upon its chemical composition and structure), so that it is very difficult to gain sufficient spectroscopic absorption for *S2* according to the Lambert-Beer law. (v) The interference of electrolyte signal, especially for many commonly used electrolytes, such as $NaClO_4$ or Na_2SO_4, could potentially overlap with the intermediate signal around 900-1100 cm^{-1}. Therefore, we are trying to use KCl or NaF as supporting electrolyte instead.

Nevertheless, we are still aiming to resolve the vibrational signature of *S2* using operando ATR-FTIR. There are a few technical difficulties to overcome at the moment before we can report a valid signal.

7.4 Supplementary Discussion IV: Reasons for the use of native hematite as a model system

In order to investigate the intrinsic surface properties, such as surface states dynamics herein, we here employed native hematite as a model system. Our hematite photoanodes were not very active compared to some previous publications where hematite was intentionally doped through either high-temperature calcination (800 °C for 20 min) or extrinsic doping.[42–45] These doping strategies most likely altered the energetics and distributions of both surface states, which would have hindered the investigation of their true intrinsic behavior in our study. In short, such devices could not be employed as native 'state-of-the-art' hematite. This is the reason why we consistently used the relatively low performance hematite. In addition, we underscore that the photocurrent (Figure 1d) was measured in pH 8 after 1 min stabilization to reach a steady-state. The test conditions, especially the electrolyte pH, exert a significant influence on the reported photocurrent value. For example, our device also exhibited notably enhanced activity in 1 M NaOH (Supplementary Figure 11) compared to the one tested in pH 8. The essential role of the electrolyte pH was also further investigated in our study, where the surface protonation state could modulate the interplay of the two types of surface states and their recombination dynamics.

Meanwhile, we want to highlight that the preparation, pre/post-treatment histories and the performance evaluation conditions of hematite photoanodes exert a significant influence on the reported values. For facile and clear comparison, we prepared the following Supplementary Table 7 to compare our values with reference values. From Supplementary Table 7 we can conclude that i) the electrolyte pH exerts a dominant effect on the reported performance (when comparing this work to ref. 11), ii) the TiO_2 pretreatment of FTO could promote the activity of hematite to a large extent (ref. 46), iii) the post annealing temperature and time span (especially the high temperature calcination at 800 °C) would alter the extent of Sn diffusion from substrate FTO to hematite. Overall, we feel that the performance of our hematite is comparable to previous investigations if preparative histories and testing conditions are properly taken into account.

Supplementary Table 7. Performance comparison of hematite photoanode in this study vs. related references.

Fabrication method	Post/pre-treatment	Electrolyte	Illumination geometry	J_{photo}@1.23 V vs. RHE [a,b]	Ref.
Atomic layer deposition (~60 nm)	N.A.	pH 6.9 (0.1 M phosphate buffer with 0.2 M KCl)	Back-side	~0.01 mA cm^{-2}	11
Atomic layer deposition (~60 nm)	N.A.	pH 13.3 (0.1 M KOH with 0.2 M KCl)	Back-side	~0.10 mA cm^{-2}	11
Two-step hydrothermal growth	600 °C for 30 min	pH 13.6 (1 M NaOH)	N.A.	~0.10 mA cm^{-2}	46
Two-step hydrothermal growth	TiO_2 modified FTO, 600 °C for 30 min	pH 13.6 (1 M NaOH)	N.A.	~0.51 mA cm^{-2}	46
One-step hydrothermal growth	Annealed at 550 °C for 1 h, then 800 °C (20 min)	pH 13.6 (1 M NaOH)	N.A.	~0.20 mA cm^{-2}	1
One-step hydrothermal growth	Annealed at 600 °C for 2 h, then 800 °C (5 min)	pH 8.0 (0.05 M $NaClO_4$)	Front-side	~0.01 mA cm^{-2}	This work
One-step hydrothermal growth	Annealed at 600 °C for 2 h, then 800 °C (5 min)	pH 13.6 (1 M NaOH)	Front-side	~0.13 mA cm^{-2}	This work

a. All data were obtained under illumination of AM 1.5 G 100 mW cm^{-2}.

b. For the performance determination, we have aged our device 1 min for every data point. The scan rate has not been indicated in other refs. of this table.

7.5 Supplementary Discussion V: Faradaic efficiency of OER on hematite electrodes

The Faradaic efficiency of oxygen evolution on the herein applied hematite photoanodes was measured at different pH (8-13) and applied potentials (1.3-1.6 V vs. RHE). The experiment was conducted in a home-designed air-tight photoelectrochemical cell employing a hematite photoanode as working electrode, Pt rods and saturated Ag/AgCl as counter and reference electrode, respectively. Prior to every measurement, the electrolyte was degassed with Ar for 15 min. The oxygen concentration change in the electrolyte during photo-electrolysis was monitored with a fluorescence-based oxygen sensor (Pyroscience). Faradaic efficiency was calculated as the ratio of measured amount of oxygen change (in terms of corresponding charge, Q_{O2}) and the theoretical amount of oxygen generation (also in form of charge, Q_{total}). Q_{total} was determined by integration of the photocurrent over time (Supplementary Figures 55-60). It is clear that Q_{O2} and Q_{total} values are steadily increasing and remain largely comparable over time under all test conditions with respect to pH values and applied potentials. This means that the Faradaic efficiency of oxygen evolution on our hematite electrodes is close to unity. In addition, we must take into account that there is always a tiny amount of atmospheric oxygen leakage into the reaction system despite excessive encapsulation of all caps. This probably leads to the observed abnormal higher Q_{O2} (compared to Q_{total}) for measurements which are conducted at lower pH values and lower applied potentials. We also performed blank measurements (without photo-electrolysis) in order to quantify the rate of oxygen leakage. Despite all these measures, we still cannot completely exclude inherent experimental errors from individual

operations. Probably a more sophisticated air-tight photoelectrochemical cell design in the future could help to eliminate this system error.

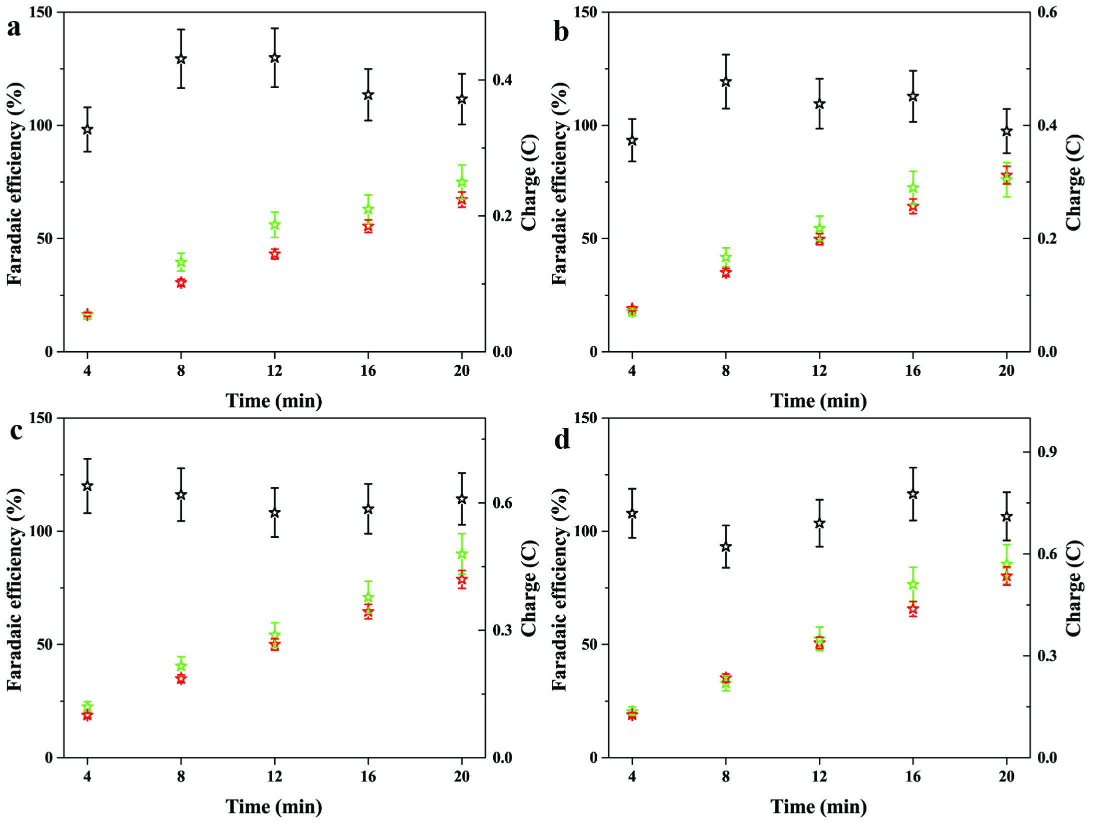

Supplementary Figure 55. Faradaic efficiency of oxygen evolution on hematite photoanodes in pH 8 electrolyte under different applied potentials (black stars, left y-axis): 1.3 (a), 1.4 (b), 1.5 (c) and 1.6 V vs. RHE (d) and simulated solar illumination. The corresponding measured Q_{O2} and theoretical Q_{total} values are plotted as red and green stars, respectively (right y-axis). All data are presented as mean values ± standard deviation.

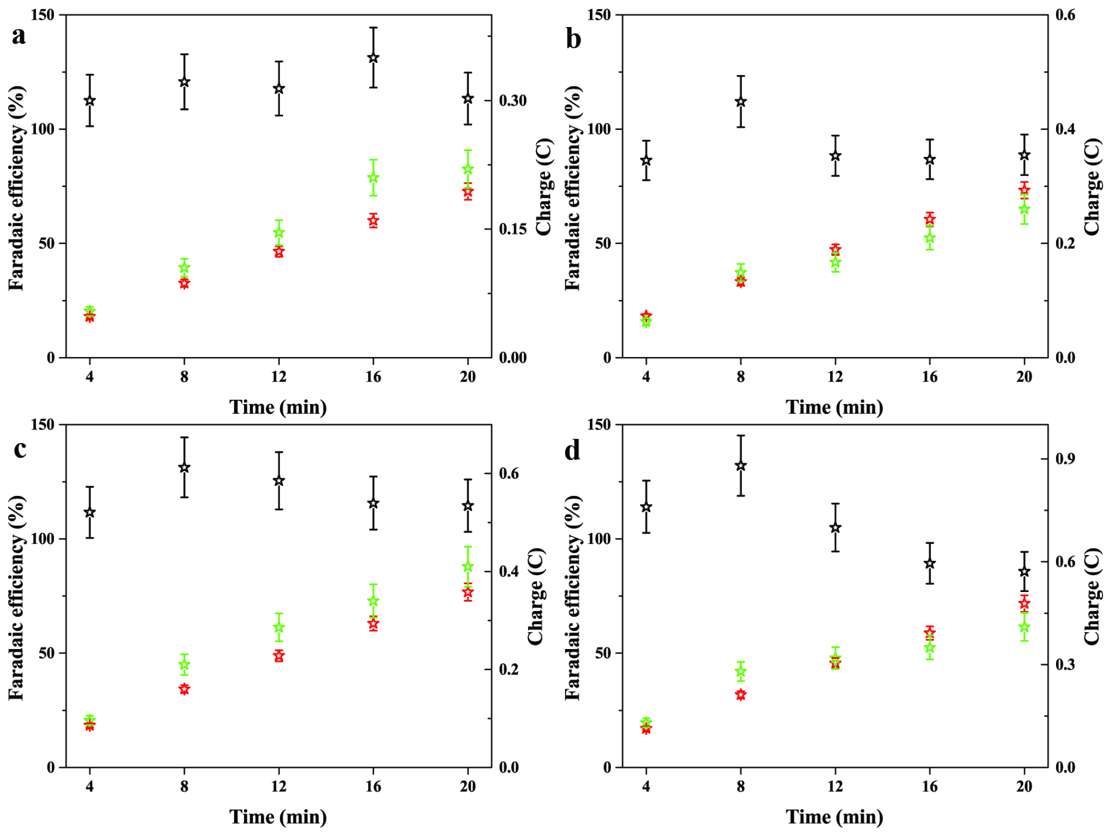

Supplementary Figure 56. Faradaic efficiency of oxygen evolution on hematite photoanode in pH 9 electrolyte under different applied potentials (black stars, left y-axis): 1.3 (a), 1.4 (b), 1.5 (c) and 1.6 V vs. RHE (d) and simulated solar illumination. The corresponding measured Q_{O2} and theoretical Q_{total} values are plotted as red and green stars, respectively (right y-axis). All data are presented as mean values ± standard deviation.

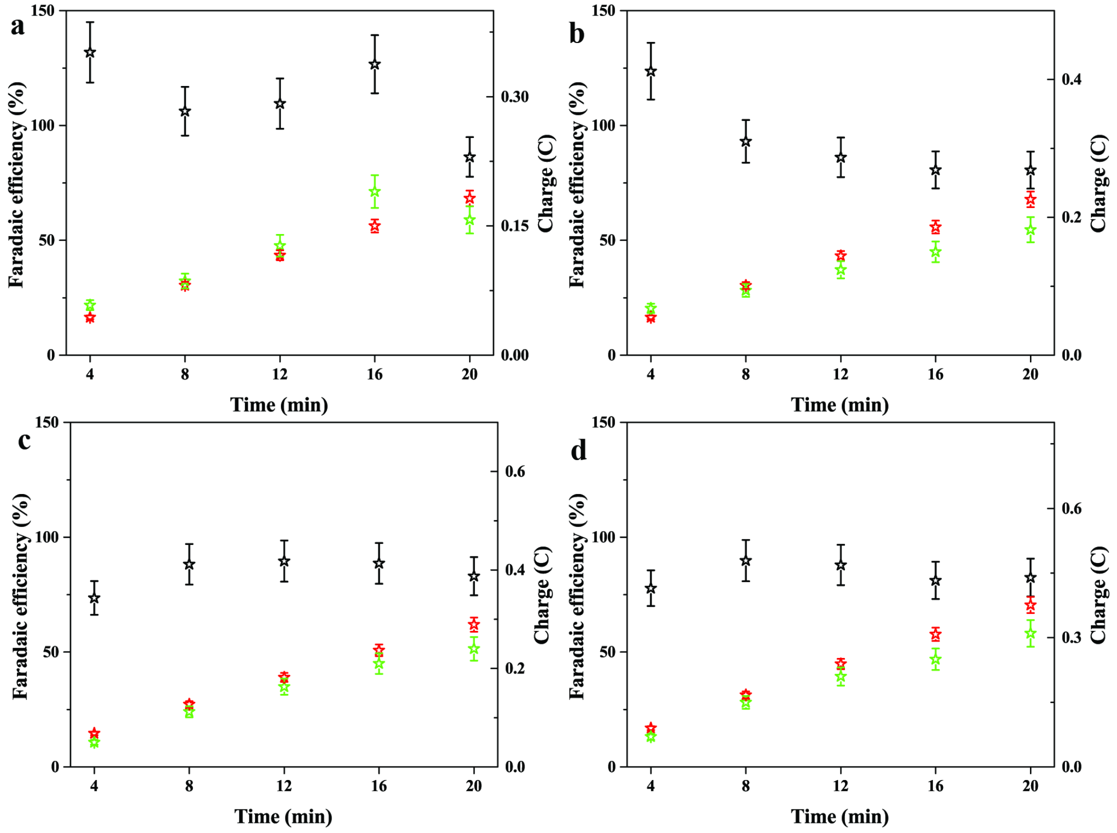

Supplementary Figure 57. Faradaic efficiency of oxygen evolution on hematite photoanode in pH 10 electrolyte under different applied potentials (black stars, left y-axis): 1.3 (a), 1.4 (b), 1.5 (c) and 1.6 V vs. RHE (d) and simulated solar illumination. The corresponding measured Q_{O2} and theoretical Q_{total} values are plotted as red and green stars, respectively (right y-axis). All data are presented as mean values ± standard deviation.

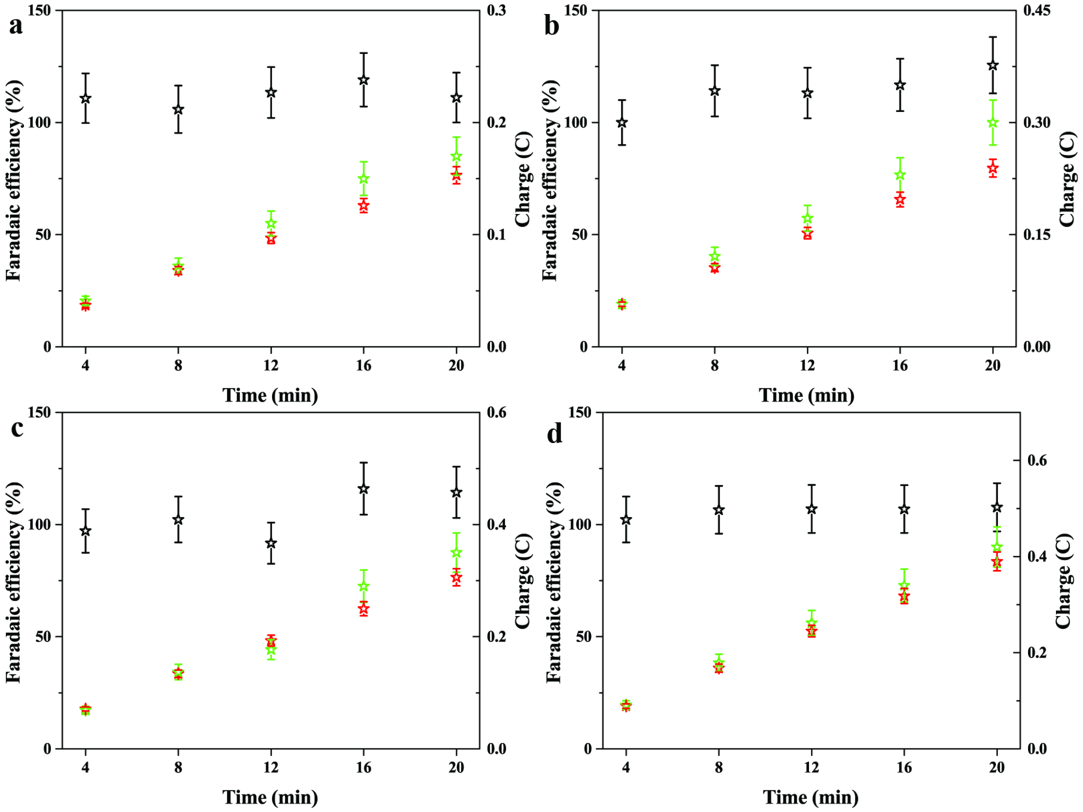

Supplementary Figure 58. Faradaic efficiency of oxygen evolution on hematite photoanode in pH 11 electrolyte under different applied potentials (black stars, left y-axis): 1.3 (a), 1.4 (b), 1.5 (c) and 1.6 V vs. RHE (d) and simulated solar illumination. The corresponding measured Q_{O2} and theoretical Q_{total} values are plotted as red and green stars, respectively (right y-axis). All data are presented as mean values ± standard deviation.

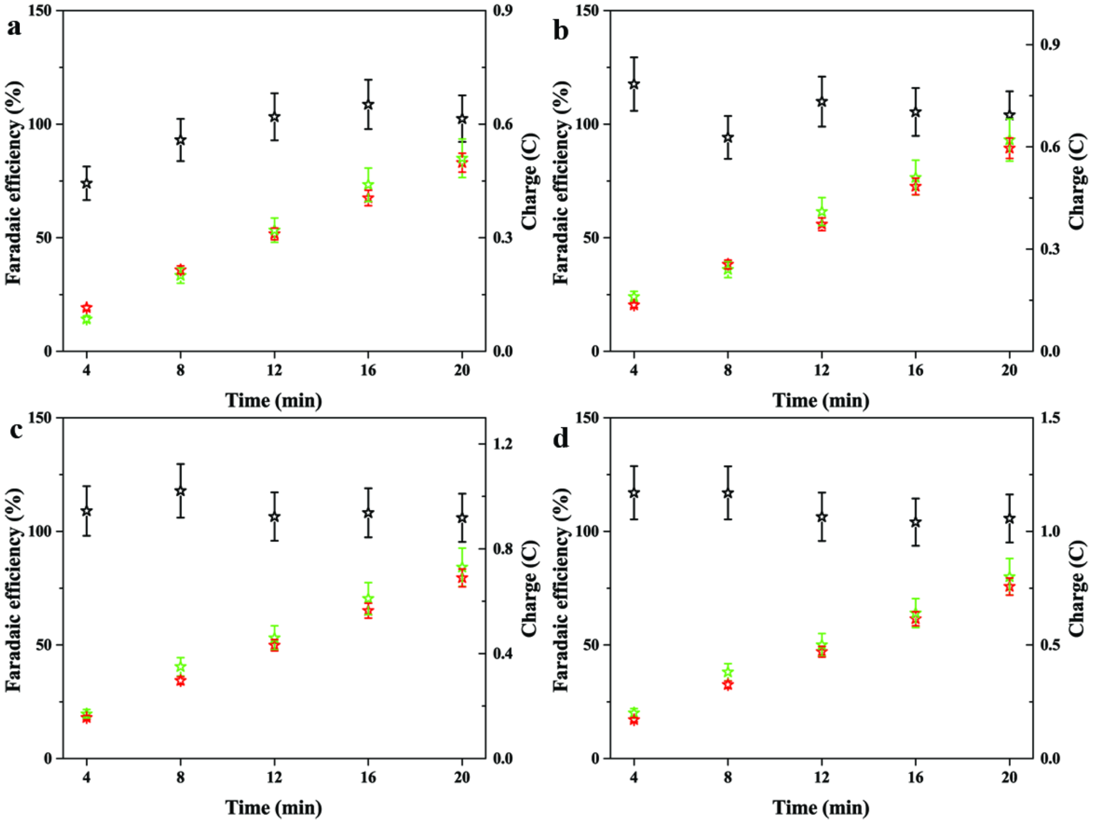

Supplementary Figure 59. Faradaic efficiency of oxygen evolution on hematite photoanode in pH 12 electrolyte under different applied potentials (black stars, left y-axis): 1.3 (a), 1.4 (b), 1.5 (c) and 1.6 V vs. RHE (d) and simulated solar illumination. The corresponding measured Q_{O2} and theoretical Q_{total} values are plotted as red and green stars, respectively (right y-axis). All data are presented as mean values ± standard deviation.

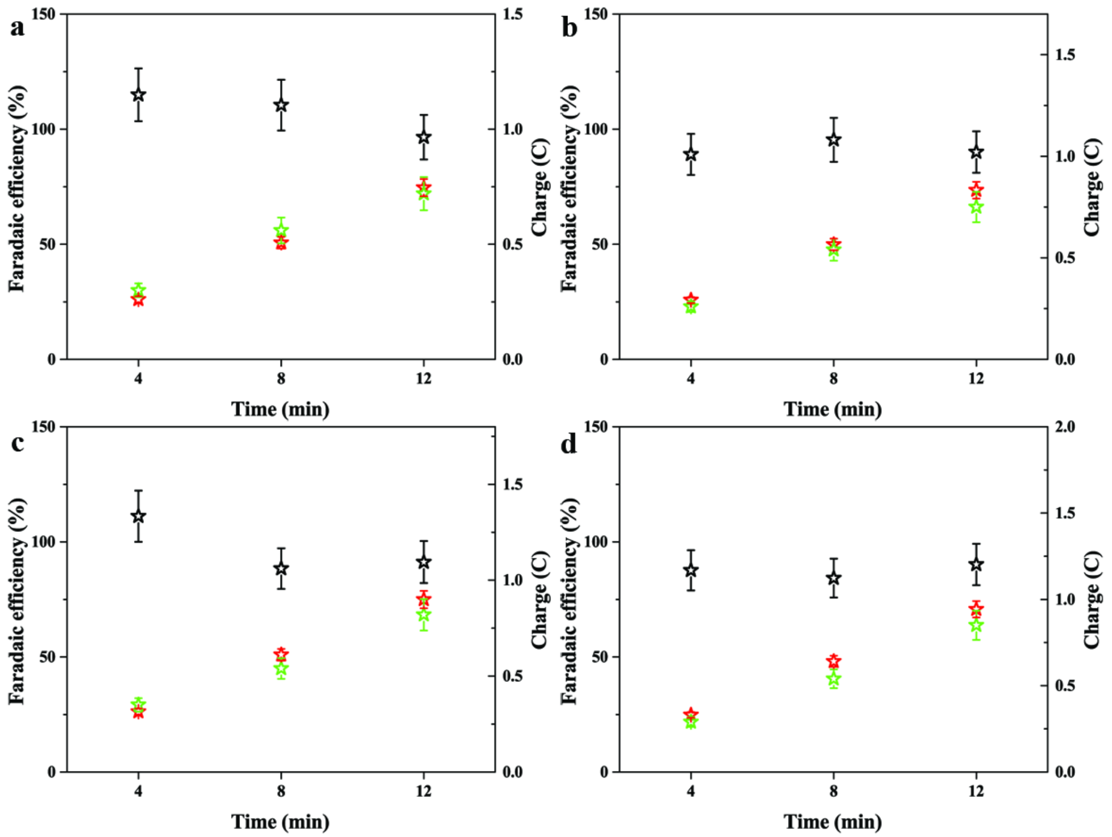

Supplementary Figure 60. Faradaic efficiency of oxygen evolution on hematite photoanode in pH 13 electrolyte under different applied potentials (black stars, left y-axis): 1.3 (a), 1.4 (b), 1.5 (c) and 1.6 V vs. RHE (d) and simulated solar illumination. The corresponding measured Q_{O2} and theoretical Q_{total} values are plotted as red and green stars, respectively (right y-axis). All data are presented as mean values ± standard deviation.

8. References

1. Ahmed, M. G. *et al.* Enhanced Photoelectrochemical Water Oxidation on Nanostructured Hematite Photoanodes via p-$CaFe_2O_4$/n-Fe_2O_3 Heterojunction Formation. *J. Phys. Chem. C* **119,** 5864–5871 (2015).

2. Vayssieres, L., Beermann, N., Lindquist, S.-E. & Hagfeldt, A. Controlled Aqueous Chemical Growth of Oriented Three-Dimensional Crystalline Nanorod Arrays: Application to Iron(III) Oxides. *Chem. Mater.* **13,** 233–235 (2001).

3. Grave, D. A., Yatom, N., Ellis, D. S., Toroker, M. C. & Rothschild, A. The "Rust" Challenge: On the Correlations between Electronic Structure, Excited State Dynamics, and Photoelectrochemical Performance of Hematite Photoanodes for Solar Water Splitting. *Adv. Mater.* **30,** e1706577 (2018).

4. Bisquert, J. Chemical capacitance of nanostructured semiconductors. Its origin and significance for nanocomposite solar cells. *Phys. Chem. Chem. Phys.* **5,** 5360 (2003).

5. Bertoluzzi, L. & Bisquert, J. Equivalent Circuit of Electrons and Holes in Thin Semiconductor Films for Photoelectrochemical Water Splitting Applications. *J. Phys. Chem. Lett.* **3,** 2517–2522 (2012).

6. Zhang, Y. *et al.* Rate-Limiting O-O Bond Formation Pathways for Water Oxidation on Hematite Photoanode. *J. Am. Chem. Soc.* **140,** 3264–3269 (2018).

7. Le Formal, F. *et al.* Rate law analysis of water oxidation on a hematite surface. *J. Am. Chem. Soc.* **137,** 6629–6637 (2015).

8. Zhang, J. & Eslava, S. Understanding charge transfer, defects and surface states at hematite photoanodes. *Sustain. Energ. & Fuels* **4,** 2724 (2019).

9. Iandolo, B., Wickman, B., Zorić, I. & Hellman, A. The rise of hematite: origin and strategies to reduce the high onset potential for the oxygen evolution reaction. *J. Mater. Chem. A* **3,** 16896–16912 (2015).

10. Klahr, B. & Hamann, T. Water Oxidation on Hematite Photoelectrodes: Insight into the Nature of Surface States through In Situ Spectroelectrochemistry. *J. Phys. Chem. C* **118,** 10393–10399 (2014).

11. Klahr, B., Gimenez, S., Fabregat-Santiago, F., Hamann, T. & Bisquert, J. Water oxidation at hematite photoelectrodes: the role of surface states. *J. Am. Chem. Soc.* **134,** 4294–4302 (2012).

12. Klahr, B., Gimenez, S., Fabregat-Santiago, F., Bisquert, J. & Hamann, T. W. Electrochemical and photoelectrochemical investigation of water oxidation with hematite electrodes. *Energy Environ. Sci.* **5,** 7626 (2012).

13. Mesa, C. A. *et al.* Multihole water oxidation catalysis on haematite photoanodes revealed by operando spectroelectrochemistry and DFT. *Nat. Chem.* **12,** 82–89 (2020).

14. Zhang, M., De Respinis, M. & Frei, H. Time-resolved observations of water oxidation intermediates on a cobalt oxide nanoparticle catalyst. *Nat. Chem.* **6,** 362–367 (2014).

15. Cowan, A. J. *et al.* Activation energies for the rate-limiting step in water photooxidation by nanostructured α-Fe_2O_3 and TiO_2. *J. Am. Chem. Soc.* **133,** 10134–10140 (2011).

16. Pendlebury, S. R. *et al.* Dynamics of photogenerated holes in nanocrystalline α-Fe_2O_3 electrodes for water oxidation probed by transient absorption spectroscopy. *Chem. Commun.* **47,** 716–718 (2011).

17. Pendlebury, S. R. *et al.* Correlating long-lived photogenerated hole populations with photocurrent densities in hematite water oxidation photoanodes. *Energy Environ. Sci.* **5,** 6304–6312 (2012).

18. Barroso, M., Pendlebury, S. R., Cowan, A. J. & Durrant, J. R. Charge carrier trapping, recombination and transfer in hematite (α-Fe_2O_3) water splitting photoanodes. *Chem. Sci.* **4,** 2724–2734 (2013).

19. Pendlebury, S. R. *et al.* Ultrafast charge carrier recombination and trapping in hematite photoanodes under applied bias. *J. Am. Chem. Soc.* **136,** 9854–9857 (2014).

20. Le Formal, F. *et al.* Back electron-hole recombination in hematite photoanodes for water splitting. *J. Am. Chem. Soc.* **136,** 2564–2574 (2014).

21. Meier, K. K. *et al.* A Long-Lived Fe(III)-(Hydroperoxo) Intermediate in the Active H200C Variant of Homoprotocatechuate 2,3-Dioxygenase: Characterization by Mössbauer, Electron Paramagnetic Resonance, and Density Functional Theory Methods. *Inorg. Chem.* **54,** 10269–10280 (2015).

22. Shiraiwa, M. *et al.* The role of long-lived reactive oxygen intermediates in the reaction of ozone with aerosol particles. *Nat. Chem.* **3,** 291–295 (2011).

23. Zhang, M. & Frei, H. Water Oxidation Mechanisms of Metal Oxide Catalysts by Vibrational Spectroscopy of Transient Intermediates. *Annu. Rev. Phys. Chem.* **68,** 209–231 (2017).

24. Chatman, S., Zarzycki, P., Preočanin, T. & Rosso, K. M. Effect of surface site interactions on potentiometric titration of hematite (α-Fe_2O_3) crystal faces. *J. Colloid Interface Sci.* **391,** 125–134 (2013).

25. Chatman, S., Zarzycki, P. & Rosso, K. M. Surface potentials of (001), (012), (113) hematite (α-Fe_2O_3) crystal faces in aqueous solution. *Phys. Chem. Chem. Phys.* **15,** 13911–13921 (2013).

26. Tombácz, E. pH-dependent surface charging of metal oxides. *Per. Pol. Chem. Eng.* **53,** 77 (2009).

27. Nakabayashi, Y. & Nosaka, Y. The pH dependence of OH radical formation in photo-electrochemical water oxidation with rutile TiO_2 single crystals. *Phys. Chem. Chem. Phys.* **17,** 30570–30576 (2015).

28. Peter, L. M. Energetics and kinetics of light-driven oxygen evolution at semiconductor electrodes. The example of hematite. *J. Solid State Electrochem.* **17,** 315–326 (2013).

29. Le Formal, F., Sivula, K. & Grätzel, M. The Transient Photocurrent and Photovoltage Behavior of a Hematite Photoanode under Working Conditions and the Influence of Surface Treatments. *J. Phys. Chem. C* **116,** 26707–26720 (2012).

30. Dabirian, A. & van de Krol, R. High-Temperature Ammonolysis of Thin Film Ta_2O_5 Photoanodes. Evolution of Structural, Optical, and Photoelectrochemical Properties. *Chem. Mater.* **27,** 708–715 (2015).

31. Kecsenovity, E. *et al.* Enhanced Photoelectrochemical Performance of Cuprous Oxide/Graphene Nanohybrids. *J. Am. Chem. Soc.* **139,** 6682–6692 (2017).

32. Li, J. *et al.* Dynamic Role of Cluster Cocatalysts on Molecular Photoanodes for Water Oxidation. *J. Am. Chem. Soc.* **141,** 12839–12848 (2019).

33. Zhang, Y. *et al.* Pivotal Role and Regulation of Proton Transfer in Water Oxidation on Hematite Photoanodes. *J. Am. Chem. Soc.* **138,** 2705–2711 (2016).

34. Abrantes, L. M. & Peter, L. M. Transient photocurrents at passive iron electrodes. *J. Electroanal. Chem.* **150,** 593–601 (1983).

35. Ahn, H.-J., Kwak, M.-J., Lee, J.-S., Yoon, K.-Y. & Jang, J.-H. Nanoporous hematite structures to overcome short diffusion lengths in water splitting. *J. Mater. Chem. A* **2,** 19999–20003 (2014).

36. Zhang, Z. *et al.* Interfacial oxygen vacancies yielding long-lived holes in hematite mesocrystal-based photoanodes. *Nat. Commun.* **10,** 4832 (2019).

37. Upul Wijayantha, K. G., Saremi-Yarahmadi, S. & Peter, L. M. Kinetics of oxygen evolution at α-Fe_2O_3 photoanodes. A study by photoelectrochemical impedance spectroscopy. *Phys. Chem. Chem. Phys.* **13,** 5264–5270 (2011).

38. Iandolo, B. & Hellman, A. The role of surface states in the oxygen evolution reaction on hematite. *Angew. Chem. Int. Ed.* **53,** 13404–13408 (2014).

39. Young, K. M. H., Klahr, B. M., Zandi, O. & Hamann, T. W. Photocatalytic water oxidation with hematite electrodes. *Catal. Sci. Technol.* **3,** 1660 (2013).

40. Kafizas, A. *et al.* Water Oxidation Kinetics of Accumulated Holes on the Surface of a TiO_2 Photoanode. A Rate Law Analysis. *ACS Catal.* **7,** 4896–4903 (2017).

41. Ma, Y. *et al.* Rate Law Analysis of Water Oxidation and Hole Scavenging on a $BiVO_4$ Photoanode. *ACS Energy Lett.* **1,** 618–623 (2016).

42. Ling, Y., Wang, G., Wheeler, D. A., Zhang, J. Z. & Li, Y. Sn-doped hematite nanostructures for photoelectrochemical water splitting. *Nano Lett.* **11,** 2119–2125 (2011).

43. Annamalai, A. *et al.* Activation of Hematite Photoanodes for Solar Water Splitting: Effect of FTO Deformation. *J. Phys. Chem. C* **119,** 3810–3817 (2015).

44. Ma, H. *et al.* Highly self-diffused Sn doping in α-Fe_2O_3 nanorod photoanodes initiated from β-FeOOH nanorod/FTO by hydrogen treatment for solar water oxidation. *Nanoscale* **10,** 22560–22571 (2018).

45. Quitério, P. *et al.* Photoelectrochemical Water Splitting: Thermal Annealing Challenges on Hematite Nanowires. *J. Phys. Chem. C* **124,** 12897–12911 (2020).

46. Wang, D. *et al.* Hierarchical three-dimensional branched hematite nanorod arrays with enhanced mid-visible light absorption for high-efficiency photoelectrochemical water splitting. *Nanoscale* **8,** 12697–12701 (2016).
